# DNA methylation GrimAge strongly predicts lifespan and healthspan

**DOI:** 10.18632/aging.101684

**Published:** 2019-01-21

**Authors:** Ake T. Lu, Austin Quach, James G. Wilson, Alex P. Reiner, Abraham Aviv, Kenneth Raj, Lifang Hou, Andrea A. Baccarelli, Yun Li, James D. Stewart, Eric A. Whitsel, Themistocles L. Assimes, Luigi Ferrucci, Steve Horvath

**Affiliations:** 1 Department of Human Genetics, David Geffen School of Medicine, University of California Los Angeles, Los Angeles, CA 90095, USA; 2 Department of Physiology and Biophysics, University of Mississippi Medical Center, Jackson, MS 39216, USA; 3 Public Health Sciences Division, Fred Hutchinson Cancer Research Center, Seattle, WA 98109, USA; 4 Center of Development and Aging, New Jersey Medical School, Rutgers State University of New Jersey, Newark, NJ 07103, USA; 5 Radiation Effects Department, Centre for Radiation, Chemical and Environmental Hazards, Public Health England, Chilton, Didcot, Oxfordshire, OX11 0RQ, United Kingdom; 6 Center for Population Epigenetics, Robert H. Lurie Comprehensive Cancer Center and Department of Preventive Medicine, Northwestern University Feinberg School of Medicine, Chicago, IL 60611, USA; 7 Laboratory of Environmental Epigenetics, Departments of Environmental Health Sciences Epidemiology, Columbia University Mailman School of Public Health, New York, NY 10032, USA; 8 Departments of Genetics, Biostatistics, Computer Science, University of North Carolina, Chapel Hill, NC 27599, USA; 9 Department of Epidemiology, Gillings School of Global Public Health, University of North Carolina, Chapel Hill, NC 27599, USA; 10 Department of Medicine, School of Medicine, University of North Carolina, Chapel Hill, NC 27516, USA; 11 Department of Medicine (Division of Cardiovascular Medicine), Stanford University School of Medicine, Stanford, CA 94305, USA; 12 VA Palo Alto Health Care System, Palo Alto, CA 94304, USA; 13 Longitudinal Studies Section, Translational Gerontology Branch, National Institute on Aging, National Institutes of Health, USA, Baltimore, MD 21224, USA; 14 Department of Biostatistics, Fielding School of Public Health, University of California Los Angeles, Los Angeles, CA 90095, USA

**Keywords:** DNA methylation GrimAge strongly predicts lifespan and healthspan

## Abstract

It was unknown whether plasma protein levels can be estimated based on DNA methylation (DNAm) levels, and if so, how the resulting surrogates can be consolidated into a powerful predictor of lifespan. We present here, seven DNAm-based estimators of plasma proteins including those of plasminogen activator inhibitor 1 (PAI-1) and growth differentiation factor 15. The resulting predictor of lifespan, DNAm GrimAge (in units of years), is a composite biomarker based on the seven DNAm surrogates and a DNAm-based estimator of smoking pack-years. Adjusting DNAm GrimAge for chronological age generated novel measure of epigenetic age acceleration, *AgeAccelGrim*.

Using large scale validation data from thousands of individuals, we demonstrate that DNAm GrimAge stands out among existing epigenetic clocks in terms of its predictive ability for time-to-death (Cox regression P=2.0E-75), time-to-coronary heart disease (Cox P=6.2E-24), time-to-cancer (P= 1.3E-12), its strong relationship with computed tomography data for fatty liver/excess visceral fat, and age-at-menopause (P=1.6E-12). AgeAccelGrim is strongly associated with a host of age-related conditions including comorbidity count (P=3.45E-17). Similarly, age-adjusted DNAm PAI-1 levels are associated with lifespan (P=5.4E-28), comorbidity count (P= 7.3E-56) and type 2 diabetes (P=2.0E-26). These DNAm-based biomarkers show the expected relationship with lifestyle factors including healthy diet and educational attainment.

Overall, these epigenetic biomarkers are expected to find many applications including human anti-aging studies.

## Introduction

DNAm levels have been used to build accurate composite biomarkers of chronological age [1–4]. DNAm-based age (epigenetic age) estimators, include the pan tissue epigenetic clock by Horvath 2013 [1], based on 353 CpGs, and an estimator developed by Hannum 2013 [2], based on 71 CpGs in leukocytes. These estimators predict lifespan after adjusting for chronological age and other risk factors [5–9]. Moreover, they are also associated with a large host of age-related conditions [10–20]. Recently, DNAm-based biomarkers for lifespan (time-to-death due to all-cause mortality) have been developed [21,22]. For example, Zhang et al (2017) combined mortality associated CpGs [21] into an overall mortality risk score, while Levine et al (2018) developed a lifespan predictor, DNAm PhenoAge, by regressing a phenotypic measure of mortality risk on CpGs [22].

Many analytical strategies are available for developing lifespan predictors from DNAm data. The reported single stage approach involves the direct regression of time-to-death (due to all-cause mortality) on DNAm levels. By contrast, the current study employed a novel two-stage procedure: In stage 1, we defined DNAm-based surrogate biomarkers of smoking pack-years and a selection of plasma proteins that have previously been associated with mortality or morbidity. In stage 2, we regressed time-to-death on these DNAm-based surrogate biomarkers. The resulting mortality risk estimate of the regression model is then linearly transformed into an age estimate (in units of years). We coin this DNAm-based biomarker of mortality "DNAm GrimAge" because high values are grim news, with regards to mortality/morbidity risk. Our comprehensive studies demonstrate that DNAm GrimAge stands out when it comes to associations with age-related conditions, clinical biomarkers, and computed tomography data.

## RESULTS

### Overview of the two-stage approach for defining DNAm GrimAge

We constructed the DNAm GrimAge in two-stages. First, we defined surrogate DNAm biomarkers of physiological risk factors and stress factors. These include the following plasma proteins: adrenomedullin, C-reactive protein, plasminogen activation inhibitor 1 (PAI-1), and growth differentiation factor 15 (GDF15) [23,24]. In addition, given that smoking is a significant risk factor of mortality and morbidity, we also used DNAm-based estimator of smoking pack-years. Second, we combined these biomarkers into a single composite biomarker of lifespan, DNAm GrimAge, which is expressed in units of years. We then performed a large-scale meta-analysis (involving more than 7000 Illumina array measurements), showing that DNAm GrimAge is a better predictor of lifespan than currently available DNAm-based predictors.

Our studies reveal a surprising finding; which is that in some instances, the DNAm-based surrogate biomarkers (e.g. for smoking pack-years) is a better predictors of mortality than the actual observed (self-reported) biomarker. We also correlated DNAm GrimAge with lifestyle factors and a host of age-related conditions, e.g. we demonstrate that these DNAm-based biomarkers predict time to cardiovascular disease. Finally, we show that DNAm GrimAge is also associated with age-related changes in blood cell composition and leukocyte telomere length.

### Training and test data from the Framingham Heart Study

We began by correlating the levels of 88 plasma protein variables (measured using immunoassays) with DNAm array data generated from the same blood samples of n=2,356 individuals from the Framingham heart study (FHS) Offspring Cohort [25] (Supplementary Note 1). We divided the FHS data randomly into a training set (70% of the FHS pedigrees, N= 1731 individuals from 622 pedigrees) and a test data set (30% pedigrees, N=625 individuals from 266 pedigrees, Supplementary Table 1). The mean age of individuals donating DNA for the training set was 66 years, while that of individuals in the test dataset was 67. These participants had similar demographic profiles, smoking history, and number of years’ follow-up as those in the training set (Supplementary Table 1).

### Stage 1: DNAm-based surrogate biomarkers of plasma proteins and smoking pack-years

**Figure 1:**
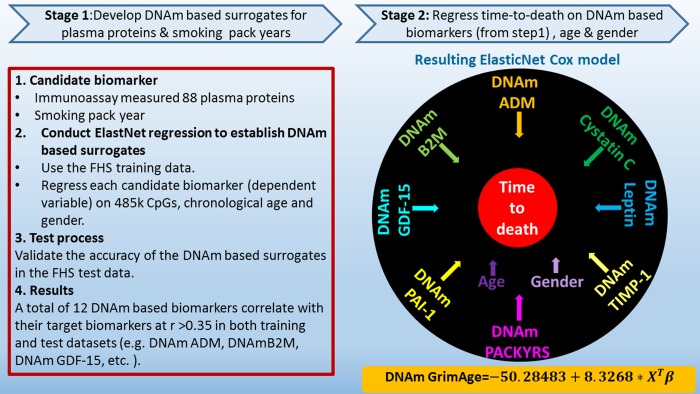
**Flowchart for developing DNAm GrimAge.** Surrogate DNAm-based biomarkers for smoking pack-years and plasma protein levels were defined and validated using training and test data from the Framingham Heart study (stage 1). Only 12 out of 88 plasma proteins exhibited a correlation r >0.35 with their respective DNAm-based surrogate marker in the test data. In stage 2, time-to-death (due to all-cause mortality) was regressed on chronological age, sex, and DNAm-based biomarkers of smoking pack-years and the 12 above mentioned plasma protein levels. The elastic net regression model automatically selected the following covariates: chronological age (Age), sex (Female), and DNAm based surrogates for smoking pack-years (DNAm PACKYRS), adrenomedullin levels (DNAm ADM), beta-2 microglobulin (DNAm B2M), cystatin C (DNAm Cystatin C), growth differentiation factor 15 (DNAm GDF-15), leptin (DNAm Leptin), plasminogen activation inhibitor 1 (DNAm PAI-1), tissue inhibitor metalloproteinase 1 (DNAm TIMP-1). The linear combination of the covariate values X^T^β was linearly transformed to be in units of years. Technically speaking, DNAm GrimAge is a mortality risk estimator. Metaphorically speaking, it estimates biological age.

We used the training data to define DNAm-based surrogate markers of 88 plasma protein variables and smoking pack-years. We restricted the analysis to CpGs that are present on both the Illumina Infinium 450K array and the new Illumina EPIC methylation array in order to ensure future compatibility. Each of the 88 plasma protein variables (dependent variable) was regressed on chronological age, sex, and the CpGs levels in the training data using an elastic net regression model [26], which automatically selected a subset of CpGs (typically fewer than 200 CpGs) whose linear combination best predicted the corresponding plasma level in the training data (Methods). For example, the DNAm levels of 137 CpGs and 211 CpGs allowed us to estimate the plasma levels of GDF15 and PAI-1, respectively. The predicted DNAm values of GDF15 and PAI-1 can then be used as surrogate markers for the measured plasma levels. In general, we denote DNAm-based surrogate markers of plasma proteins and smoking pack-years by adding the prefix "DNAm" to the respective variable name, e.g. DNAm pack-years (Fig. 1 and Supplementary Table 2).

Not all of the available 88 plasma protein levels were successfully imputed based on DNAm data.

Instead, only 12 of the 88 plasma proteins exhibited a moderately high correlation coefficient (r>0.35) between their measured levels and their respective DNAm-based surrogate marker in the test data set (Table 1). We focused on these 12 DNAm surrogate biomarkers in stage 2. Additionally, we constructed a DNAm-based surrogate of self-reported smoking pack-years, DNAm pack-years, based on a linear combination of 172 CpGs.

**Table 1:** Reproducibility and age correlations of DNAm based surrogate biomarkers.

**Correlation ( *r* )**	**Training** **(N=1731)**			**Test** **(N=625)**	
	**Observed** **biomarker**	**Age**		**Observed** **biomarker**	**Age**
**DNAm based surrogate**					
adrenomedullin	0.65	0.63		0.38	0.64
beta-2-microglobulin	0.62	0.83		0.43	0.85
CD56	0.86	0.17		0.36	0.17
ceruloplasmin	0.56	0.04		0.49	-0.02
cystatin-C	0.58	0.81		0.39	0.83
EGF fibulin-like ECM protein1	0.59	0.72		0.41	0.87
growth differentiation factor 15	0.74	0.71		0.53	0.81
leptin	0.68	0.06		0.35	0.05
myoglobin	0.50	-0.04		0.38	0.03
plasminogen activator inhibitor 1	0.69	0.19		0.36	0.16
serum paraoxonase/arylesterase 1	0.57	-0.22		0.51	-0.22
tissue Inhibitor Metalloproteinases 1	0.43	0.92		0.35	0.90
smoking pack-years	0.79	0.17		0.66	0.13

The table reports the correlation coefficients between the observed marker (i.e. observed plasma protein level or self-reported smoking pack-years) and its respective DNAm-based surrogate marker in 1) the FHS training data and 2) the FHS test data. Each of the DNA-based surrogate biomarkers (rows) leads to a correlation *r* > 0.35 in both training and test datasets (columns 2 and 4). DNAm-based pack-years is highly correlated with the self-report pack-years in both training and test datasets (*r* ≥ 0.66). The table also reports the correlation coefficients between the DNAm-based surrogate biomarkers (rows) and chronological age in the FHS training and test data (columns 3 and 5).

### Stage 2: Constructing a composite biomarker of lifespan based on surrogate biomarkers

In stage 2, we developed a predictor of mortality by regressing time-to-death due to all-cause mortality (dependent variable) on the following covariates: the DNAm-based estimator of smoking pack-years, chronological age at the time of the blood draw, sex, and the 12 DNAm-based surrogate biomarkers of plasma protein levels. The elastic net Cox regression model automatically selected the following covariates: DNAm pack-years, age, sex, and the following 7 DNAm-based surrogate markers of plasma proteins: adrenomedullin (ADM), beta-2-microglobulim (B2M), cystatin C (Cystatin C), GDF-15, leptin (Leptin), PAI-1, and tissue inhibitor metalloproteinases 1 (TIMP-1), (Supplementary Table 2). DNAm-based biomarkers for smoking pack-years and the 7 plasma proteins are based on fewer than 200 CpGs each, totaling 1,030 unique CpGs (Supplementary Table 2). Details on the plasma proteins can be found in Supplementary Note 2.

The linear combination of covariates resulting from the elastic net Cox regression model can be interpreted as an estimate of the logarithm of the hazard ratio of mortality. We linearly transformed this parameter into an age estimate, i.e., DNAm GrimAge, by performing a linear transformation whose slope and intercept terms were chosen by forcing the mean and variance of DNAm GrimAge to match that of chronological age in the training data (Methods, Fig. 1). In independent test data, DNAm GrimAge is calculated without estimating any parameter because the numeric values of all parameters were chosen in the training data. Following the terminology from previous articles on DNAm-based biomarkers of aging, we defined a novel measure of epigenetic age acceleration, AgeAccelGrim, which, by definition, is *not* correlated (r=0) with chronological age. Toward this end, we regressed DNAm GrimAge on chronological age using a linear regression model and defined AgeAccelGrim as the corresponding raw residual (i.e. the difference between the observed value of DNAm GrimAge minus its expected value). Thus, a positive (or negative) value of AgeAccelGrim indicates that the DNAm GrimAge is higher (or lower) than expected based on chronological age.

Unless indicated otherwise, we used AgeAccelGrim (rather than DNAm GrimAge) in association tests of age-related conditions because age was a confounder in these analyses. For the same reason, we also used age-adjusted versions of our DNA-based surrogate markers (for smoking pack-years and the seven plasma protein levels). In general, all association tests were adjusted for chronological age and, when required, other confounders as well (such as sex, Methods).

### Pairwise correlations between DNAm GrimAge and surrogate biomarkers

**Figure 2:**
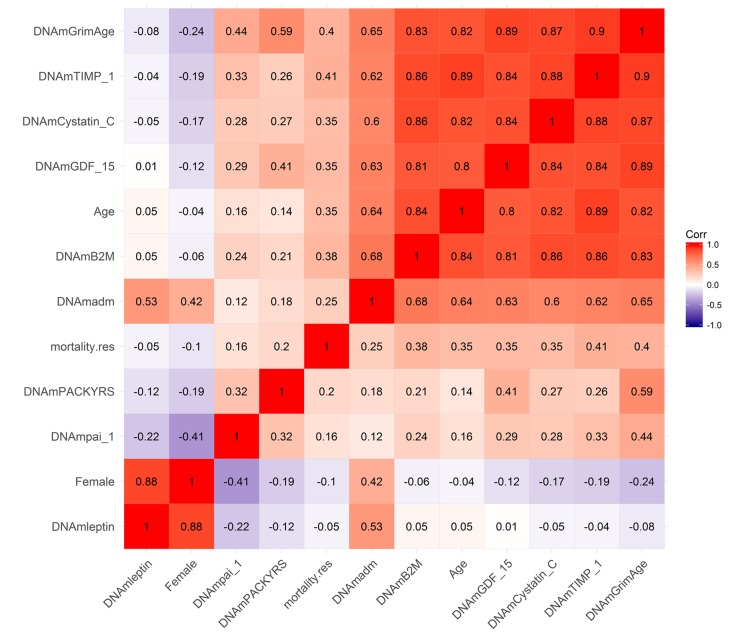
**Heat map of pairwise correlations of DNAm based biomarkers.** The heat map color-codes the pairwise Pearson correlations of select variables (surrounding the definition of DNAm GrimAge) in the test data from the Framingham Heart Study (N=625). DNAm GrimAge is defined as a linear combination of chronological age (Age), sex (Female takes on the value 1 for females and 0 otherwise), and eight DNAm-based surrogate markers for smoking pack-years (DNAm PACKYRS), adrenomedullin levels (DNAm ADM), beta-2 microglobulin (DNAm B2M), cystatin C (DNAm Cystatin C), growth differentiation factor 15 (DNAm GDF-15), leptin (DNAm Leptin), plasminogen activation inhibitor 1 (DNAm PAI-1), issue inhibitor metalloproteinase 1 (DNAm TIMP-1). The figure also includes an estimator of mortality risk, *mortality.res*, which can be interpreted as a measure of "excess" mortality risk compared to the baseline risk in the test data. Formally, mortality.res is defined as the deviance residual from a Cox regression model for time-to-death due to all-cause mortality. The rows and columns of the Figure are sorted according to a hierarchical clustering tree. The shades of color (blue, white, and red) visualize correlation values from -1 to 1. Each square reports a Pearson correlation coefficient.

Using the test data from the FHS, we calculated pairwise correlations between DNAm GrimAge and its underlying variables (Fig. 2 and Supplementary Table 2). DNAm GrimAge is highly correlated with DNAm TIMP-1 (r=0.90) and chronological age (r=0.82). An estimate of excess mortality risk (called mortality residual *mortality.res*) exhibits higher positive correlations with both DNAm GrimAge and DNAm TIMP-1 (*r* ~ 0.40) than with chronological age (*r* ~ 0.35, Fig. 2), in keeping with our later finding that these DNAm biomarkers are better predictors of lifespan than chronological age. With the exception of DNAm Leptin, all of the DNAm-based biomarkers exhibited positive correlations with the measure of excess mortality risk (0.41 ≥ *r ≥* 0.16, Fig. 2). With the exception of DNAm Leptin, all DNAm based surrogate biomarkers exhibited moderate to strong pairwise correlations with each other. DNAm Leptin is elevated in females (Supplementary Fig. 1A, B) consistent with what has been reported in the literature [27,28]. After stratifying by sex, we find that plasma leptin levels increase weakly with age (*r*=0.18 and P=2.1E-3 in males; *r*=0.19, P=4.8E-4 in females, Supplementary Fig. 1E, F).

### Predicting time-to-death in validation data

To evaluate whether our novel DNAm-based biomarkers are better predictors of lifespan than chronological age, we analyzed N=7,375 Illumina methylation arrays generated from blood samples of 6,935 individuals comprising 3 ethnic/racial groups: 50% European ancestry (Caucasians), 40% African Americans, and 10% Hispanic ancestry (Table 2, Methods, Supplementary Note 1). The data came from different cohort studies: test data from the FHS, BA23 and EMPC study from the Women’s Health Initiative (WHI), the InCHIANTI cohort study, and African Americans from the Jackson Heart Study (JHS). We stratified each cohort by race/ethnicity (resulting in 9 strata) to avoid confounding and to ascertain whether the mortality predictors apply to each group separately.

**Table 2:** Overview of the cohorts used in the validation analysis.

				**Smoking status**				
**Study**	**N**	**Female**	**Age**	**Never**	**Former**	**Current**	**Pack-years**	**Years of** **Follow-up**
FHS* test	625	53%	66.9±8.64 [61,73]	37%	52%	10%	14.7±19.91 [0,23]	7.7±1.78 [7.3,8.8]
WHI BA23	2107	100%	65.3±7.1 [60,70.9]	52%	36%	10%	9.5±18.55 [0,12.5]	16.9±4.63 [15.8,19.9]
WHI EMPC	1972	100%	63.3±7.03 [57.9,68.7]	52%	38%	9%	9±17.27 [0,12.5]	18±4.02 [17.9,20.1]
JHS	1747	63%	56.2±12.31 [46.5,65.4]	65%	21%	14%	NA	11.7±2.55 [11.2,13.1]
InChianti**	924 (484)	54%	67±16.64 [60,78]	57%	29%	14%	10.3±17.33 [0,16.8]	5.4±4.84 [0.1,9.3]

NA=not available.

Quantitative variables are presented in the format of mean ±SD [25^th^, 75^th^].

*The distribution of age is based on exam 8.

**The statistics are based on the number of 924 observations across 484 individuals.

The table summarizes the characteristics of 6,935 individuals (corresponding to 7,375 Illumina arrays) from five independent cohorts that were used in our validation analysis. For example, up to two longitudinal measurements were available for each of 484 individuals in the InChianti cohort.

The mean chronological age at the time of the blood draw was 63.0 years. The mean follow-up time (used for assessing time-to-death due to all-cause mortality) was 13.7 years. Since chronological age is one of the component variables underlying DNAmGrimAge, it is not surprising that the latter is highly correlated with age in each of the study cohorts (*r* **≥** 0.79**,** Supplementary Fig. 2).

**Figure 3:**
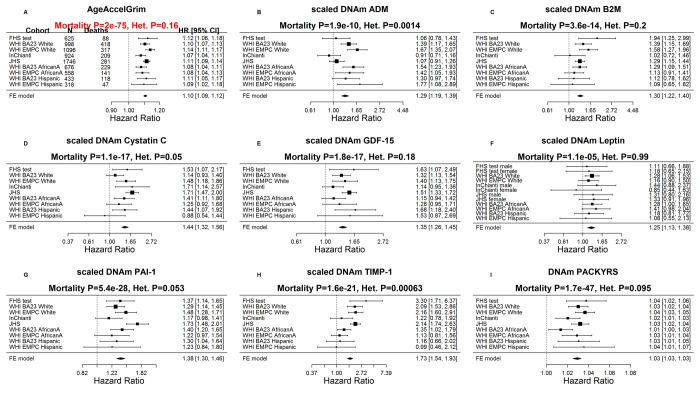
**Meta analysis forest plots for predicting time-to-death due to all-cause mortality.** Each panel reports a meta-analysis forest plot for combining hazard ratios predicting time-to-death based on a DNAm-based biomarker (reported in the figure heading) across different strata formed by racial group within cohort. (**A**) Results for AgeAccelGrim. Each row reports a hazard ratio (for time-to-death) and a 95% confidence interval resulting from a Cox regression model in each of 9 strata (defined by cohort and racial groups). Results for (age-adjusted) DNAm-based surrogate markers of (**B**) adrenomedullin (ADM), (**C**) beta-2 microglobulin (B2M), (**D**) cystatin C (Cystatin C), (**E**) growth differentiation factor 15 (GDF-15), (**F**) leptin, (**G**) plasminogen activation inhibitor 1 (PAI-1), (**H**) tissue inhibitor metalloproteinase 1 (TIMP-1) and (**I**) smoking pack-years (PACKYRS). The sub-title of each panel reports the meta-analysis p-value and a p-value for a test of heterogeneity Cochran Q test (Het.). (**A**) Each hazard ratio (HR) corresponds to a one-year increase in AgeAccelGrim. (**B-H**) Each hazard ratio corresponds to an increase in one-standard deviation. (**I**) Hazard ratios correspond to a 1 year increase in pack-years. The most significant meta-analysis P value (here AgeAccelGrim) is marked in red. A *non-*significant Cochran Q test p-value is desirable because it indicates that the hazard ratios do not differ significantly across the strata. For example, the hazard ratios associated with AgeAccelGrim exhibit insignificant heterogeneity across the strata (${Cochran\;Q\;test\;P}_{I^{2}}$=0.16).

While each (age-adjusted) component variable underlying DNAm GrimAge is a significant predictor of lifespan (Fig. 3), DNAm pack-years (meta-analysis P=1.7E-47) and DNAm PAI-1(P=5.4E-28) exhibit the most significant meta-analysis P-values. The fixed effects meta-analysis P-values reveal that AgeAccelGrim stands out when it comes to lifespan prediction (meta-analysis P=2.0E-75, Fig. 3A). The same applies when the analysis is restricted to never-smokers (Supplementary Fig. 3) or to former/current smokers (Supplementary Fig. 4). AgeAccelGrim remains a highly significant predictor of lifespan after restricting the analysis to never-smokers (N=3,988, meta analysis P=1.1E-16, Supplementary Fig. 9) or to former/current smokers (P=3.5E-33, Supplementary Fig. 3A).

### Instances in which DNAm-based surrogates outperform observed biomarkers

The DNAm-based surrogate biomarker for smoking pack-years has two surprising properties. First, it predicts lifespan in never-smokers (P=1.6E-6, Supplementary Fig. 3I). Second, the surrogate marker is a more significant predictor of lifespan than self-reported pack-years: P=8.5E-5 for DNAm marker versus P=2.1E-3 for observed pack-years in in the FHS test data; similarly, P=5.3E-4 versus 0.18 in the InChianti Study (Supplementary Table 3). The superior predictive performance of DNAm based surrogate biomarkers vis-à-vis their observed/ counter parts also applies to PAI-1 plasma levels (P=8.7E-4 for the DNAm marker versus P=0.074 for the observed levels), TIMP-1 (P=3.8E-4 for the DNAm marker versus P=0.017), and to a lesser extent to cystatin C (P=0.019 for the DNAm estimator versus P=0.054 for the observed level, Supplementary Table 4).

### Mortality prediction based on observed plasma protein levels

The AgeAccelGrim is a composite biomarker derived from DNAm-based surrogate biomarkers of plasma protein levels and smoking pack-years. This begs the question whether a predictor of lifespan based directly on observed plasma protein levels and self-reported smoking pack-years, would outperform its DNAm-based analog? Analogous to our construction of DNAm GrimAge, we used a Cox regression model to regress time to-death on the observed plasma protein levels and self-reported pack-year in the training data (Methods). The resulting mortality risk estimator (defined as weighted average of the observed biomarkers) was linearly transformed into units of years. The resulting predictor, i.e., *observed* GrimAge, and its age-adjusted version. i.e., *DNAm* based AgeAccelGrim, were compared in the FHS, showing similar HRs (*observed* AgeAccelGrim HR=1.10, P=3.2E-7; DNAm based AgeAccelGrim HR= 1.12, P=8.6E-5, Supplementary Table 5). Overall, this comparison shows that DNAm levels in general and our DNAm-based surrogate biomarkers in particular capture a substantial proportion of the information that is captured by the 7 selected plasma proteins and self-reported smoking pack-years. Since our study focuses on DNAm-based biomarkers, we will only consider DNAm-based biomarkers in the following.

### Age-related conditions

**Figure 4:**
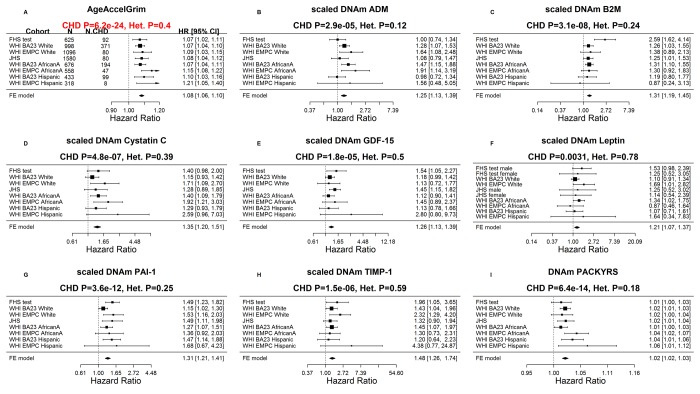
**Meta analysis forest plots for predicting time-to-coronary heart disease.** Each panel reports a meta-analysis forest plot for combining hazard ratios predicting time to CHD and the DNAm-based biomarker (reported in the figure heading) across different strata formed by racial groups within cohorts. (**A**) Results for AgeAccelGrim. Each row reports a hazard ratio (for time-to-CHD) and a 95% confidence interval resulting from a Cox regression model in each of 9 strata (defined by cohort and racial groups). Results for (age adjusted) DNAm-based surrogate markers of (**B**) adrenomedullin (ADM), (**C**) beta-2 microglobulin (B2M), (**D**) cystatin C (Cystatin C), (**E**) growth differentiation factor 15 (GDF-15), (**F**) leptin, (**G**) plasminogen activation inhibitor 1 (PAI-1), (**H**) tissue inhibitor metalloproteinase 1 (TIMP-1) and (**I**) smoking pack-years (PACKYRS). The sub-title of each panel reports the meta-analysis p-value and a p-value for a test of heterogeneity Cochran Q test (Het.). (**A**) Each hazard ratio (HR) corresponds to a one-year increase in AgeAccelGrim. (**B**-**H**) Each hazard ratio corresponds to an increase in one-standard deviation. (**I**) Hazard ratios correspond to a one unit increased in DNAm pack-years. The most significant meta-analysis P value (here AgeAccelGrim) is marked in red.

Our Cox regression analysis of time-to-coronary heart disease (CHD), reveals that AgeAccelGrim is highly predictive of incident CHD (HR=1.07, P=6.2E-24 and $P_{I^{2}}$=0.4, Fig. 4A). As expected, several underlying DNAm-based surrogate biomarkers also individually predict incident CHD; notably the age-adjusted versions of DNAm smoking pack-years (HR=1.02, P=6.4E-14) and DNAm PAI-1 (HR=1.31 per SD, P=3.6E-12).

Similarly, time-to-congestive heart failure (CHF) is also associated with AgeAccelGrim (HR=1.10 and P=4.9E-9), age-adjusted DNAm cystatin C (HR=2.02 and P=2.0E-10) and DNAm PAI-1 (HR=1.58 and P=8.9E-10, Supplementary Fig. 5).

Cross sectional studies reveal that AgeAccelGrim is associated with hypertension (odds ratio [OR]=1.04 and P= 5.1E-13, Supplementary Fig. 6), type 2 diabetes (OR=1.02 and P=0.01, Supplementary Fig. 7), and physical functioning (Stouffer P=1.7E-8, Supplementary Fig. 8). All of the reported associations are in the expected directions, e.g. higher values of AgeAccelGrim are associated with lower physical functioning levels. In women, early age at menopause is associated with significantly higher values of AgeAccelGrim (P=1.6E-12, Supplementary Fig. 9A) and to a lesser extent with all of the age-adjusted versions of the DNAm based surrogate markers, notably DNA cystatin C (P=2.2E-6) and DNAm GDF-15 (P=1.3E-5, Supplementary Fig. 9).

### DNAm plasminogen activation inhibitor 1

**Figure 5:**
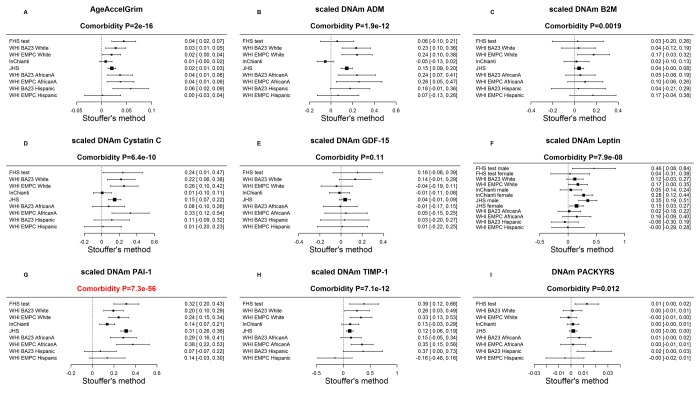
**Meta-analysis of associations with total number of age-related conditions.** Each panel reports a meta-analysis forest plot for combining regression coefficients between the comorbidity index and the DNAm-based biomarker (reported in the figure heading) across different strata, which are formed by racial group within cohort. (**A**) Meta analysis of the regression slope between AgeAccelGrim and the comorbidity index. Analogous results for (age-adjusted) DNAm based surrogate markers of (**B**) adrenomedullin (ADM), (**C**) beta-2 microglobulin (B2M), (**D**) cystatin C (Cystatin C), (**E**) growth differentiation factor 15 (GDF-15), (**F**) leptin, (**G**) plasminogen activation inhibitor 1 (PAI-1), (**H**) tissue inhibitor metalloproteinase 1 (TIMP-1) and (**I**) smoking pack-years (PACKYRS). The individual study results were combined using fixed effect meta-analysis (reported in the panel heading). Cochran Q test for heterogeneity across studies (Het.). The effect sizes correspond to one year of age acceleration in panel **A**, one pack-year in panel I and one standard deviation in other panels for DNAm proteins. The estimate with the most significant meta P value is marked in red.

AgeAccelGrimAge outperforms (age-adjusted versions of) DNAm smoking pack-years and the 7 DNAm-based surrogate markers of plasma protein levels individually with regards to prediction of time-to-death or time-to-coronary heart disease (Figs. 3 and 4). However, age-adjusted DNAm PAI-1 outperforms AgeAccelGrim for several age-related traits (Supplementary Figs. 5-9), notably the comorbidity index (defined as the total number of age-related conditions) where Stouffer's meta-analysis P value for DNAm PAI-1 (P=7.3E-56) is more significant than that for AgeAccelGrim (P=2.0E-16, Fig. 5). As with AgeAccelGrim, higher levels of age-adjusted DNAm PAI-1 are associated with hypertension status, type 2 diabetes status, time-to-CHD (Fig. 4), time-to-CHF, and early age at menopause (Supplementary Figs. 5-7 and 9), while lower levels are associated with disease free status (Stouffer P=2.9E-11, Supplementary Fig. 10) and better physical functioning (Stouffer P=1.4E-8, Supplementary Fig. 8).

### Heritability analysis

We used pedigree based polygenic models (Methods) to measure heritability estimates of AgeAccelGrim and the individual biomarkers. There is significant heritability for AgeAccelGrim ($h^{2}$ =0.30, P=0.022) and observed AgeAccelGrim ($h^{2}$ =0.37, P=0.006**,** Supplementary Table 6). Similarly, several of our DNAm-based surrogate biomarkers (PAI1, B2M, ADM, and GDF15) and their observed counterparts are also highly heritable (Supplementary Table 6), e.g. DNAm PAI-1 ($h^{2}$ =0.34 and P=7.1E-3), observed PAI-1 levels ($h^{2}$ =0.51 and P=6.2E-4), DNAm Beta 2 microglobulin levels ($h^{2}$ =0.45 andP=2.4E-3), and observed B2M ($h^{2}$ =0.34 and P=3.3E-3). Overall, these results suggest that many observed and DNAm-based biomarkers are heritable.

### AgeAccelGrim versus other epigenetic measures of age acceleration

Using the same validation datasets (N=7,375 arrays), we compared DNAm GrimAge with three widely-used DNA-based biomarkers of aging: DNAm age estimator based on different somatic tissues by Horvath (2013) [1], the DNAm age estimator based on leukocytes by Hannum (2013) [2] and the DNAm PhenoAge estimator by Levine (2018) [22]. The respective age-adjusted measures of epigenetic age acceleration will be denoted as AgeAccel (or AgeAccelerationResidual), AgeAccelHannum, and AgeAccelPheno following the notation of previous publications. The four epigenetic measures of age acceleration (including AgeAccelGrim) are in units of year. AgeAccelGrim exhibits moderate positive correlations with each of the three alternative measures of epigenetic age acceleration (0.17 ≤ *r* ≤ 0.45, Supplementary Fig. 11) with the strongest correlation with AgeAccelPheno. The relatively weak correlation with Horvath’s pan-tissue clock (r=0.17) probably reflects the fact that DNAm GrimAge was developed exclusively with blood methylation data. It is evident that AgeAccelGrim is superior with respect to meta-analysis P-values for prediction of time-to-death: AgeAccelGrim (P=2.0E-75, HR=1.10), AgeAccel (Meta P=8.9E-5, HR=1.02, Supplementary Fig. 12), AgeAccelHannum (Meta P=6.8E-16, HR=1.04), AgeAccelPheno (Meta P=3.5E-36, HR=1.05). The results remain qualitatively the same after restricting the analysis to never-smokers or former/current smokers (Supplementary Figs. 13 and 14).

Similarly, AgeAccelGrim stands out when comparing individuals in the top 20% percentile of epigenetic age acceleration to those in the bottom 20% percentile (Stouffer meta-analysis P= 6.4E-38, Supplementary Fig. 15), AgeAccelPheno (P=5.7E-21), AgeAccelHannum (P=1.3E-5), and AgeAccel (P=0.17).

When it comes to significant associations with comorbidity index, age-adjusted DNAm PAI-1( $P_{DNAm\;PAI-1}$=7.3E-56, Fig. 5) outperforms all other DNAm-based biomarkers including AgeAccelGrim ($P_{AgeAccelGrim}$ =2.0E-16) and AgeAccelPheno ($P_{AgeAccelPheno}$=7.8E-21, Supplementary Fig. 16).

AgeAccelGrim is more informative than AgeAccelPheno in predicting time-to-CHD ($P_{AgeAccelGrim}$ =6.2E-24 and ${HR}_{AgeAccelGrim}$=1.07 versus $P_{AgeAccelPheno}$= 1.7E-8 and ${HR}_{AgeAccelPheno}$ =1.03, Supplementary Fig. 17) even after stratifying the analysis by smoking status (Supplementary Figs. 18 and 19).

AgeAccelGrim greatly outperforms the other 3 measures of epigenetic age acceleration including predicting time to (any) cancer (AgeAccelGrim P= 1.3E-12 versus AgeAccelPheno P=2.7E-3, Supplementary Fig. 20) and as related to an inverse association with early age at menopause in women (AgeAccelGrim P=1.6E-12 versus AgeAccel P=2.2E-3, Supplementary Fig. 21). A sensitivity analysis reveals that the latter finding remains qualitatively the same even after removing the InChianti cohort, which exhibited the strongest negative association between epigenetic age acceleration and age at menopause (Supplementary Fig. 22).

### Multivariate Cox models adjusting for traditional risk factors

The above-mentioned Cox regression models were adjusted for age at blood draw (baseline), batch, pedigree, and intra-subject correlation as needed. We also fit multivariate Cox regression models that included additional covariates assessed at baseline: body mass index, educational level, alcohol intake, smoking pack-years, prior history of diabetes, prior history of cancer, and hypertension status (Methods). Even after adjusting for these known risk factors for morbidity, AgeAccelGrim remained a highly significant predictor of lifespan (P=5.7E-29, Supplementary Fig. 23) and time-to-CHD (P=3.7E-11, Supplementary Fig. 24) and outperformed previously published measures of epigenetic age acceleration.

### Stratified analyses

We evaluated AgeAccelGrim and underlying DNAm biomarkers in different strata characterized by age (younger/older than 65 years), body mass index (obese versus non-obese), educational attainment, prevalent condition at baseline such as prior history of cancer, type 2 diabetes, or hypertension. In all of these strata, AgeAccelGrim remains a significant predictor of time-to-death (Supplementary Table 7) and time-to-CHD (Supplementary Table 8). Furthermore, AgeAccelGrim outperforms existing DNAm-based biomarkers of aging in all strata except for one (comprised of n=281 individuals with a prior history of cancer).

These subgroup analysis results also confirm that epigenetic age acceleration is an independent predictor of earlier mortality even after adjusting for possible confounders and within major subgroups of the population. Additional results applied to age-adjusted DNAm proteins and DNAm pack-years are listed in Supplementary Data 1. With few exceptions, we found that DNAm-based PAI-1, TIMP-1 and pack-years remained highly significant in each stratum.

### Exceptionally fast/slow agers

The DNAm GrimAge estimate allows an intuitive interpretation as physiological age since it is in units of years. However, if someone is 8 years older than expected, this does not mean that this person has on average a 8 year shorter life expectancy. Rather, one should use the hazard ratio when it comes to assessing mortality risks. It is a statistical co-incidence that the hazard ratio associated with one-year increase in AgeAccelGrim is the same in strata comprised of never-smokers (HR=1.10, Supplementary Fig. 3A), former/current smokers (HR=1.10, Supplementary Fig. 4A), and among all individuals combined (HR=1.10, Fig. 3A). This allows us to evaluate the mortality risks in exceptionally fast and slow agers (according to AgeAccelGrim) irrespective of their smoking status. The top 5^th^ percentile and the 95% percentile of AgeAccelGrim corresponds to -7.5 years and + 8.3 years respectively (Supplementary Table 9). A person in the top 95^th^ percentile of AgeAccelGrim (=8.3 years) faces a hazard of death that is twice that of the average person in their stratum (whose AgeAccelGrim equals 0). Specifically, fast aging status is associated with a hazard ratio of HR=2.2=1.10^8.3^. Conversely, a slow ager in the bottom 5^th^ percentile (-7.5 years) faces a hazard of death that is half that of the average person in their stratum, HR=0.49=1.10^-7.5^.

### DNAm GrimAge versus single stage estimators of mortality risk

DNAm GrimAge was built using a novel two-stage approach that critically depended on the development of DNAm-based surrogate biomarkers. To justify the utility of this indirect approach, we compared DNAm GrimAge with several DNAm-based mortality risk predictors that were developed by directly regressing lifespan on DNAm data (referred to as single stage mortality predictors). To this end, we developed a new mortality predictor, DNAm Mortality (in year units) by directly regressing time-to-death (due to all-cause mortality) on CpGs in the FHS training data. DNAm Mortality was calculated as linear combination of 59 CpGs. The direct approach entailed the constructions of DNAm Mortality, an elastic net Cox regression model, and linear transformation of the mortality risk to ensure that the values of DNAm Mortality are in units of years (Methods). In addition, we also evaluated the published mortality predictor by Zhang [21] which, remarkably, is based on only 10 CpGs (Methods). The latter two (single-stage) lifespan predictors were found to correlate highly with each other (r=0.77 in the FHS test data).

The novel age-adjusted DNAm Mortality estimator (HR=1.07, P=3.0E-44) and both versions of Zhang's mortality risk estimator (P=4.2E-39, Supplementary Fig. 25) lead to a less significant meta-analysis P-value for lifespan prediction than AgeAccelGrim (P=2.0E-75). It is not meaningful to compare HR estimates (here HR=1.02 and HR=1.10, respectively) because these HR estimates critically depend on the scale/distribution of the respective mortality predictors. To provide a meaningful and scale-independent comparison, we focused on the meta-analysis P-values.

AgeAccelGrim also stands out in terms of its meta-analysis P-value for predicting time-to-CHD (AgeAccelGrim P=6.2E-24, AgeAccelMortality P=4.6E-11, AgeAccelZhang P=9.5E-12, Supplementary Fig. 26).

It is useful to characterize the different lifespan predictors in terms of their correlation with DNAm pack-years because smoking is a major risk factor. Age-adjusted DNAm pack-years exhibits positive correlations with both DNAm Mortality and Zhang's mortality predictor (*r* ≥ 0.55). The connection of single stage mortality predictors to smoking can also be observed at the CpG level. DNAm Mortality, Zhang’s mortality predictor, and DNAm pack-years explicitly use CpG cg05575921 (in the *AHRR* gene on chromosome 5p15.33), which has previously been identified by epigenome-wide association studies of cumulative smoking exposure [21,29]. Overall, these results suggest that the two single-stage lifespan predictors relate more strongly to cumulative smoking exposure than does AgeAccelGrim.

### Association with blood cell composition

DNAm data allow one to estimate several quantitative measures of blood cell types as described in Methods [30,31]. We previously showed that DNAm biomarkers of aging, which capture age-related changes in blood cell composition, are better predictors of lifespan than those that are independent of blood cell counts [7]. Therefore, we hypothesized that several of our novel DNAm biomarkers would exhibit significant correlations with these imputed measures of blood cell composition. This is indeed the case as can be seen from our large scale meta-analysis across the validation data (Supplementary Fig. 27, Supplementary Data 2). AgeAccelGrim is significantly associated with a decrease in naive CD8 naïve cells (r=-0.22, P=9.2E-62, Supplementary Fig. 27A and Supplementary Data 2), CD4+T cells (r=-0.21, P=1.8E-57), and B cells (r=-0.18, P=9.7E-43) and with an increase in granulocytes/neutrophils (r=0.24, P=1.5E-74) and plasma blasts (r=0.22, P=7.3E-63). While these results demonstrate that AgeAccelGrim is associated with an age-related decline in immune system functioning, our cross sectional analysis does not allow us to dissect cause-and-effect relationships.

Age-adjusted DNAm TIMP-1 exhibits the most significant correlations with the measures of blood cell composition (e.g. proportion of granulocytes r=0.36, P=2.7E-172, Supplementary Fig. 27H and Supplementary Data 2) followed by age-adjusted DNAm Cystatin C (proportion of CD4+ T cells counts r=-0.33, P=3.4E-142). Although many of our DNAm biomarkers are correlated with blood cell counts, this does not mean that these measures *only* capture changes in blood cell composition as can be seen from the following. First, measures of blood cell composition correlate weakly with our age-adjusted DNAm surrogate markers of smoking pack-years (strongest correlation r=-0.14, Supplementary Fig. 27G) and PAI-1 levels (strongest correlation r=0.17, Supplementary Fig. 27I) even though both biomarkers are strongly associated with mortality risk and age-related conditions as shown above. Second, the DNAm surrogate markers remain significant predictors of mortality in multivariate Cox regression models that include blood cell counts as additional covariates as detailed in the following.

### Cox models that include blood cell counts

Our multivariate Cox regression models demonstrate that AgeAccelGrim remains highly predictive of lifespan (P=2.6E-53) even after adjusting for seven covariates that assess imputed blood cell counts (Supplementary Fig. 28A). Note that this p-value is only slightly lower than that obtained without adjustment for blood cell counts (P=2.0E-75 in Fig. 3A). Further, AgeAccelGrim remains highly predictive for time-to-CHD (OR=1.07, P=1.1E-17 Supplementary Fig. 29A) even after adjusting for blood cell counts.

Similarly, our other DNAm biomarkers (such as DNAm PAI-1, DNAm PACKYRS) remain predictive of lifespan and time-to-CHD after adjusting for blood cell counts (SSupplementary Figs. 28 B-I and 29 B-I ). While this adjustment typically lowers statistical significance levels, there is one notable exception: DNAm leptin levels exhibits *more* significant P values after adjusting for blood cell counts (Supplementary Fig. 28F versus Fig. 3F; Supplementary Fig. 29F versus Fig. 4F).

### Association with leucocyte telomere length

Leukocyte telomere length (LTL) has been found to be weakly predictive of mortality and cardiovascular disease. Our meta-analysis reveals a statistically significant but weak negative correlation between LTL and AgeAccelGrim (*r*= -0.12 and meta P= 3.3E-10, Supplementary Table 10) across data from the FHS, WHI (BA23 sub-study) and JHS (total N =2,702, 27% White and 73% African American). Similarly, LTL exhibits (weak) negative correlations with DNAm based surrogate biomarkers for GDF-15 (*r*= -0.10, meta P= 3.4E-7), DNAm PAI-1 (*r*= -0.10, meta P= 5.1E-8) and DNAm smoking pack-years (*r*= -0.09 and meta P= 2.9E-6).

### Functional annotation of sets of CpGs

The genomic locations of the 1030 CpGs underlying the DNAm GrimAge estimator were analyzed using the GREAT software tool [32] which assigns biological meaning to a set of genomic locations (here CpGs) by analyzing the annotations of nearby genes. At a false discovery rate of FDR < 0.05 we found 361 gene sets from GO, KEGG, PANTHER. Among those, 28 surpassed the more stringent Bonferroni correction including MHC class II receptor activity (nominal P=1.2E-6), cytokine-mediated signaling pathway (P=6.9E-5), response to interferon-gamma (P=1.5e-4), regulation of protein sumoylation (P=4.4E-5), endoderm formation (P=5.9E-5), epigenetic regulation of gene expression (P=6.7E-5), and fatty acid transmembrane transport (P=9.5E-5).

Similarly, we evaluated sets of CpGs underlying DNAm-based surrogate biomarkers. At FDR < 0.05, we found n=388, 307, and 153 significant gene sets for DNAm B2M, PAI-1, and Cystatin-C, respectively. Of those, the top gene sets are involved in immune function (nominal P=1.1E-9 for DNAm B2M CpGs), adipocytokine signaling pathway (P =3.6E-7 for DNAm PAI-1 CpGs) or lipid function (P =3.8E-7 for DNAm PAI-1 CpGs). The significant gene sets for all DNAm surrogate biomarkers can be found in Supplementary Data 3.

### Diet, education, and life style factors

**Figure 6:**
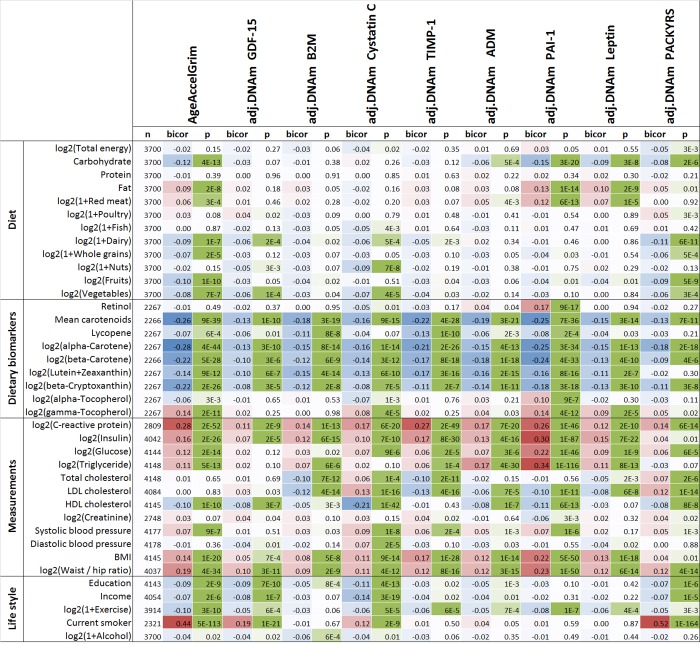
**Cross sectional correlations between DNAm biomarkers and lifestyle factors.** Robust correlation coefficients (biweight midcorrelation [62]) between 1) AgeAccelGrim and its eight age-adjusted underlying DNAm-based surrogate biomarkers and 2) 38 variables including self-reported diet, 9 dietary biomarkers, 12 variables related to metabolic traits and central adiposity, and 5 life style factors. The 2-color scale (blue to red) color-codes bicor correlation coefficients in the range [-1, 1]. The green color scale (light to dark) applied to unadjusted P values. The analysis was performed on the WHI cohort in up to 4200 postmenopausal women. An analogous analysis stratified by race/ethnicity can be found in Supplementary Fig. 30.

Several previous measures of epigenetic age acceleration in blood have been shown to exhibit statistically significant but weak correlations with lifestyle factors and biomarkers of metabolic syndrome [22,33]. Here we revisited these cross-sectional studies in the WHI (comprising approximately 4000 postmenopausal women, Methods) with our novel measures of AgeAccelGrim and its underlying DNAm-based surrogate biomarkers (Fig. 6).

All (age-adjusted) DNAm-based biomarkers correlate with plasma biomarkers measuring vegetable consumption, but AgeAccelGrim (robust correlation coefficient r=-0.26, P=9E-39**,** Fig. 6) and DNAm PAI-1 (r=-0.25, P=7E-36) stand out in terms of their strong relationship with mean carotenoid levels (Fig. 6, Supplementary Fig. 30). Far less significant associations could be observed for self-reported measures of fruit, vegetable, and dairy intake, which highlights the limitations of self-reported measures of dietary intake.

The following novel results could not be observed with previous DNAm-based biomarkers of aging: (self-reported) proportion of carbohydrate consumption was associated with lower AgeAccelGrim (robust correlation r=-0.12, P=4E-13) and DNAm PAI-1 (r=-0.15, P=3E-20). Conversely, an increased proportion of fat intake (but not protein intake) was associated with increased AgeAccelGrim (r=0.09, P=2E-8) and DNAm PAI-1 (r=0.13, P=1E-14). Measures of lipid metabolism, triglyceride levels and HDL cholesterol levels, were significantly correlated with AgeAccelGrim (r=0.11 and r=-0.10, respectively) and even more so with (age adjusted) DNAm PAI-1 levels (r=0.34 and r=-0.11). Similarly, measures of glucose metabolism, insulin- and glucose levels, exhibited positive correlations with AgeAccelGrim (r=0.16 and r=0.12, respectively) and with (age adjusted) DNAm PAI-1 levels (r=0.30 and r=0.22).

Similar to what we observed with previous DNAm based biomarkers of aging, plasma C-reactive protein levels exhibited comparatively strong positive correlations with DNAm-based biomarkers, particularly AgeAccelGrim (r=0.28, P=2E-52), DNAm TIMP-1 (r=0.27, P=2E-49), and DNAm PAI-1 (r=0.26, P=1E-46).

Measures of adiposity, BMI and waist-to-hip ratio, are associated with increased AgeAccelGrim, age-adjusted DNAm PAI-1, and other DNAm-based surrogate biomarkers. Higher education and income are associated with lower AgeAccelGrim (P=2E-9 and P=2E-6). AgeAccelGrim stands out when it comes to detecting a beneficial effect of physical exercise (r=-0.10, P=3E-10).

Several of our results in the WHI could be replicated in a smaller dataset (N< 625 individuals from the FHS test data) that included lipid and metabolic biomarker data (Supplementary Fig. 31). In the FHS, hemoglobin A1C and albumin levels (in urine) exhibited significant positive correlations with AgeAccelGrim, age-adjusted DNAm PAI-1 (0.10 ≤ *r* ≤0.12 and 1.4E-7 ≤ *P* ≤ 2.3E-3), and to a lesser extent with our other DNAm based surrogate biomarkers (Supplementary Fig. 31).

### Omega-3 polyunsaturated fatty acid supplementation

Omega-3 polyunsaturated fatty acid (PUFAs) supplementation is increasingly used for protection against cardiovascular disease. However, omega-3 PUFA supplementation was not found to be associated with a lower risk of cardiac death, sudden death, myocardial infarction, stroke, or all-cause mortality [34–36]. We studied the association between self-reported omega-3 intake and AgeAccelGrim in n=2,174 participants of the FHS and found that omega-3 acids intake was negatively correlated with AgeAccelGrim (robust correlation r=-0.10, P=4.6E-7, linear mixed effects P=1.3E-5, Supplementary Table 11). The effect of omega 3 supplementation is more pronounced in males (r=-0.08, P=0.012) than in females (r=-0.05, P=0.07).

A multivariate linear mixed model analysis revealed an association between AgeAgelGrim and omega-3 acid levels (linear mixed effects P=0.017) after adjusting for gender, educational levels, data status (an indicator of training data), and smoking pack-year.

### Computed tomography measures of fatty organs

Computed tomography (CT) imaging techniques provide "shadow images of fat" that can be used for the indirect quantification of organ quality (e.g. liver). Radiographic pixels measure the density of an organ (referred to as attenuation) in Hounsfield units (HU). CT scans are used for diagnosing fatty liver disease: a low density/attenuation value (low HU) is associated with *high* fat content in the liver.

We analyzed CT scan data from liver, spleen, paraspinal muscle, visceral adipose tissue (VAT), and subcutaneous adipose tissue (SAT) from the Framingham Heart Study cohort [37,38] (Methods).

**Figure 7:**
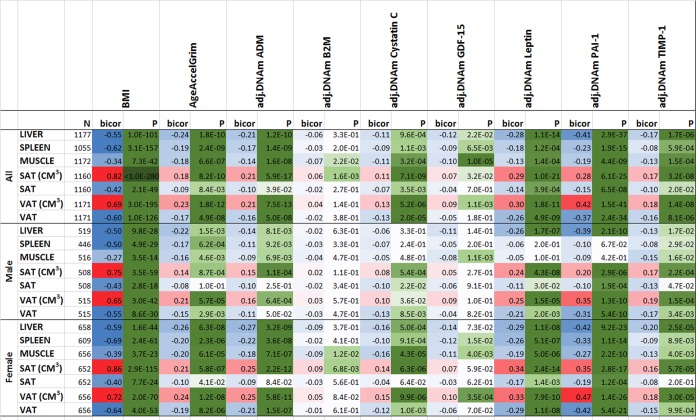
**Computed tomography variables versus with body mass index and age-adjusted DNAm biomarkers in the FHS.** The columns correspond to BMI, AgeAccelGrim and age-adjusted DNAm surrogates of plasma proteins. The rows correspond to computed tomography-derived organ density measures (Hounsfield units) or volumetric measures for subcutaneous adipose tissue (SAT CM3) or visceral adipose tissue (VAT CM3). The columns report the available sample size (n) in the FHS, the robust correlation coefficient (bicor, based on the biweight midcorrelation coefficient [62]). To avoid confounding by pedigree structure, we computed the p-value using a linear mixed effect model (pedigree as random effect). The bicor correlation coefficients are color-coded (blue to red) across its range of [-1, 1]. P-values are color-coded in green (light to dark green scale). We applied the correlation analysis to males and females, respectively, and then combined the results via fixed effect models weighted by inverse variance (listed in the top rows, denoted as “ALL”).

As expected, BMI exhibited strong positive correlations with volumetric measures of SAT (r=0.82, Fig. 7) and VAT (r=0.69). Further, we observed strong negative correlations between body mass index and density (attenuation) values in liver (r=-0.55, p=1E-101, Fig. 7), spleen (r=-0.62,P=3E-157), paraspinal muscle (r=-0.34, P=7E-42), subcutaneous adipose tissue (SAT, r=-0.42, P=2E-49), and visceral adipose tissue (VAT, r=-0.60, P=1E-126). With the exception of muscle, CT values exhibit only weak correlations with chronological age in this cohort comprised of older individuals (Supplementary Fig. 32). We previously found that body mass index is strongly correlated (r=0.42) with epigenetic age acceleration in human liver but exhibits only weak correlations with epigenetic age acceleration in blood (r around 0.10) [39].

Compared to previous epigenetic biomarkers of aging (Supplementary Fig. 33), AgeAccelGrim and DNAm PAI-1 stand out in terms of their strong correlations with CT-derived measures of adiposity (Fig. 7): AgeAccelGrim is negatively correlated with liver density (bicor= -0.24, P=1.79E-10) and positively correlated with VAT volume (bicor=0.23, P=1.77E-12) in both sexes.

Most of our DNAm-based surrogate biomarkers of proteins are significantly associated with CT measures of adiposity (Fig. 7) except for our DNAm-based surrogate biomarkers of B2M and smoking pack-years (which exhibit non-significant correlations after adjusting for multiple comparisons).

The strong marginal correlations between AgeAccelGrim and CT measures beg the question whether they reflect confounding by BMI or sex. This is not the case as can be seen from a multivariate regression model that regressed AgeAccelGrim (dependent variable) on BMI, sex, and several CT derived measures of organ density and fat volume. Even after adjusting for potential confounders, AgeAccelGrim exhibits a significant association with liver density (P=6.86E-4) (Model I in Supplementary Table 12 and Methods). Interestingly, BMI is no longer associated with AgeAccelGrim after adjusting the analysis for liver density or VAT volume (Supplementary Table 12) which suggests that liver density mediates the relationship between BMI and AgeAccelGrim.

A multivariate model analysis reveals that AgeAccelGrim is more strongly associated with VAT volume (P= 5.54E-4) than with SAT volume (Model II in Supplementary Table 12) which supports the widely held view that VAT is more dangerous than SAT.

A comprehensive multivariate model that includes both organ density measures and volumetric measures of SAT/VAT reveals that liver density (P=7.32E-3) exhibits the most significant association with AgeAccelGrim (Model III in Supplementary Table 12).

Age-adjusted DNAm-based surrogate markers of PAI-1, ADM, TIMP-1, and leptin also exhibit significant correlation with the CT measures (Fig. 7). The finding associated with age-adjusted DNAm leptin echoes the earlier significant association between immunoassay based leptin with SAT and VAT variables [37].

Age adjusted DNAm PAI-1 exhibits the strongest associations with CT-based measures of adiposity: it is strongly and positively correlated with VAT volume (r=0.42, P=1.5E-41, Fig. 7), SAT volume (r=0.28) and negatively correlated with liver density (r=-0.41, P=2.9E-37), VAT density (r=-0.37), and spleen density (r=-0.23). A multivariate regression analysis of age-adjusted PAI-1 (dependent variable) reveals highly significant associations with liver density (P=3.17E-14 in Model I) and VAT volume (P=4.22E-13, Model II in Supplementary Table 13) even after adjusting for BMI and other confounders. Including all CT variables as covariates in a multivariate model reveals significant associations with both liver density (P=3.16E-8) and VAT volume (P=1.38E-7, Model III in Supplementary Table 13).

Overall, these results suggest that fatty liver and excess VAT are the most significant CT-based measures of (age-adjusted) DNAm PAI-1 and DNAm Grim.

## DISCUSSION

Several articles have previously described DNAm-based biomarkers for measuring tissue age and for predicting lifespan [10,40]. This work shows that DNAm GrimAge, which is as a linear combination of chronological age, sex, and DNAm-based surrogate biomarkers for seven plasma proteins and smoking pack-years, outperforms all other DNAm-based biomarkers, on a variety of health-related metrics. An age-adjusted version of DNAm GrimAge, which can be regarded as a new measure of epigenetic age acceleration (AgeAccelGrim), is associated with a host of age-related conditions, lifestyle factors, and clinical biomarkers. Using large scale validation data from three ethnic groups, we demonstrate that AgeAccelGrim stands out among pre-existing epigenetic clocks in terms of its predictive ability for time-to-death, time-to-coronary heart disease, time-to-cancer, its association with computed tomography data for fatty liver/excess fat, and early age at menopause.

Our DNAm-based surrogate biomarker of smoking might complement self-reported assessments of pack-years. The surprising finding that DNAm pack-years outperforms self-reported pack-years in predicting lifespan could reflect a) erroneous self-reporting or b) the fact that DNAm pack-years captures intrinsic variation across individuals with lasting biological damage that results from smoking, i.e., inter-individual sensitivities to smoking.

Markers of inflammation and metabolic conditions are associated with several epigenetic biomarkers including AgeAccelGrim, age-adjusted DNAm TIMP-1, and DNAm PAI-1. However, DNAm PAI-1 stands out when it comes to associations with type 2 diabetes status, glucose-, insulin-, triglyceride levels, anthropometric measures of adiposity (body mass index and waist-to-hip ratio), and computed tomography data on fatty liver and excess adipose tissue.

Our DNAm-based surrogate biomarkers of plasma protein levels may be leveraged by researchers who rely on bio-banked DNA samples without the availability of plasma samples. Strong evidence supports links between plasma proteins used in the construction of GrimAge and various age-related conditions: ADM levels are increased in individuals with hypertension and heart failure [41]. Plasma B2M is a clinical biomarker associated with cardiovascular disease, kidney function, and inflammation [42]. Plasma cystatin-C is used to assess kidney function [43]. ADM, B2M, cystatin C, and leptin relate to many age-related traits including cognitive functioning [44–46]. GDF-15 is involved in age-related mitochondrial dysfunction [46]. PAI-1 plays a central role in a number of age-related subclinical and clinical conditions [47], and recent genetic studies link PAI-1 to lifespan [48]. The tissue inhibitor of metalloproteinases, TIMP-1, plays an anti-apoptotic function [49]. We acknowledge the following limitations. The levels of relatively few plasma proteins (12 out of 88) were accurately imputed based on DNAm levels in blood. In the FHS data, the measurement of the plasma proteins (exam 7) preceded the measurement of blood DNAm data (exam 8) by 6.6 years, suggesting that the DNAm profiles may not represent a highly accurate snapshot of the status of these proteins at the time of blood collection. That said, the elucidation of cause-and-effect relationships between plasma proteins and DNAm will require future longitudinal cohort studies and mechanistic evaluations.

Despite their obvious strengths, DNAm-based biomarkers are unlikely to replace existing clinical biomarkers such as blood glucose or blood pressure measurements in medical practice. Rather, these epigenetic biomarkers are expected to complement existing clinical biomarkers when evaluating the individual’s ‘aging’ status. Since DNAm captures important properties of the DNA molecule, these DNAm biomarkers are proximal to innate aging processes [10].

Beyond lifespan prediction, AgeAccelGrim (and several of its underlying surrogate biomarkers including DNAm PAI-1) relate to many age-related conditions (multi-morbidity, metabolic syndrome, markers of inflammation) in the expected way, i.e. high values are associated with a bad risk profile.

In general, epigenetic aging is distinct from senescence-mediated aging and is not prevented by telomerase expression [50–52]. In spite of this, we do find that higher values of AgeAccelGrim (and several DNAm-based surrogate markers) are associated with shorter telomere length and an imputed blood cell composition that is indicative of immunosenescence.

Overall, we expect that these DNAm-based biomarkers will find useful applications in numerous human studies, especially those of anti-aging interventions.

## METHODS

### Study cohort

To establish DNAm based estimators and DNAm GrimAge, we used 2,356 individuals composed of 888 pedigrees from the FHS cohort [25], a large-scale longitudinal study started in 1948, initially investigating risk factors for cardiovascular disease (CVD). The FHS cohort contains medical history and measurements, immunoassays at exam 7, and blood DNA methylation profiling at exam 8. The technology of immunoassay was based on Luminex xMAP assay, an extension of the enzyme-linked immunosorbent assay (ELISA) performed with multiple analyte-specific capture antibodies bound to a set of fluorescent beads. The DNA methylation profiling was based on the Illumina Infinium HumanMethylation450K BeadChip.

We assigned 70% pedigrees (1731 individuals/622 pedigrees) to the training process and the remaining 30% of pedigrees (625 individuals/266 pedigrees) to the FHS test data (Supplementary Table 1). The training dataset was used to build the DNAm based surrogate markers for plasma proteins, smoking pack-years, and the composite biomarker DNAm GrimAge.

### Validation data from 5 cohorts

Our validation analyses involved 7,375 Illumina arrays measuring blood methylation levels in N=6,935 individuals from five independent cohorts: the FHS test dataset (N=625), WHI BA23 (N=2107), WHI EMPC study (N=1972), JHS (N=1747), and InChianti (N=924 from 1 to 2 longitudinal measures on 484 individuals, Table 2 and Supplementary Note 1). All the statistical analyses were adjusted for the correlation structure due to pedigree effects or repeated measurements as described below.

### Estimation of surrogate DNAm based biomarkers

We developed estimators for plasma proteins based on blood methylation data. We leveraged immunoassay measurements in the FHS which profiled 88 plasma protein biomarkers (in units of pg/mL), including cardiovascular disease related plasma proteins such as C-reactive protein [53] and growth differentiation factor 15 (GDF-15) [54]. For each protein marker, missing values were imputed by the respective median value. The median missing rate was < 0.3%. Next the resulting observed plasma levels were regressed on DNAm data in the FHS training data.

Each plasma protein was regressed on the CpGs using the elastic net regression model implemented in the R package *glmnet*. Ten-fold cross validation was performed in the FHS training data to specify the underlying tuning parameter λ. The selection of CpGs by the penalized regression model is not robust. Similar estimator could be built using different sets of CpGs

We required the predicted variable associated with the target variable with >0.35 correlation in both training and test datasets. Only 12 out of 88 proteins exhibited a correlation greater than 0.35 between observed plasma levels and their respective DNAm based estimators in the FHS test data (Table 1). The missing rates of the 12 ImmunoAssay proteins were less than 0.7%. The correlation estimates have a distribution of 0.64±0.12 [0.43, 0.86] (mean±SD [range]) in the training dataset and a distribution of 0.43±0.09 [0.35, 0.66] in the test dataset.

### DNA methylation data

Our study involved DNA methylation data generated on two different Illumina array platforms: Illumina Inf 450K array and the Illumina EPIC array. Our analysis focused on the subset of 450,161 CpGs that were present on both platforms. We used meta analysis techniques to combine the results from the difference cohorts since the respective methylation data were normalized using different methods, e.g. the WHI BA23 were normalized using the background correction method implemented in GenomeStudio. By contrast, the JHS data were normalized using the "noob" normalization method implemented in the *minfi* R package [55,56]. We kept the original normalization methods to ensure consistency with previous publications.

### Smoking Pack-Years

The variable "smoking pack-years" attempts to measure the cumulative amount of cigarettes consumed by the smoker. It is calculated by the number of packs of cigarettes smoked per day multiplied by the number of years the person smoked. We computed smoking pack-years using the information up to exam 8 in the FHS cohort.

### Definition of DNAm GrimAge

We again used an elastic net Cox regression model [26] to regress time-to-death (due to all-cause mortality) since exam 7 on the 12 DNAm based surrogate markers for plasma proteins and on DNAm PACKYR, chronological age, and sex. As part of stage 2, we validated the accuracy of the DNAm based surrogate markers for their observed counterparts in the FHS test dataset. However, mortality predictor (DNAmGrimAge) was only fit in the FHS training dataset (N=1731). In the training dataset, we performed 10-fold cross validation to specify the value of the tuning parameter λ. A completely unbiased evaluation of DNAm GrimAge is achieved in the validation data sets (WHI, JHS, and InChianti).

### Calibration of DNAm GrimAge into units of years

The final elastic net Cox model listed in Supplementary Table 2 results in an uncalibrated DNAm GrimAge estimate, which can be interpreted as the linear combination of the covariates, $X^{T}\beta$ , or alternatively as the logarithm of the hazard ratio,$\log[h\left(t\right){/h}_{0}\left(t\right)]=X^{T}\beta$**,** where $h_{0}\left(t\right)$ is the baseline hazard at time. The linear combination, $X^{T}\beta$ , can be interpreted as an uncalibrated version of DNAm GrimAge. To facilitate an intuitive interpretation as a physiological age estimator, we linearly transformed it so that the resulting estimate would be in units of years. Toward this end, we imposed the following requirement:

the mean and variance of the resulting value, DNAm GrimAge, should be the same as the mean and variance of the age variable in the FHS training data (exam 7).

This resulted in the following transformation

DNAm GrimAge =$\;-50.28483+8.3268*X^{T}\beta .$

### Observed GrimAge

While our DNAm GrimAge was defined with respect to DNAm based surrogate biomarkers, our *observed* Grim Age estimators is *not* based on DNA methylation levels. Rather, it is based on observed plasma protein levels, self-report pack-years, age, and gender. Observed GrimAge was built by fitted a Cox regression model using the observed variables in the same FHS training data that were used for building the DNAm GrimAge estimator. We computed a corresponding measure of age acceleration, called *observed* AgeAccelGrim, by adjusting observed GrimAge for chronological age (defined as raw residual resulting from regressing observed GrimAge on chronological age).

### Statistical models used in validation analysis

Validation analysis was performed on 7,735 observations across 6,395 individuals (Table 2 and Supplementary Note 1) coming from five datasets: the FHS test dataset (N=625), WHI BA23 (N=2107), WHI EMPC (N=1972), Jackson Heart Study (JHS, N=1747), and InChianti study (N=924 from 1 to 2 longitudinal measures on 484 individuals, Table 2 and Supplementary Note 1). Our validation analysis involved i) Cox regression for time to death, for time-to-CHD, and for time to coronary heart failure, ii) linear regression for our DNAm based measures (independent variable) associated with and number of age-related conditions (dependent variable) and physical function score, respectively, iii) linear regression for age at menopause (independent variable) associated with our DNAm measure, iv) logistic regression analysis for estimating the odds ratios of our DNAm based measure associated with any cancer, hypertension, type 2 diabetes, emphysema, and disease free status. The variable of “number of age-related conditions” includes arthritis, cataract, cancer, CHD, CHF, emphysema, glaucoma, lipid condition, osteoporosis, type 2 diabetes, etc. (see Supplementary Note 1). In our validation analysis, we used AgeAccelGrim (the age-adjusted measure of DNAm GrimAge), and used the scaled measures of seven DNAm surrogates for plasma proteins based on the mean and standard deviation (SD) of the FHS training dataset such that the effect size was approximately corresponding to one SD. All the models were adjusted for age, and adjusted for batch effect as needed. To avoid the bias due to familial correlations from pedigrees in the FHS cohort or the intra subject correlations from the repeated measures, we accounted for the correlations accordingly in all the analyses in the following. In Cox regression analysis, we used robust standard errors, the Huber sandwich estimator, implemented in R *coxph* function. We used linear mixed models with a random intercept term, implemented in *lme* R function. We used generalized estimation equation models (GEE), implemented in R *gee* function, for our logistic regression models. Additional covariates related to demographic characteristics, psychosocial behaviors and clinical covariates were adjusted in multivariate Cox models analysis. The additional covariates includes BMI (category), education attainment (category), alcohol consumption (gram/day), self report smoking pack-years, three medical covariates: status of cancer, hypertension and type 2 diabetes at baseline. The categories associated with BMI ranges are a) 18.5 -25 (normal), b) 25 to 30 (over), and c) >30 (obese). The categories associated with education attainment are a) less than high school, b) high school degree, c) some college, and d) college degree and above. Both smoking pack-years and education variables were not available in the JHS cohort. Smoking category (never, former and current) was used in the analysis using the JHS cohort.

### Meta analysis

We used fixed effect models weighted by inverse variance to combine the results across validation study sets into a single estimate by using the *metafor* R function in most situations. We also used Stouffer’s meta analysis method (weighted by the square root of the sample size) in specific situations where the harmonization of covariates across cohorts was challenging, e.g. when evaluating the number of age-related conditions, disease free status and physical function scores (Fig. 5).

### Heritability analysis

In general, epigenetic measures of age acceleration are highly heritable [52,57,58]. To evaluate whether AgeAccelGrim is heritable as well, we estimated the narrow sense heritability $h^{2}$ using the polygenic models defined in SOLAR [59] and its R interface solarius [60]. Heritability is defined as the total proportion of phenotypic variance attributable to genetic variation in the polygenic model. All traits were adjusted for age and gender. The robust polygenic model (with the option of a t-distribution) was used to estimate heritability of AgeAccelGrim and DNAm based proteins. The heritability estimate correspondents to the variance component associated with the kinship coefficient. If the corresponding P value is significant (P<0.05), the underlying trait is deemed to be heritable.

### Two stage estimate of mortality versus a single stage estimate of mortality

To develop our *single stage* mortality estimator, DNAm Mortality, we used elastic net Cox regression to regress time-to-death on the CpG markers, chronological age and sex in the FHS training data. We used the same options in the training process (i.e., 10-fold cross validation for choosing the lambda tuning parameter). The resulting mortality risk estimator, (uncalibrated) DNA Mortality, is a linear combination of 59 CpGs and chronological age. Next we used the same age calibration method that we previously used for DNAm GrimAge to arrive at a mortality risk estimator in units of years, DNAm Mortality. We also evaluated the two mortality risk estimators by Zhang (on the basis of 10 CpGs) [21]. The first risk estimator from Zhang is a composite score based on 10 CpGs with weights determined by a Cox regression with lasso penalty. Of the 10 CpGs, cg06126421 and cg23665802 were absent in the JHS cohort and had to be imputed (by the respective median values in the FHS training data).

To provide an unbiased comparison with our mortality predictors, we applied our age calibration method to the Zhang estimator as well, resulting in the mortality predictor "DNAmZhang". The second Zhang estimator, referred as DNAmZhangScore, was defined as the total sum of scores of the 10 CpGs with aberrant methylation [21]. The resulting risk score ranges from 0 to 10.

### AgeAccelGrim versus blood cell composition

The imputed blood cell abundance measures were related to DNAm Grim Age models using the validation study sets: FHS test, WHI BA23, JHS, and InChianti, involving n=6,003 individuals. The following imputed blood cell counts were analyzed: B cell, naïve CD4+ T, CD4+ T, naïve CD8+ T, CD8+ T, exhausted cytotoxic CD8+ T cells (defined as CD8 positive CD28 negative CD45R negative), plasma blasts, natural killer cells, monocytes, and granulocytes. The abundance of naive T cells, exhausted T cells, and plasma blasts were based on the Horvath method [61]. The remaining cell types were imputed using the Houseman method [31]. More details were described in Supplementary Methods. To avoid confounding by age, we used AgeAccelGrim and adjusted all DNAm based surrogate biomarkers by chronological age (by forming residuals). The correlation results were combined across studies via the same fixed effect models.

### Cox models that include blood cell counts

We also fit multivariate Cox regression models that adjusted for imputed blood cell counts in addition to chronological age, batch, and pedigree structure, for predicting time-to-death and time-to-CHD. The blood cell counts were imputed based on DNA methylation levels (as detailed above). To avoid multi-collinearities between blood cell counts, we only included the following 7 blood cell counts into the multivariate model: naïve CD8+T, exhausted cytotoxic CD8+ T cells, plasma blasts, CD4+T, natural killer cells, monocytes and granulocytes.

### GREAT analysis

We applied the GREAT analysis software tool [32] to sets of CpGs (e.g. 1030 CpGs underlying DNAm GrimAge).CpGs in non-coding regions typically lack annotation with respect to biological functions. GREAT assigns biological meaning to a set of non-coding genomic regions (implicated by the CpGs) by analyzing the annotations of the nearby genes. Toward this end, the GREAT software performs both a binomial test (over genomic regions) and a hypergeometric test over genes when using a whole genome background. We performed the enrichment based on default settings (Proximal: 5.0 kb upstream, 1.0 kb downstream, plus Distal: up to 1,000 kb) for gene sets associated with GO terms, MSigDB, PANTHER and KEGG pathway. To avoid large numbers of multiple comparisons, we restricted the analysis to the gene sets with between 10 and 3,000 genes. We report nominal P values and two adjustments for multiple comparisons: Bonferroni correction and the Benjamini-Hochberg false discovery rate.

### Lifestyle factors including diet and education

We performed a robust correlation analysis (biweight midcorrelation, bicor [62]) between our novel biomarkers (AgeAccelGrim and its eight age-adjusted components) and 38 variables from the WHI including 12 self-reported dietary variables, behavioral variables, 9 dietary biomarkers, 12 variables related to metabolic related traits and central adiposity, and 5 life style factors. We combined the postmenopausal women from the WHI BA23 and WHI EMPC (roughly n= 4000 women). This cross sectional, robust correlation analysis was conducted in all groups combined and in three separate ethnic groups (Hispanic ancestry, European ancestry, African Ancestry). Ancestry information was verified using ancestry informative SNP markers. Blood biomarkers were measured from fasting plasma collected at baseline. Food groups and nutrients are inclusive, including all types and all preparation methods, e.g. folic acid includes synthetic and natural, dairy includes cheese and all types of milk. The individual variables are explained in [33].

### Computed tomography data from the Framingham Heart Study

The computed tomography (CT) in liver, spleen, paraspinal muscle, subcutaneous adipose tissue (SAT), and visceral adipose tissue (VAT) were performed in n=2,803 individuals from the FHS Offspring, Third Generation and Omni 2 Cohort participants between September 2008 and December 2011 [37,38]. Of those, 1,177 Offspring Cohort participants were included in our FHS study. The age at CT scan was in general slightly older than the age at blood draw for the DNA methylation profile (mean age difference= 3.7 years, ranging from 1.2 to 6.1 years).

Organ density measures, more precisely CT attenuation coefficients, reflect how easily a target can be penetrated by an X-ray. The Hounsfield unit (HU) scale is a linear transformation of the original linear attenuation coefficient measurement into one in which the radiodensity of distilled water is defined as zero Hounsfield units (HU). Radiation attenuation in liver, spleen, or muscle is inversely related to respective measures of fat content.

The CT measures from three areas of the liver, two areas of the spleen and two areas of the paraspinal muscle were averaged to determine the average Hounsfield units in liver, spleen and muscle, respectively. The CT-scan measures of visceral and subcutaneous adipose tissue are described in [38].

In our analysis, we first performed marginal robust correlation analysis (biweight midcorrelation, bicor coefficient) [62] to study the association between the CT-scan derived measures and DNAm based biomarkers. As gender affects adipose associated parameters, we performed the analysis in males and females, separately. Next we combined the results across the two genders using fixed effects meta analysis. To adjust for potential confounders, we also performed three types of multivariate linear mixed effects models that included gender, BMI as fixed effects and pedigree structure as random effect. In Model I, we regressed a DNAm based biomarker (e.g. AgeAccelGrim) on CT derived covariates: liver density, spleen density, and paraspinal muscle density. In Model II, we regressed the DNAm based biomarker (dependent variable) on volumetric measures of adipose tissue (both SAT and VAT volume). We omitted measures of adipose tissue density from the analysis since a) they were not significant after adjusting for SAT/VAT volumes, and b) we wanted to protect the model fit from issues of multi-collinearity.

In Model III, we used all CT measures as covariates (i.e. liver, spleen and muscle density, SAT volume, and VAT volume). We used the BMI measure assessed at exam 9 in the FHS, i.e. the closest exam following the CT-scan exam.

## Supplementary Material

Supplementary Table of Content

Supplementary Note 1

Supplementary Note 2

Supplementary Methods

Supplementary Figures

Supplementary Tables

Supplementary References

Supplementary Data 1

Supplementary Data 2

Supplementary Data 3
