# Supplementary material for: DNA methylation GrimAge strongly predicts lifespan and healthspan
Source: Aging (Albany NY). 2019 Jan 21;11(2):303–27. doi: 10.18632/aging.101684 (PMC6366976; doi:10.18632/aging.101684)
Supplement: Supplementary Table of Content [file aging-11-101684-s001.docx]

**SUPPLEMENTARY MATERIAL**

for the article entitled "DNA methylation GrimAge strongly predicts lifespan and healthspan"

AT Lu et al.

Table of Contents

[Supplementary Note 1. Description of datasets](#_Toc530441030)

[Framingham Heart Study Cohort](#_Toc530441031)

[Women's Health Initiative](#_Toc530441032)

[Jackson Heart Study](#_Toc530441033)

[Invecchiare in Chianti, aging in the Chianti area (InChianti)](#_Toc530441034)

[Supplementary Note 2. DNAm based surrogates for plasma proteins](#_Toc530441035)

[Supplementary Methods. Estimation of blood cell counts based on DNAm levels](#_Toc530441036)

[Supplementary Figure 1. Leptin levels versus sex and age in the FHS](#_Toc530441037)

[Supplementary Figure 2. DNAm GrimAge versus chronological age in different cohorts](#_Toc530441038)

[Supplementary Figure 3. Meta analysis forest plots for predicting time-to-death in never-smokers.](#_Toc530441039)

[Supplementary Figure 4. Meta analysis forest plots for predicting time-to-death in smokers.](#_Toc530441040)

[Supplementary Figure 5. Meta analysis for predicting time-to-congestive heart failure](#_Toc530441041)

[Supplementary Figure 6. Meta-analysis of hypertension status](#_Toc530441042)

[Supplementary Figure 7. Meta-analysis of type 2 diabetes status](#_Toc530441043)

[Supplementary Figure 8. Meta-analysis of physical functioning level](#_Toc530441044)

[Supplementary Figure 9. Meta-analysis of age-at-menopause](#_Toc530441045)

[Supplementary Figure 10. Meta-analysis of disease-free status](#_Toc530441046)

[Supplementary Figure 11. Pairwise correlations between measures of epigenetic age acceleration in the FHS test data](#_Toc530441047)

[Supplementary Figure 12. Comparing measures epigenetic age acceleration with respect to predicting time-to-death](#_Toc530441048)

[Supplementary Figure 13. Comparing measures of epigenetic age acceleration with respect to predicting time-to-death among never-smokers](#_Toc530441049)

[Supplementary Figure 14. Comparing measures of epigenetic age acceleration with respect to predicting time-to-death among smokers](#_Toc530441050)

[Supplementary Figure 15. Kaplan Meier plots of individuals who age slowly/quickly according to different measures of epigenetic age acceleration](#_Toc530441051)

[Supplementary Figure 16. Comparing measures of epigenetic age acceleration wrt. comorbidity count](#_Toc530441052)

[Supplementary Figure 17. Comparing measures of epigenetic age acceleration wrt. predicting time-to-coronary heart disease](#_Toc530441053)

[Supplementary Figure 18. Comparing measures of epigenetic age acceleration wrt. predicting time-to-coronary heart disease among never-smokers.](#_Toc530441054)

[Supplementary Figure 19. Comparing measures of epigenetic age acceleration wrt. predicting time-to-coronary heart disease among smokers](#_Toc530441055)

[Supplementary Figure 20. Comparing measures of epigenetic age acceleration wrt. predicting time-to-cancer .](#_Toc530441056)

[Supplementary Figure 21. Comparing measures of epigenetic age acceleration wrt. age-at-menopause](#_Toc530441057)

[Supplementary Figure 22. Sensitivity analysis of the meta analysis for age-at-menopause](#_Toc530441058)

[Supplementary Figure 23. Multivariate Cox regression analysis of time-to-death for epigenetic measures of age acceleration](#_Toc530441059)

[Supplementary Figure 24. Multivariate Cox regression analysis of time-to-CHD for epigenetic measures of age acceleration](#_Toc530441060)

[Supplementary Figure 25. Comparing DNAm GrimAge with single stage mortality predictors wrt. time-to-death](#_Toc530441061)

[Supplementary Figure 26. Comparing DNAm GrimAge with single stage mortality predictors wrt. time-to-coronary heart disease](#_Toc530441062)

[Supplementary Figure 27. Measures of blood cell composition versus DNAm based biomarkers.](#_Toc530441063)

[Supplementary Figure 28. Meta analysis forest plots for predicting time-to-death adjusted for blood cell composition.](#_Toc530441064)

[Supplementary Figure 29. Meta analysis forest plots for predicting time-to-coronary heart disease adjusted for blood cell composition.](#_Toc530441065)

[Supplementary Figure 30. Marginal correlation analysis of lifestyle factors, biomarkers, and age-adjusted DNAm biomarkers in the WHI](#_Toc530441066)

[Supplementary Figure 31. Marginal correlation analysis of clinical biomarkers versus age-adjusted DNAm biomarkers in the FHS](#_Toc530441067)

[Supplementary Figure 32. Correlation analysis of chronological age versus CT-scan fatty liver and adipose tissue density in the FHS](#_Toc530441068)

[Supplementary Figure 33. Marginal correlation analysis of CT-scan fatty liver associated and adipose tissue density with age-adjusted DNAm biomarkers in the FHS](#_Toc530441069)

[Supplementary Table 1. Characteristics of the Framingham Heart Study](#_Toc530441070)

[Supplementary Table 2. Multivariate regression model for estimating DNA GrimAge](#_Toc530441071)

[Supplementary Table 3. Comparing self-reported versus DNAm based estimates of smoking pack years wrt. predicting time-to-death.](#_Toc530441072)

[Supplementary Table 4. Comparing ImmunoAssay versus DNAm based estimates of plasma proteins wrt. predicting time-to-death.](#_Toc530441073)

[Supplementary Table 5. Mortality prediction in the FHS based on DNAm- and observed versions of AgeAccelGrim](#_Toc530441074)

[Supplementary Table 6. Heritability analysis of observed and DNAm based variables](#_Toc530441075)

[Supplementary Table 7. Stratification analysis of time-to-death among epigenetic measures of age acceleration](#_Toc530441076)

[Supplementary Table 8. Stratification analysis of time-to-CHD among epigenetic measures of age acceleration](#_Toc530441077)

[Supplementary Table 9. Distributions of epigenetic age acceleration measures](#_Toc530441078)

[Supplementary Table 10. Correlation analyses between DNAm based biomarkers and leukocyte telomere length](#_Toc530441079)

[Supplementary Table 11. Impact of OMEGA3 on epigenetic age acceleration measures](#_Toc530441080)

[Supplementary Table 12. Multivariate regression analysis of AgeAccelGrim on CT-scan derived fatty liver and adipose tissue density in FHS](#_Toc530441081)

[Supplementary Table 13. Multivariate regression analysis of age-adjusted DNA methylation based plasminogen inhibitor 1 (DNAm PAI-1) on CT-scan derived fatty liver and adipose tissue density in FHS](#_Toc530441082)

[Supplementary References](#_Toc530441083)
