# Supplementary material for: DNA methylation GrimAge strongly predicts lifespan and healthspan
Source: Aging (Albany NY). 2019 Jan 21;11(2):303–27. doi: 10.18632/aging.101684 (PMC6366976; doi:10.18632/aging.101684)
Supplement: Supplementary Note 1 [file aging-11-101684-s002.docx]

# Supplementary Note 1. Description of datasets

Our study involved four cohorts: the Framingham Heart Study (FHS) offspring cohort, the Women's Health Initiative (WHI), Jackson Heart Study (JHS), and Invecchiare in Chianti, aging in the Chianti area (InChianti). We established our DNAm GrimAge model using the individuals from the FHS cohort. Of the FHS cohort, 1731 individuals were used for the training process in establishing the DNAm GrimAge model and 625 individuals were used for the test process at stage 1. Validation analysis was performed on 7,735 observations across 6,395 individuals coming from five independent datasets: the FHS test dataset (N=625), WHI BA23 (N=2107), WHI EMPC (N=1972), JHS (N=1747), and InChianti study (N=924 from 1 to 2 longitudinal measures on 484 individuals). Below we describe each study cohort/datasets in more detail.

###

### Framingham Heart Study Cohort

The FHS cohort [1] is a large-scale longitudinal study started in 1948, initially investigating the common factors of characteristics that contribute to cardiovascular disease (CVD), https://www.framinghamheartstudy. org/index.php. The study at first enrolled participants living in the town of Framingham, Massachusetts, who were free of overt symptoms of CVD, heart attack or stroke at enrollment. In 1971, the study started FHS Offspring Cohort to enroll a second generation of the original participants’ adult children and their spouses (n= 5124) for conducting similar examinations[2]. Participants from the FHS Offspring Cohort were eligible for our study if they attended both the seventh and eighth examination cycles and consented to having their molecular data used for study. We used the 2,356 participants from the group of Health/Medical/Biomedical (IRB, MDS) consent and available for both Immunoassay array DNA methylation array data. The FHS data are available in dbGaP (accession number: phs000363.v16.p10 and phs000724.v2.p9).

We computed the total of number age-related conditions based on dyslipidemia, hypertension, cardiovascular disease (including coronary heart disease [CHD] or congestive heart failure [CHF]), type 2 diabetes, cancer and arthritis. Time to CHD or time to CHF was truncated at zero if it occurred before exam 8. Deaths among the FHS participants that occurred prior to January 1, 2013 were ascertained using multiple strategies, including routine contact with participants for health history updates, surveillance at the local hospital and in obituaries of the local newspaper, and queries to the National Death Index. Death certificates, hospital and nursing home records prior to death, and autopsy reports were requested. When cause of death was undeterminable, the next of kin were interviewed. The date and cause of death were reviewed by an endpoint panel of 3 investigators.

***DNA methylation quantification***

Peripheral blood samples were collected at the 8^th^ examination. Genomic DNA was extracted from buffy coat using the Gentra Puregene DNA extraction kit (Qiagen) and bisulfite converted using EZ DNA Methylation kit (Zymo Research Corporation). DNA methylation quantification was conducted in two laboratory batches using the Illumina Infinium HumanMethylation450 array (Illumina). Methylation beta values were generated using the Bioconductor *minfi* package with Noob background correction [3].

### Women's Health Initiative

The WHI is a national study that enrolled postmenopausal women aged 50-79 years into the clinical trials (CT) or observational study (OS) cohorts between 1993 and 1998[4, 5]. We included 4,079 WHI participants with available phenotype and DNA methylation array data: 2,107 women from “*Broad Agency Award 23*” (WHI BA23) and 1,972 women from “*Epigenetic Mechanisms of PM-Mediated CVD Risk*” (WHI EMPC). WHI BA23 focuses on identifying miRNA and genomic biomarkers of coronary heart disease (CHD), integrating the biomarkers into diagnostic and prognostic predictors of CHD and other related phenotypes, and other objectives can be found in <https://www.whi.org/researchers/data/WHIStudies/StudySites/BA23/Pages/>home.

aspx. WHI EMPC is a study of epigenetic mechanisms underlying associations between ambient particulate matter (PM) air pollution and cardiovascular disease [6]. WHI EMPC and BA23 span three WHI sub-cohorts including GARNET, WHIMS and SHARe. 936 EMPC participants were not in any of the WHI GWAS (either GARNET, WHIMS, SHARe, MOPMAP, HIPFX, or GECCO). The largest overlap was with SHARE & GARNET. There was almost no overlap with WHIMS & MOPMAP.

The total number of age-related conditions was based on Alzheimer’s disease, amyotrophic lateral sclerosis, arthritis, cancer, cataract, CVD, glaucoma, emphysema, hypertension, and osteoporosis.

***DNA methylation quantification for BA23***

In brief, bisulﬁte conversion using the Zymo EZ DNA Methylation Kit (Zymo Research, Orange, CA, USA) as well as subsequent hybridization of the HumanMethylation450k Bead Chip (Illumina, San Diego, CA), and scanning (iScan, Illumina) were performed according to the manufacturers protocols by applying standard settings. DNA methylation levels (β values) were determined by calculating the ratio of intensities between methylated (signal A) and un-methylated (signal B) sites. Specifically, the β value was calculated from the intensity of the methylated (M corresponding to signal A) and un-methylated (U corresponding to signal B) sites, as the ratio of fluorescent signals β = Max(M,0)/[Max(M,0)+Max(U,0)+100]. Thus, β values range from 0 (completely un-methylated) to 1 (completely methylated).

***DNA methylation quantification for WHI EMPC***

Illumina Infinium HumanMethylation450 BeadChip data from the Northwestern University Genomics Core Facility for WHI EMPC participants sampled in stages 1a, 1b, and 2 were quality controlled, normalized and batch adjusted. Beta-mixture quantile normalization was implemented using BMIQ [7] and empirical Bayes methods of batch adjustment for stage and plate were implemented in ComBat [8].

***Lifestyle factors and dietary assessment in the Women's Health Initiative (WHI)***

WHI participants completed self-administered questionnaires at baseline which provided personal information on a wide range of topics, including sociodemographic information (age, education, race, income), and current health behaviors (recreational physical activity, tobacco and alcohol exposure, and diet). Participants also visited clinics at baseline where certified Clinical Center staff collected blood specimens and measured anthropometrics (weight, height, hip and waist circumferences) and blood pressures (systolic, diastolic). Body mass index and waist to hip ratio were calculated from these measurements.

Dietary intake was assessed at baseline using the WHI Food Frequency Questionnaire [9]. Briefly, participants were asked to report on dietary habits in the past three months, including intake, frequency, and portion sizes of foods or food groups, along with questions concerning topics such as food preparation practices and types of added fats. Nutrient intake levels were then estimated from these responses. For current drinker, we use the threshold of more than one serving equivalent (14g) within the last 28 days.

###

### Jackson Heart Study

The JHS is a large, population-based observational study evaluating the etiology of cardiovascular, renal, and respiratory diseases among African Americans residing in the three counties (Hinds, Madison, and Rankin) that make up the Jackson, Mississippi metropolitan area [10]. The age at enrollment for the unrelated cohort was 35-84 years; the family cohort included related individuals >21 years old. Participants provided extensive medical and social history, had an array of physical and biochemical measurements and diagnostic procedures, and provided genomic DNA during a baseline examination (2000-2004) and two follow-up examinations (2005-2008 and 2009-2012). Annual follow-up interviews and cohort surveillance are ongoing. In our analysis, we used the visits at baseline from 1747 individuals as part of project JHS ancillary study ASN0104, available with both phenotype and DNA methylation array data. Total numbers of age-related conditions were based on hypertension, type 2 diabetes, kidney dysfunction based on ever dialysis, and CVD.

***DNA methylation quantification***

Peripheral blood samples were collected at the baseline. Methylation beta values were generated using the Bioconductor *minfi* package with Noob background correction [3].

### Invecchiare in Chianti, aging in the Chianti area (InChianti)

The InChianti (Invecchiare in Chianti, aging in the Chianti area) cohort is a representative population-based study of older persons enrolling individuals aged 20 years and older from two areas in the Chianti region of Tuscany, Italy, http://inchiantistudy.net/wp/. One major goal of the study is to translate epidemiological research into geriatric clinical tools, ultimately advancing clinical applications in older persons. Of the cohort, 924 observations from 484 individuals with both phenotype information and DNA methylation data were including in our studies. The observations were collected from baseline in 1998 and the third follow-up visit in 2007. All participants provided written informed consent to participate in this study. The study complied with the Declaration of Helsinki. The Italian National Institute of Research and Care on Aging Institutional Review Board approved the study protocol. We computed the total number of age-related conditions based on cancer, hypertension, myocardial infarction, Parkinson’s disease, stroke and type 2 diabetes.

***DNA methylation quantification***

Genomic DNA was extracted from buffy coat samples using an AutoGen Flex and quantified on a Nanodrop1000 spectrophotometer prior to bisulfite conversion. Genomic DNA was bisulfite converted using Zymo EZ-96 DNA Methylation Kit (Zymo Research Corp., Irvine, CA) as per the manufacturer’s protocol. CpG methylation status of 485,577 CpG sites was determined using the Illumina Infinium HumanMethylation450 BeadChip (Illumina Inc., San Diego, CA) as per the manufacturer’s protocol and as previously described [11]. Initial data analysis was performed using GenomeStudio 2011.1 (Model M Version 1.9.0, Illumina Inc.). Threshold call rate for inclusion of samples was 95%. Quality control of sample handling included comparison of clinically reported sex versus sex of the same samples determined by analysis of methylation levels of CpG sites on the X chromosome [11]. Methylation beta values were generated using the Bioconductor *minfi* package with Noob background correction [3].
