# Supplementary material for: DNA methylation GrimAge strongly predicts lifespan and healthspan
Source: Aging (Albany NY). 2019 Jan 21;11(2):303–27. doi: 10.18632/aging.101684 (PMC6366976; doi:10.18632/aging.101684)
Supplement: Supplementary Note 2 [file aging-11-101684-s003.docx]

**Supplementary Note 2. DNAm based surrogates for plasma proteins**

The model of DNAm GrimAge is composed of seven DNAm based plasma proteins, DNAm based pack years, age and gender. Below we briefly describe the seven plasma proteins.

**ADM (adrenomedullin)** is a vasodilator peptide hormone. Plasma ADM, initially isolated from adrenal gland, is increased in individuals with hypertension and heart failure [12]. A recent study showed that ADM was involved in age-related memory loss in mice and aging human brains [13].

**B2M (Beta-2 microglobulin)** is a component of major histocompatibility complex class 1 (MHC I) molecular. Plasma B2M is a clinical biomarker associated with cardiovascular disease, kidney function, inflammation severity[14]. B2M is a pro-aging factor associated with cognitive and regenerative function in aging process and suggests B2M may be targeted therapeutically in old age [15]. A previous study showed that systemic B2M accumulation in aging blood promoted age-related cognitive dysfunction and impairs mouse models [15].

**Cystatin C or cystatin 3** (formerly gamma trace, post-gamma-globulin, or neuroendocrine basic polypeptide) is mainly used as a biomarker of kidney function. Plasma cystatin-C is a clinical relevant biomarker indicating kidney function[16]. Cystatin-C seems plays a role in cardiovascular disease [17] or amyloid deposition associated with Alzheimer’s disease [18].

**GDF-15 (growth differentiation factor 15)** is one of transforming growth factor beta subfamily. GDF-15 has been implicated in aging and age- related disorders. It also plays a role in age-related mitochondria dysfunction [19].

**Leptin** is a hormone predominantly in adipose cells. Leptin plays a role in regulating energy balance by inhibiting hunger and is implicated in Alzheimer’s disease [20].

**Plasminogen activator inhibitor antigen type 1(PAI-1)** is the major inhibitor of tissue-type plasminogen activator and unokinase plasminogen activator. PAI-1, released in response to inflammation process, plays a central role in a number of age-related subclinical (i.e., inflammation, atherosclerosis, insulin resistance) and clinical conditions (i.e., obesity, comorbidities) [21].

**TIMP-1 or TIMP metallopeptidase inhibitor 1** is a tissue inhibitor of metalloproteinases. It is also involves chromatin structures, promoting cell proliferation in a wide range of cell types, and may also have an anti-apoptotic function [22].
