# Supplementary material for: DNA methylation GrimAge strongly predicts lifespan and healthspan
Source: Aging (Albany NY). 2019 Jan 21;11(2):303–27. doi: 10.18632/aging.101684 (PMC6366976; doi:10.18632/aging.101684)

# SUPPLEMENTARY FIGURES

# Supplementary Figure 1. Leptin levels versus sex and age in the FHS.

Panel (A) Observed leptin levels (based on the immunoassay) versus sex (x-axis) in the test data from the FHS. (B) The surrogate DNAm based biomarker, DNAm leptin, versus sex. (C,D) Mortality risk (technically defined as the deviance residual mortality.res) versus DNAm leptin levels (x-axis) in (C) males and (D) females of the FHS test data, respectively. (E, F) Chronological age versus DNAm leptin levels (x-axis) in (E) males and (F) females of the FHS test data, respectively.


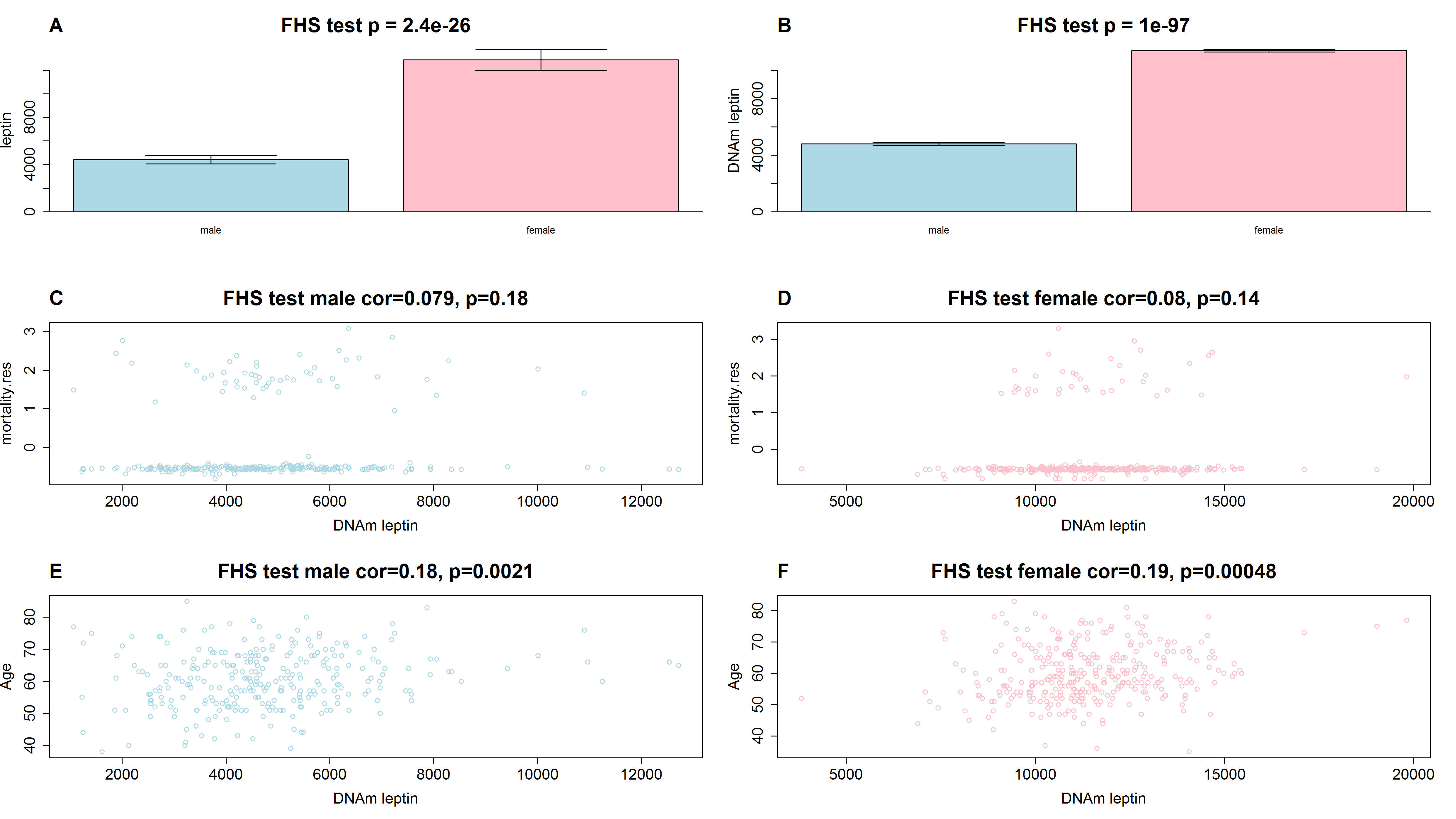


# Supplementary Figure 2. DNAm GrimAge versus chronological age in different cohorts.

Each panel depicts a scatter plot of DNAm GrimAge (x-axis) versus chronological age at the time of the blood draw (x-axis). The title of each panel reports the data set, the Pearson correlation coefficient, and a corresponding correlation test p-value. (A,B) Framingham Heart Study data that were used (A) to develop (train) the DNAm GrimAge estimator, and (B) to test it. Analogous results for (C) the BA23 sub-study of the Women's Health Initiative, (D) WHI EMPC, (E) Jackson Heart Study, (F) InChianti cohort.


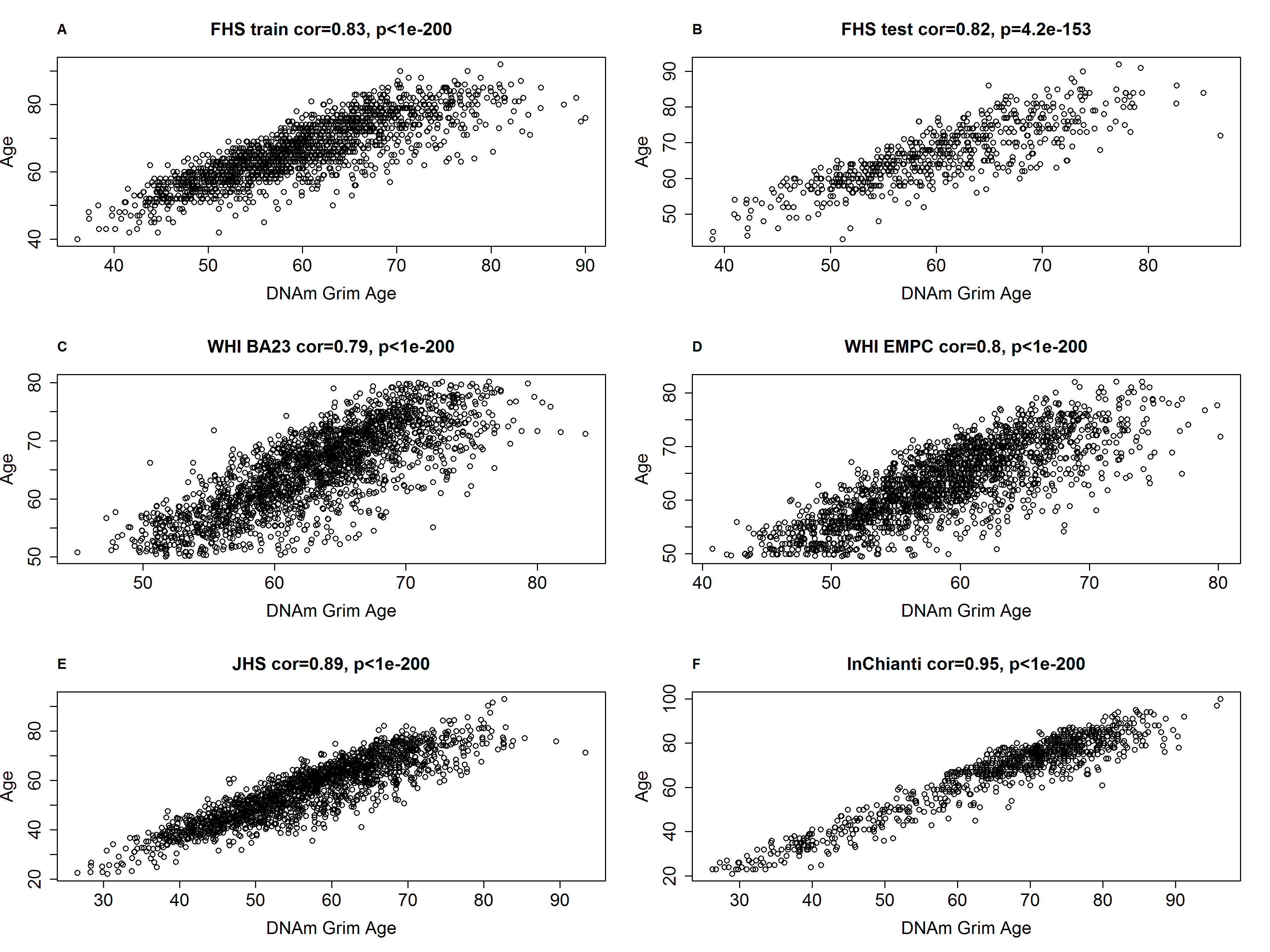


# Supplementary Figure 3. Meta analysis forest plots for predicting time-to-death in never-smokers.

Each panel reports a meta analysis forest plot for combining hazard ratios predicting time-to-death based on a DNAm based biomarker (reported in the figure heading) across different strata formed by racial group within cohort. (A) Results for AgeAccelGrim. Each row reports a hazard ratio (for time-to-death) and a 95% confidence interval resulting from a Cox regression model in each of 5 strata (defined by cohort). Results for (age-adjusted) DNAm based surrogate markers of (B) adrenomedullin (ADM), (C) beta-2 microglobulin (B2M), (D) cystatin C (Cystatin C), (E) growth differentiation factor 15 (GDF-15), (F) leptin, (G) plasminogen activation inhibitor 1 (PAI-1), (H) tissue inhibitor metalloproteinase 1 (TIMP-1) and I) smoking pack-years (PACKYRS). The sub-title of each panel reports the meta analysis p-value and a p-value for a test of heterogeneity Cochran Q test (Het.). (A) Each hazard ratio (HR) corresponds to a one-year increase in AgeAccelGrim. (B-H) Each hazard ratio corresponds to an increase in one-standard deviation. I) Hazard ratios correspond to a 1 year increase in pack-years. A *non-*significant Cochran Q test p-value is desirable because it indicates that the hazard ratios don't differ significantly across the strata. The most significant meta-analysis P value (here AgeAccelGrim) is marked in red.


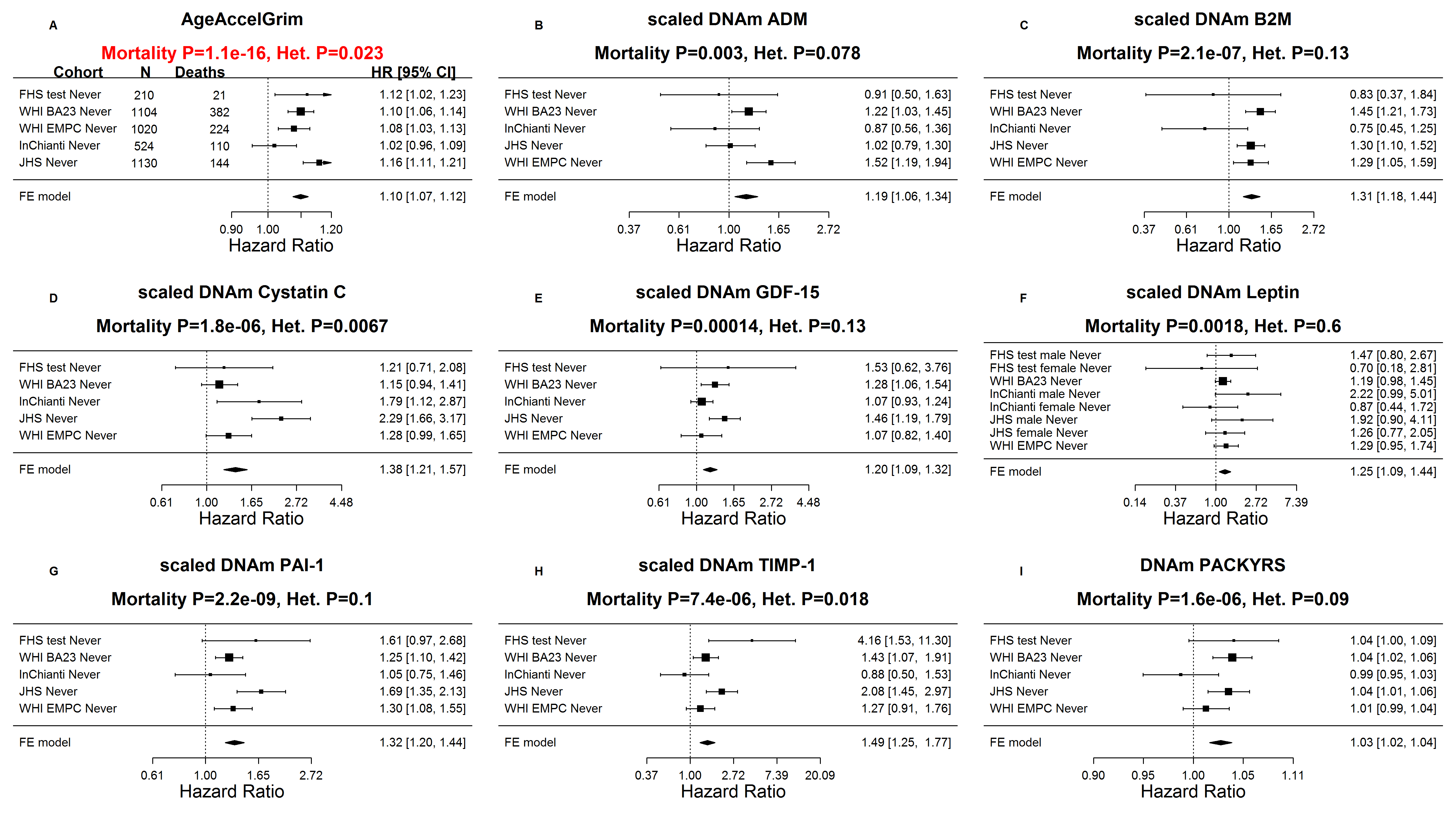


# Supplementary Figure 4. Meta analysis forest plots for predicting time-to-death in smokers.

Here we restrict the analysis to current or former smokers. Each panel reports a meta analysis forest plot for combining hazard ratios predicting time-to-death based on a DNAm based biomarker (reported in the figure heading) across different strata formed by racial group within cohort. (A) Results for AgeAccelGrim. Each row reports a hazard ratio (for time-to-death) and a 95% confidence interval resulting from a Cox regression model in each of 9 strata (defined by cohort and smoking category group). The subgroup “current smokers” of the FHS test cohort (N=38) was removed from analysis as the Cox regression model did not converge due to the small sample size. Results for (age-adjusted) DNAm based surrogate markers of (B) adrenomedullin (ADM), (C) beta-2 microglobulin (B2M), (D) cystatin C (Cystatin C), (E) growth differentiation factor 15 (GDF-15), (F) leptin, (G) plasminogen activation inhibitor 1 (PAI-1), (H) tissue inhibitor metalloproteinase 1 (TIMP-1) and I) smoking pack-years (PACKYRS). The sub-title of each panel reports the meta analysis p-value and a p-value for a test of heterogeneity Cochran Q test (Het.). (A) Each hazard ratio (HR) corresponds to a one-year increase in AgeAccelGrim. B-H) Each hazard ratio corresponds to an increase in one-standard deviation. (I) Hazard ratios correspond to a 1 year increase in pack-years. The most significant meta-analysis P value (here AgeAccelGrim) is marked in red.


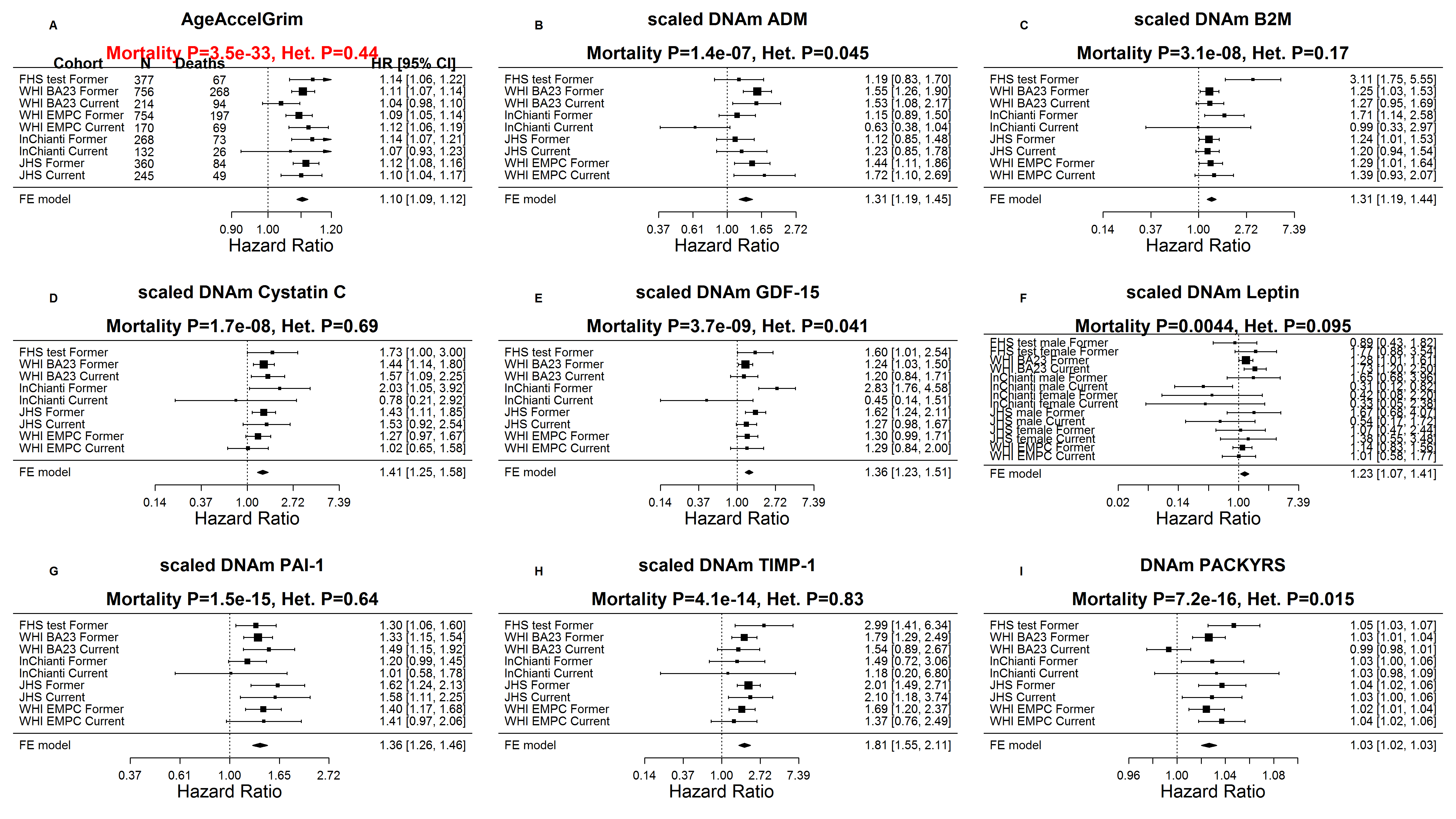


# Supplementary Figure 5. Meta analysis for predicting time-to-congestive heart failure.

Each panel reports a meta analysis forest plot for combining hazard ratios predicting time-to-CHF based on a DNAm based biomarker (reported in the figure heading) across different strata formed by racial groups within cohorts. (A) Results for AgeAccelGrim. Each row reports a hazard ratio (for time-to-CHF) and a 95% confidence interval resulting from a Cox regression model in each of 7strata (defined by cohort and racial groups). Results for (age adjusted) DNAm based surrogate markers of (B) adrenomedullin (ADM), (C) beta-2 microglobulin (B2M), (D) cystatin C (Cystatin C), (E) growth differentiation factor 15 (GDF-15), (F) leptin, G) plasminogen activation inhibitor 1 (PAI-1), (H) tissue inhibitor metalloproteinase 1 (TIMP-1) and (I) smoking pack-years (PACKYRS). The sub-title of each panel reports the meta analysis p-value and a p-value for a test of heterogeneity Cochran Q test (Het.). (A) Each hazard ratio (HR) corresponds to a one-year increase in AgeAccelGrim. (B-H) Each hazard ratio corresponds to an increase in one-standard deviation.( I) Hazard ratios correspond to a one unit increased in DNAm pack-years. The most significant meta-analysis P value is marked in red.


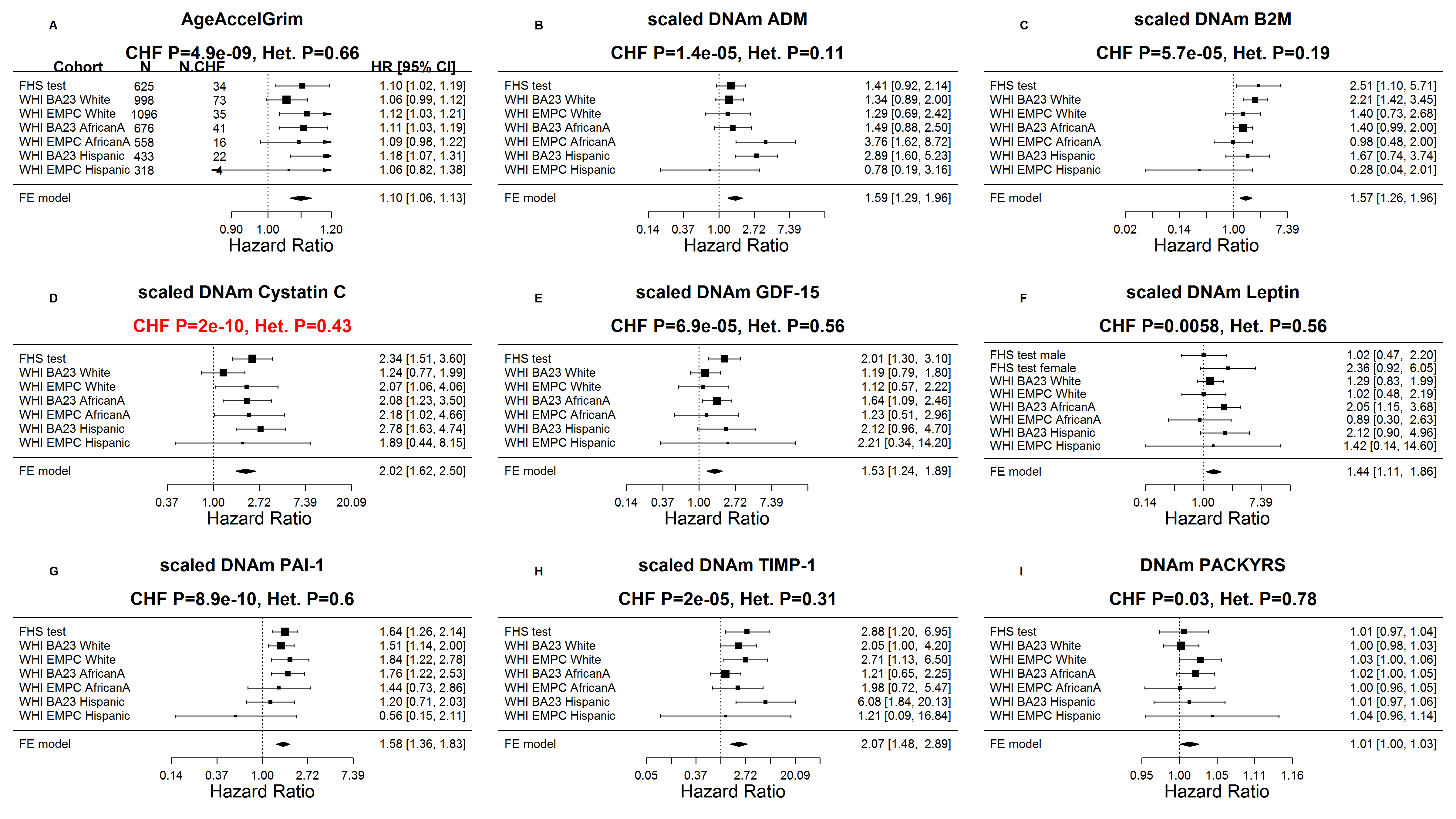


# Supplementary Figure 6. Meta-analysis of hypertension status.

Meta analysis forest plots for the odds ratios between (age-adjusted) DNAm based biomarkers and hypertension status across different strata formed by racial groups within cohorts. Results for (A) AgeAccelGrim, (B-I) underlying DNAm based surrogate biomarkers. All DNAm based biomarkers were adjusted for chronological age at the time of the blood draw. Each panel heading reports the DNAm based biomarker, a meta analysis p value, and the results of a heterogeneity test (Cochran's Q test). A) Each odds ratio (OR) corresponds to a one-year increase in AgeAccelGrim. (B-H) Each OR corresponds to an increase in one-standard deviation. (I) Each OR corresponds to a 1 year increase in smoking pack-years.


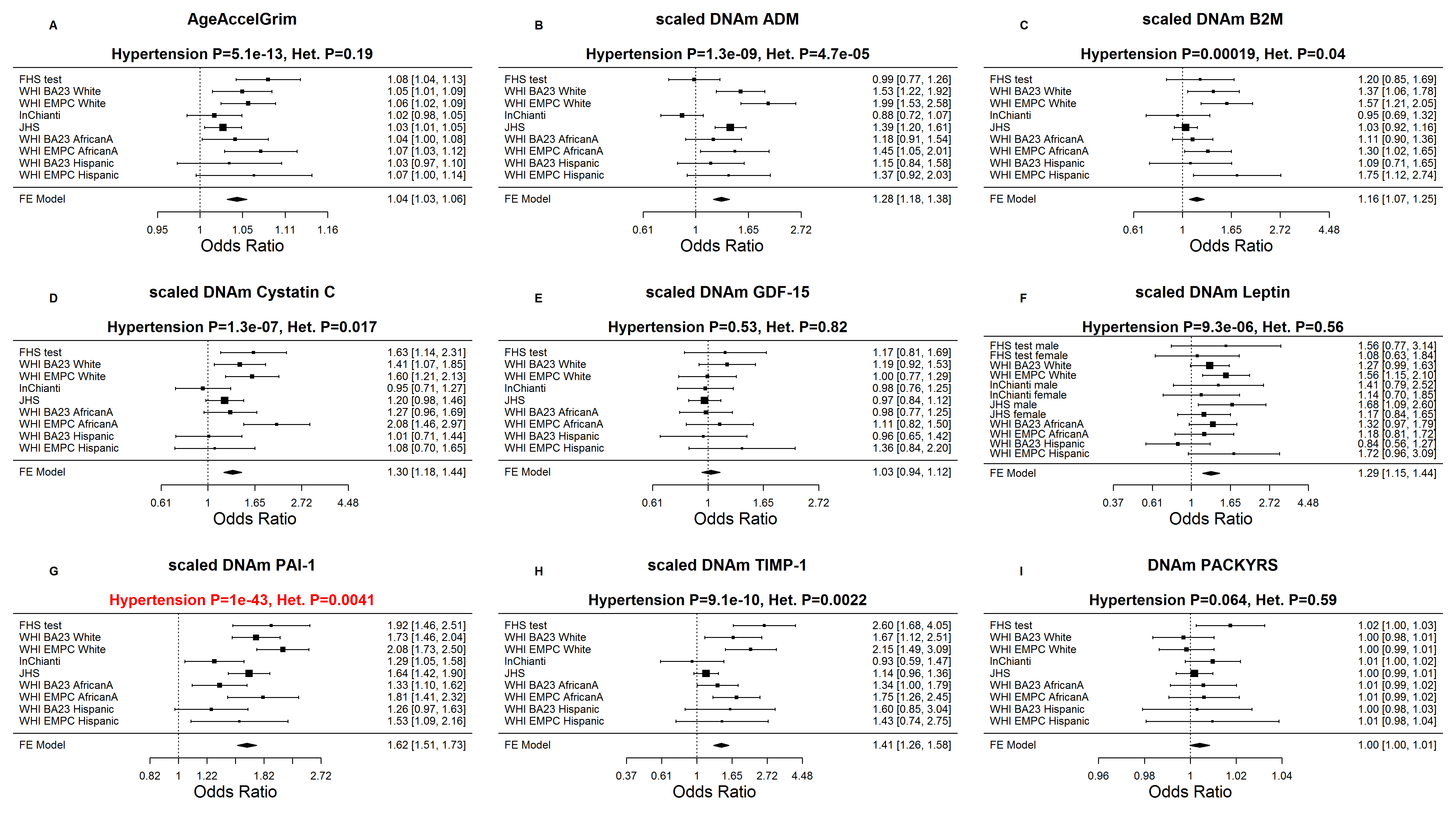


# Supplementary Figure 7. Meta-analysis of type 2 diabetes status.

Meta analysis forest plots for the odds ratios between (age-adjusted) DNAm based biomarkers and type 2 diabetes status across different strata formed by racial groups within cohorts. Results for (A) AgeAccelGrim, (B-I) underlying DNAm based surrogate biomarkers. All DNAm based biomarkers were adjusted for chronological age at the time of the blood draw. Each panel heading reports the DNAm based biomarker, a meta analysis p value, and the results of a heterogeneity test (Cochran's Q test). (A) Each odds ratio (OR) corresponds to a one-year increase in AgeAccelGrim. (B-H) Each OR corresponds to an increase in one-standard deviation. (I) Each OR corresponds to a 1 year increase in smoking pack-years.


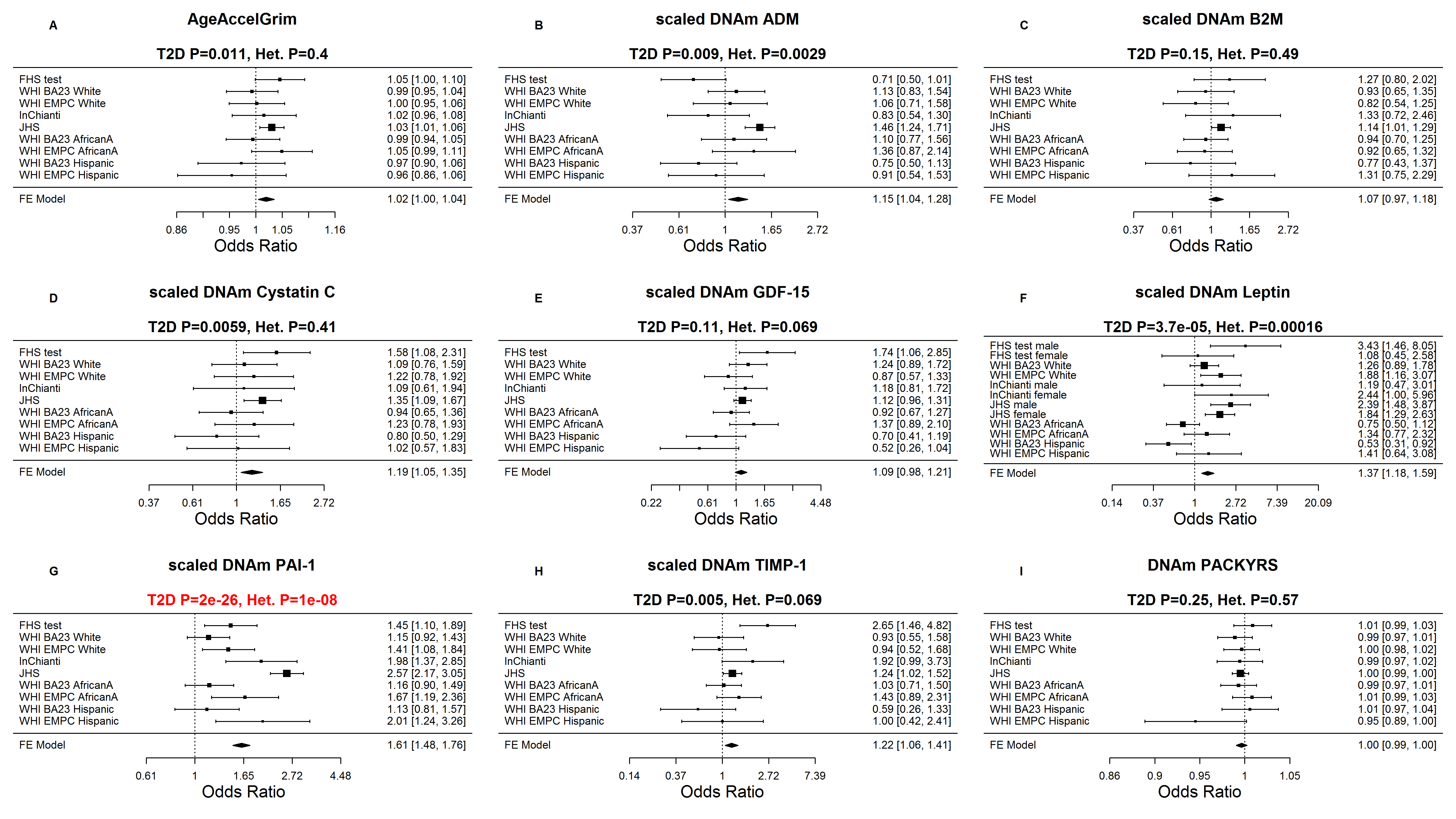


# Supplementary Figure 8. Meta-analysis of physical functioning level.

Meta analysis forest plots (Stouffer's method) for the regression coefficients between (age-adjusted) DNAm based biomarkers (independent variable) and physical functioning levels (dependent variable) across different strata formed by racial groups within cohorts. Results for (A) AgeAccelGrim, (B-I) underlying DNAm based surrogate biomarkers. All DNAm based biomarkers were adjusted for chronological age at the time of the blood draw. Each panel heading reports the DNAm based biomarker, a meta analysis p value, and the results of a heterogeneity test (Cochran's Q test). (A) Each regression slope corresponds to a one-year increase in AgeAccelGrim. (B-H) Each regression slope corresponds to an increase in one-standard deviation. (I) Each regression slope corresponds to a 1 year increase in smoking pack-years.

**
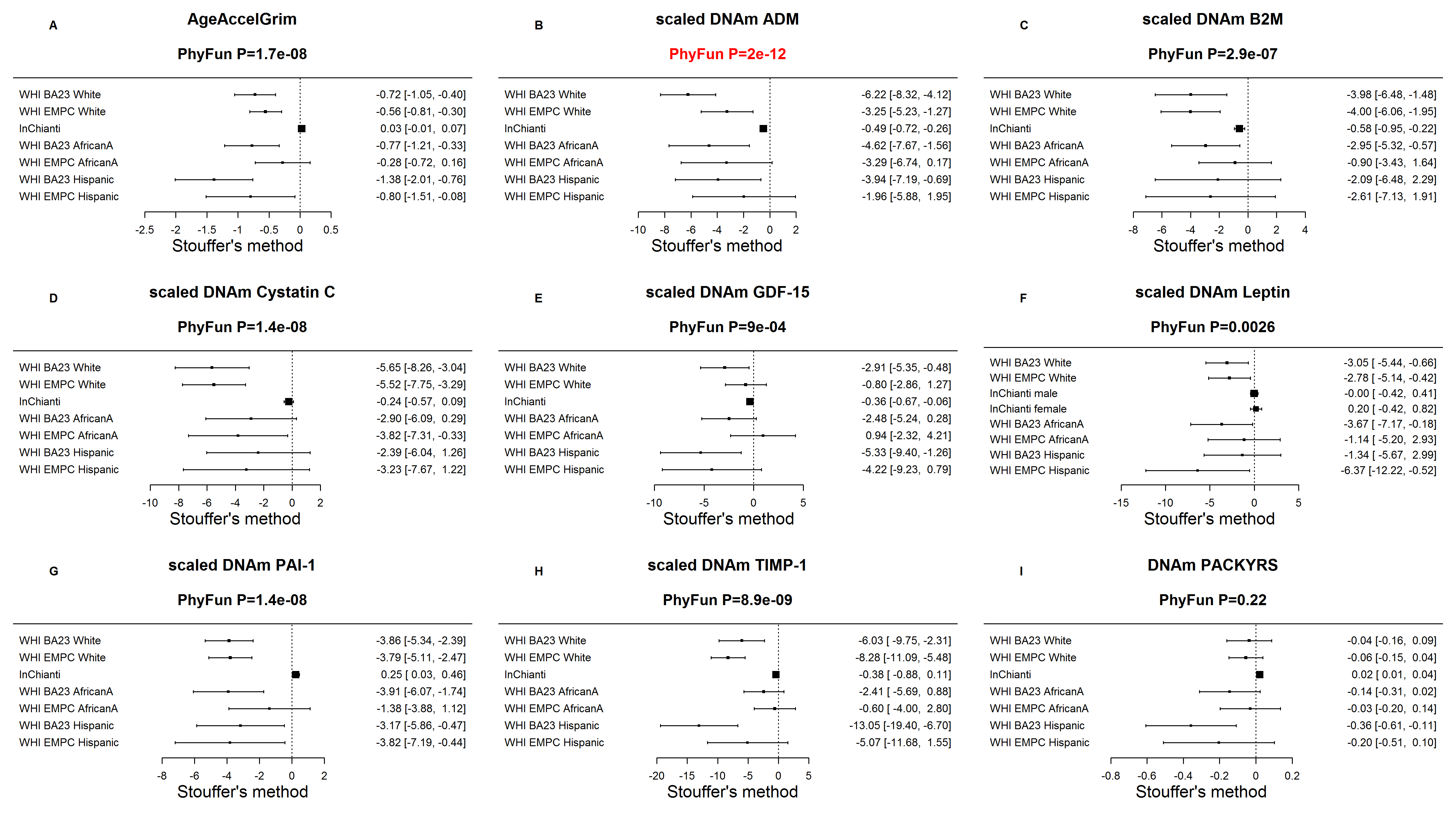
**

# Supplementary Figure 9. Meta-analysis of age-at-menopause.

Each panel reports a meta analysis forest plot for combining regression coefficients (slopes) between age-at-menopause in women (independent variable) and the DNAm based biomarker (dependent variable, as reported in the figure heading) across different strata, witch are formed by racial group within cohort. (A) Meta analysis of the regression slope between AgeAccelGrim and age at menopause. Analogous results for (age adjusted) DNAm based surrogate markers of (B) adrenomedullin (ADM), (C) beta-2 microglobulin (B2M), D) cystatin C (Cystatin C), (E) growth differentiation factor 15 (GDF-15), (F) leptin,(G) plasminogen activation inhibitor 1 (PAI-1), (H) tissue inhibitor metalloproteinase 1 (TIMP-1) and (I) smoking pack-years (PACKYRS). The individual study results were combined using fixed effect meta-analysis (reported in the panel heading). Cochran Q test for heterogeneity across studies (Het.). The effect sizes correspond to one year late of age at menopause.


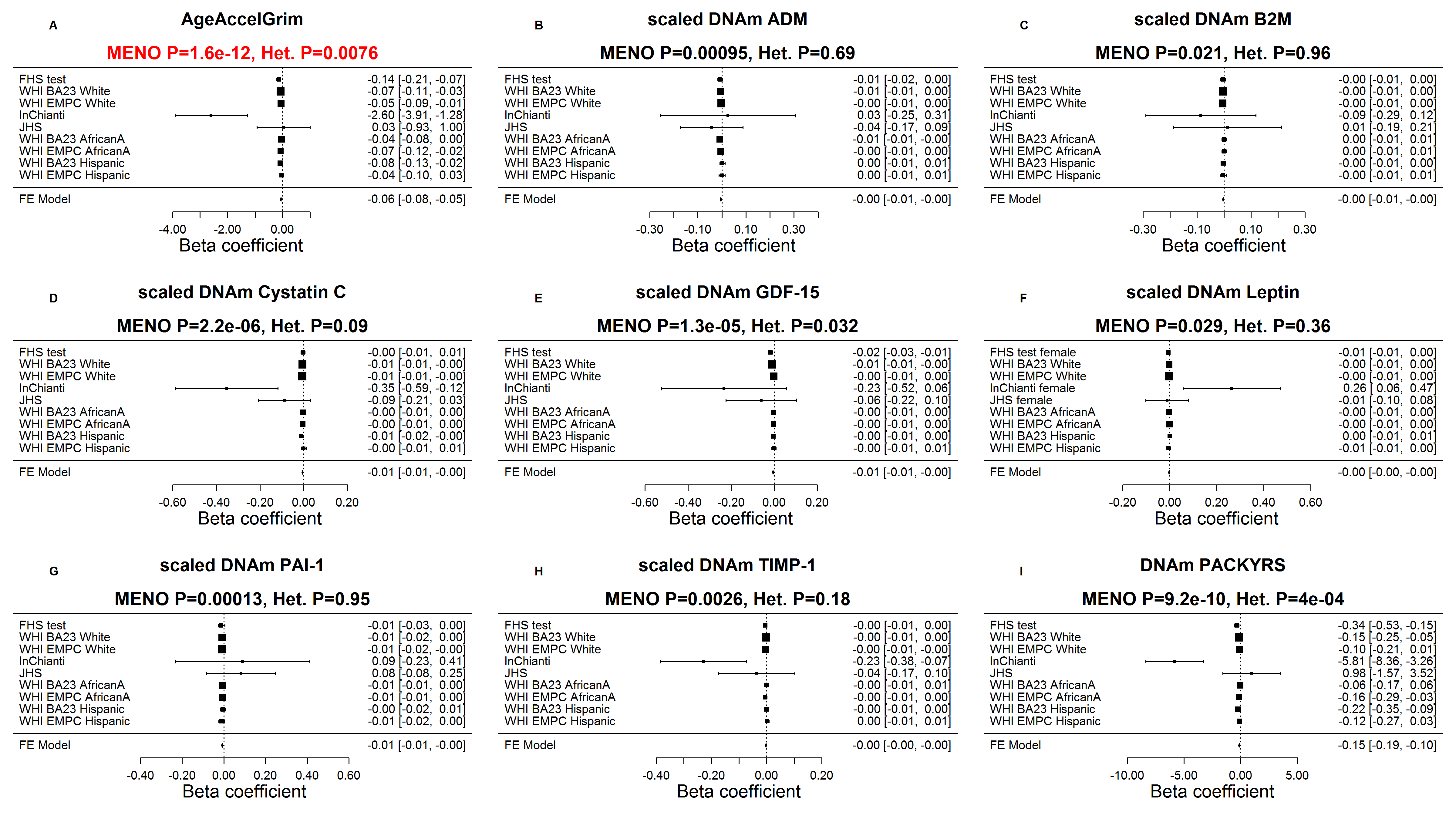


# Supplementary Figure 10. Meta-analysis of disease-free status.

We combined the results across studies based on Stouffer’s method. Each panel reports a meta analysis forest plot for combining logistic regression coefficients between disease-free status and the DNAm based biomarker (reported in the figure heading) across different strata, which are formed by racial group within cohort. (A) Meta analysis of the odds ratios between AgeAccelGrim and disease free status. Analogous results for (age adjusted) DNAm based surrogate markers of (B) adrenomedullin (ADM), (C) beta-2 microglobulin (B2M), (D) cystatin C (Cystatin C), (E) growth differentiation factor 15 (GDF-15), (F) leptin, G) plasminogen activation inhibitor 1 (PAI-1), (H) tissue inhibitor metalloproteinase 1 (TIMP-1) and (I) smoking pack-years (PACKYRS). The individual study results were combined using Stouffer's meta analysis method. Each odds ratio (OR) corresponds to one year of age acceleration in panel A, one pack-year in panel I and one standard deviation in other panels for DNAm proteins. The estimate with the most significant meta P value is marked in red.


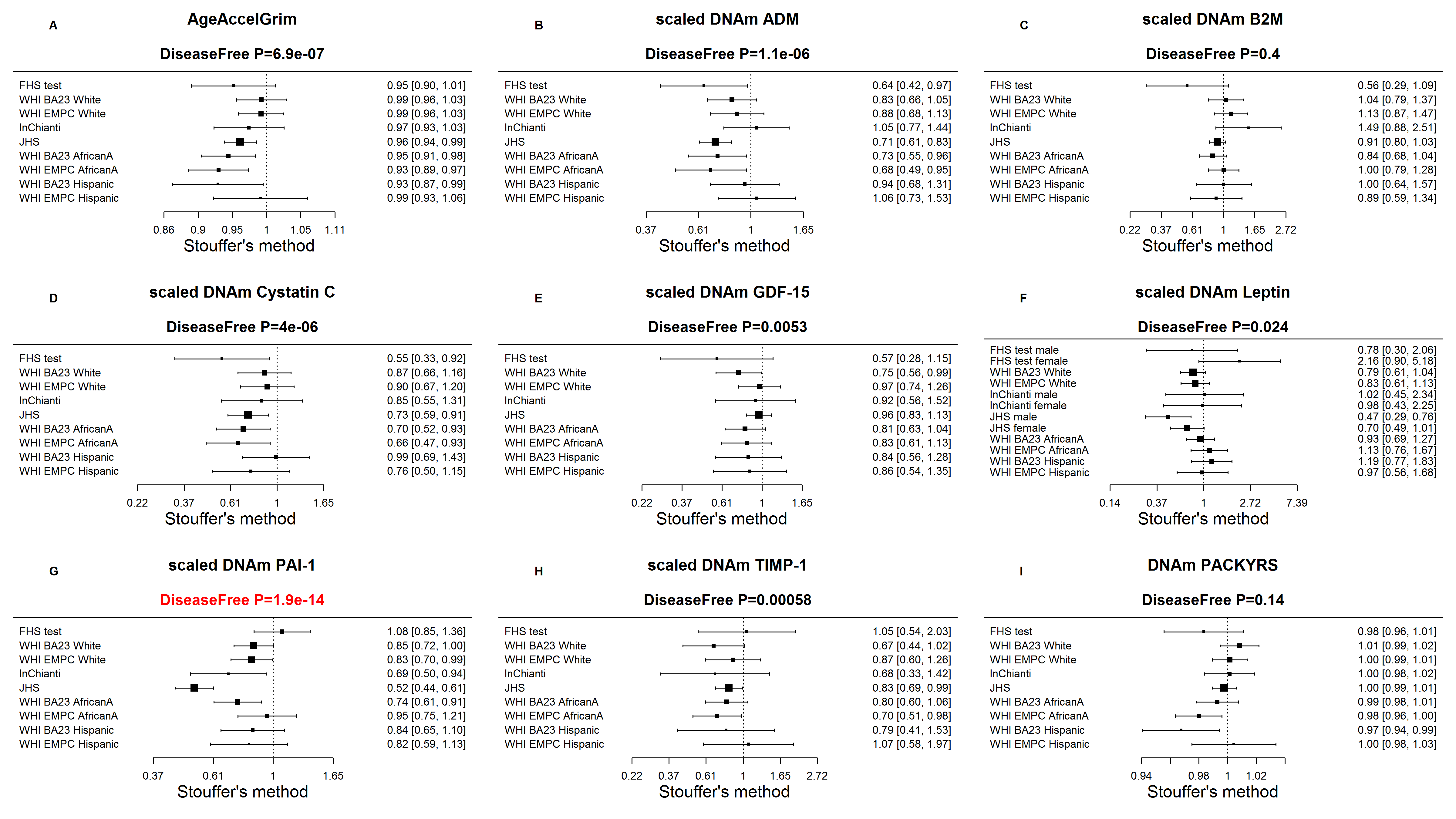


# Supplementary Figure 11. Pairwise correlations between measures of epigenetic age acceleration in the FHS test data.

The heatmap color-codes the pairwise Pearson correlation coefficients between epigenetic measures of age acceleration based on DNAmGrimAge (AgeAccelGrim), the pan-tissue DNAm age developed by Horvath (AgeAccelerationResidual) [24], the blood-based DNAm age developed by Hannum et al. (AgeAccelHannum) [27], and DNAm PhenoAge developed by Levine (AgeAccelPheno) [28]. The analysis is not confounded by chronological age, since the respective measures of age acceleration are not correlated with chronological age.


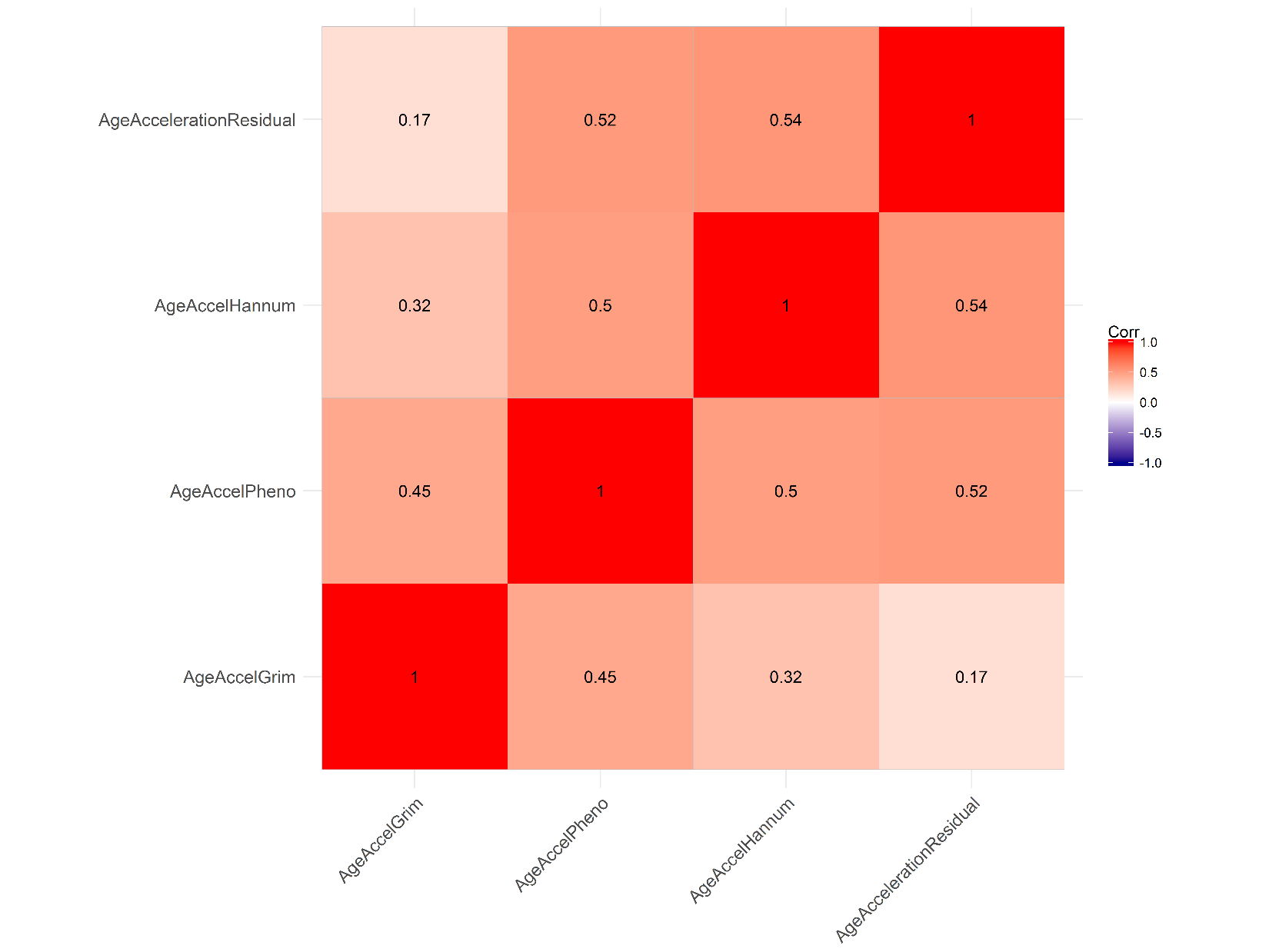


# Supplementary Figure 12. Comparing measures epigenetic age acceleration with respect to predicting time-to-death.

Each panel presents a meta analysis forest plot for combining hazard ratios predicting time-to-death based on an epigenetic measure of age acceleration (reported in the figure heading) across different strata formed by racial group within cohort. Each row reports a hazard ratio (for time-to-death due to all cause mortality) and a 95% confidence interval resulting from a Cox regression model in each of 9 strata (defined by cohort and racial groups). Panels A-D report results for (A) AgeAccelGrim, (B) age-adjusted DNAm PhenoAge (AgeAccelPheno) [28], (C) age-adjusted DNAm age based on Hannum et al. (AgeAccelHannum) [27], and (D) age adjusted DNAm age based on Horvath (AgeAccelResidual) [24]. The meta analysis p-value (colored in red) should be used when it comes to comparing the predictive accuracy of the different measures. It would not be appropriate to compare the hazard ratios directly because the 4 respective measures of age acceleration have different distributions and are scaled differently. Each hazard ratio (HR) corresponds to a 1 year increase in the respective measure of age acceleration. A non-significant p value of the heterogeneity Cochran’s Q test (Het.) is desirable.


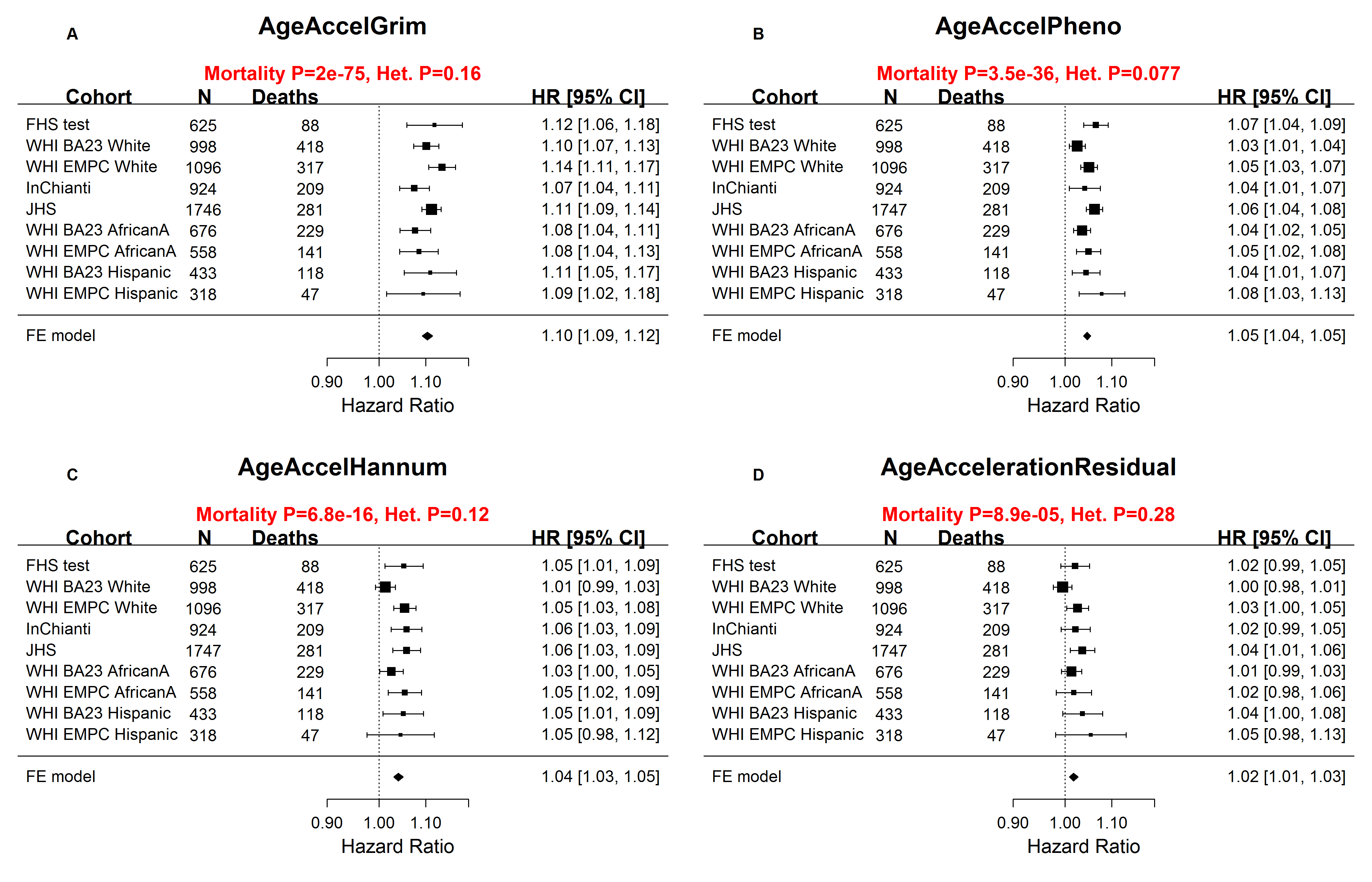


# Supplementary Figure 13. Comparing measures of epigenetic age acceleration with respect to predicting time-to-death among never-smokers.

Here we focus on individuals who never smoked. Each panel presents a meta analysis forest plot for combining hazard ratios predicting time-to-death based on an epigenetic measure of age acceleration (reported in the figure heading) across different strata formed by racial group within cohort. Each row reports a hazard ratio (for time-to-death due to all cause mortality) and a 95% confidence interval resulting from a Cox regression model in each of 9 strata (defined by cohort and racial groups). Panels A-D report results for (A) AgeAccelGrim, (B) age-adjusted DNAm PhenoAge (AgeAccelPheno) [28], (C) age-adjusted DNAm age based on Hannum et al. (AgeAccelHannum) [27], and (D) age adjusted DNAm age based on Horvath (AgeAccelResidual) [24]. The meta analysis p-value (colored in red) should be used when it comes to comparing the predictive accuracy of the different measures. It would not be appropriate to compare the hazard ratios directly because the 4 respective measures of age acceleration have different distributions and are scaled differently. Each hazard ratio (HR) corresponds to a 1 year increase in the respective measure of age acceleration. A non-significant p value of the heterogeneity Cochoran’s Q test (Het.) is desirable.


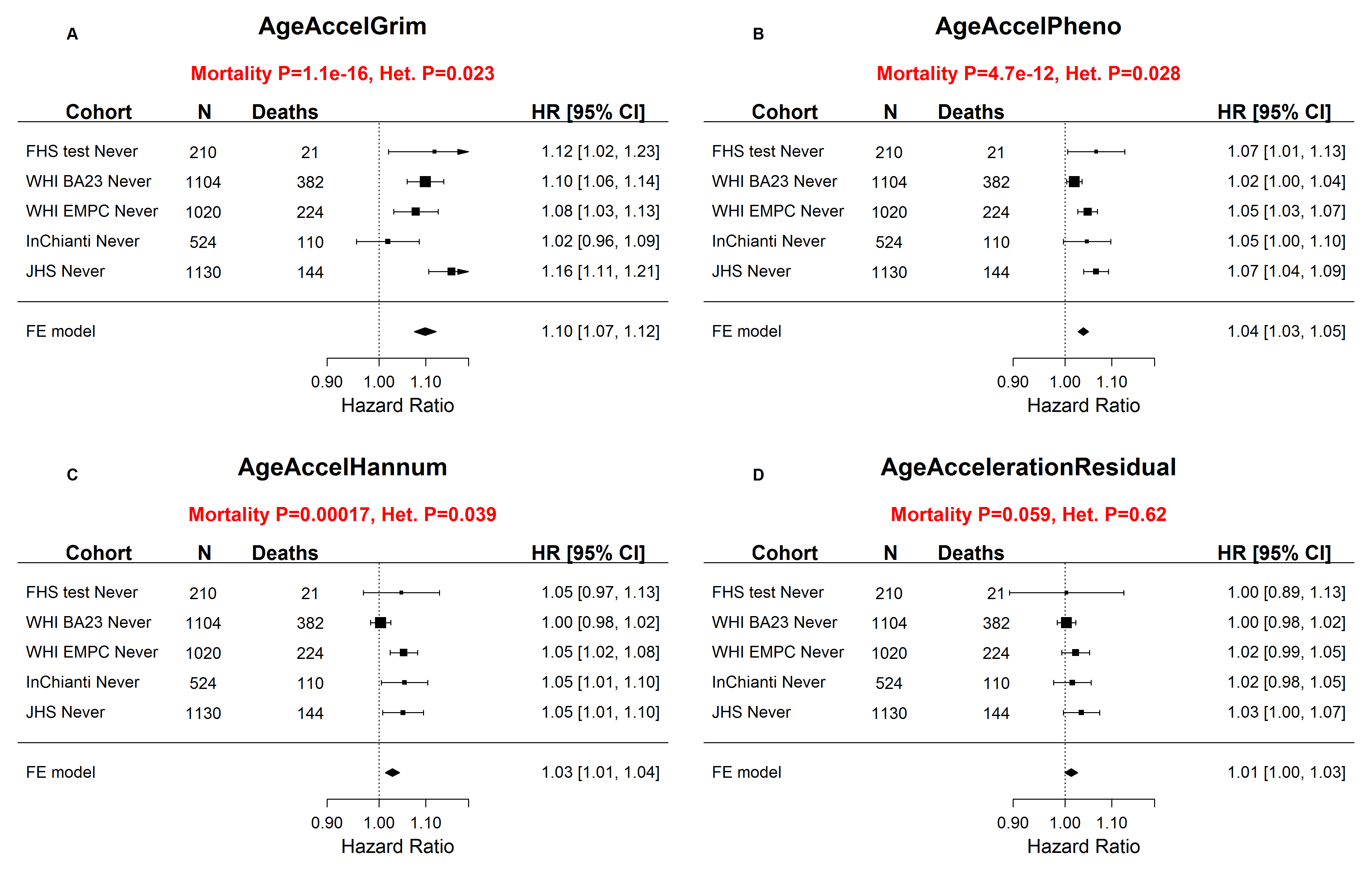


#

# Supplementary Figure 14. Comparing measures of epigenetic age acceleration with respect to predicting time-to-death among smokers.

Here we focus on former or current smokers. Each panel presents a meta analysis forest plot for combining hazard ratios predicting time-to-death based on an epigenetic measure of age acceleration (reported in the figure heading) across different strata formed by racial group within cohort. Each row reports a hazard ratio (for time-to-death due to all cause mortality) and a 95% confidence interval resulting from a Cox regression model in each of 9 strata (defined by cohort and racial groups). Panels A-D report results for (A) AgeAccelGrim, (B) age-adjusted DNAm PhenoAge (AgeAccelPheno) [28], (C) age-adjusted DNAm age based on Hannum et al. (AgeAccelHannum) [27], and (D) age adjusted DNAm age based on Horvath (AgeAccelerationResidual) [24]. The meta analysis p-value (colored in red) should be used when it comes to comparing the predictive accuracy of the different measures. It would not be appropriate to compare the hazard ratios directly because the 4 respective measures of age acceleration have different distributions and are scaled differently. Each hazard ratio (HR) corresponds to a 1 year increase in the respective measure of age acceleration. A non-significant p value of the heterogeneity Cochran’s Q test (Het.) is desirable.


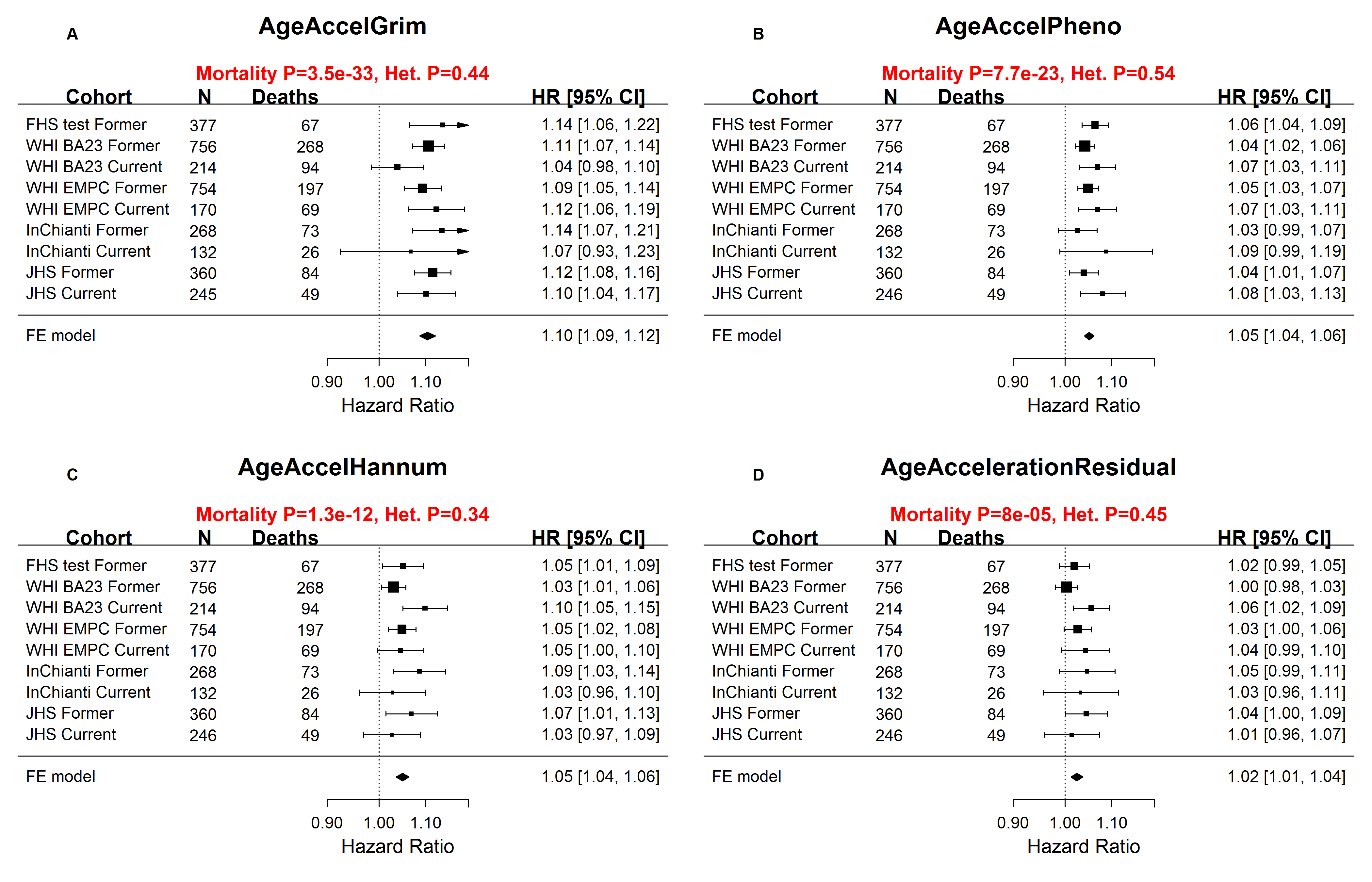


#

# Supplementary Figure 15. Kaplan Meier plots of individuals who age slowly/quickly according to different measures of epigenetic age acceleration.

Each panel depicts two Kaplan Meier plots for survival distributions, i.e. the probability of being alive (y-axis) at a given time after the blood draw (x-axis). The two plots/lines correspond to two groups of individuals: fast epigenetic agers (defined as being in the top 20% percentile of epigenetic age acceleration) and slow epigenetic agers (defined to be in the bottom 20% percentile). Each row reports the results for a different measure of epigenetic age acceleration: first row (A-E) AgeAccelGrim, second row (F-J) AgeAccelPheno, third row (K-O) AgeAccelHannum (based on Hannum 2013), fourth row (P-T) AgeAccelerationResidual (based on Horvath 2013). Columns correspond to the different data sets (corresponding to sub-studies within cohorts). The results cross studies were combined by Stouffer’s method. When it comes to detecting a different mortality risk between fast and slow agers, AgeAccelGrim has the best performance (Stouffer's meta analysis P=6.4E-38), followed by AgeAccelPheno (P=5.7E-21), AgeAccelHannum (P= 1.3E-5) and AgeAccelResidual (P=0.17).


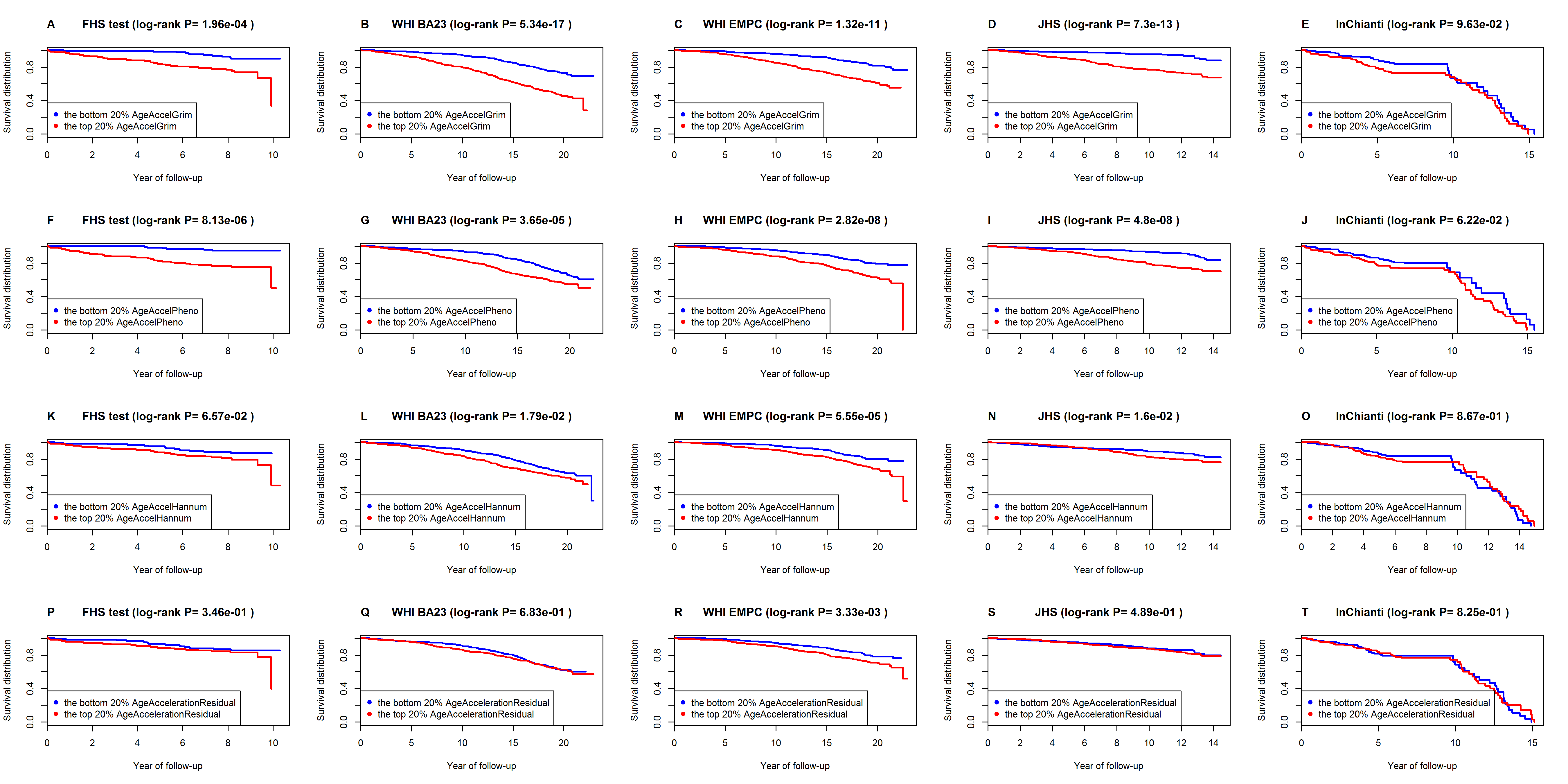


# Supplementary Figure 16. Comparing measures of epigenetic age acceleration wrt. comorbidity count.

Each panel reports a meta analysis forest plot for combining regression coefficients between the comorbidity count (number of morbidities at the time of blood draw) and the DNAm based biomarker (reported in the figure heading) across different strata, which are formed by racial group within cohort. Panel A-D report results for age-adjusted measures of (A) DNAmGrimAge (AgeAccelGrim), (B) DNAm PhenoAge (AgeAccelPheno) [28], (C) DNAm age based on Hannum et al. (AgeAccelHannum) [27], and D) DNAm age based on Horvath (AgeAccelResidual) [24]. Stouffer's meta analysis p-value (colored in red) should be used when it comes to comparing the sensitivity of the different measures. It would not be appropriate to compare the coefficient values directly because the 4 respective measures of age acceleration have different distributions. Each coefficient corresponds to a 1 year increase in the respective measure of age acceleration. As DNAm PhenoAge was trained by the InChianti cohort, we removed the cohort and repeated the analysis. It showed that AgeAccelGrim (P=1.1E-16) and AgeAccelPheno (P=8.5E-18) had similar performance in terms of level of significance, followed by AgeAccelHannum (2.1E-07) and AgeAccelrationResidual (P=1.4E-06).


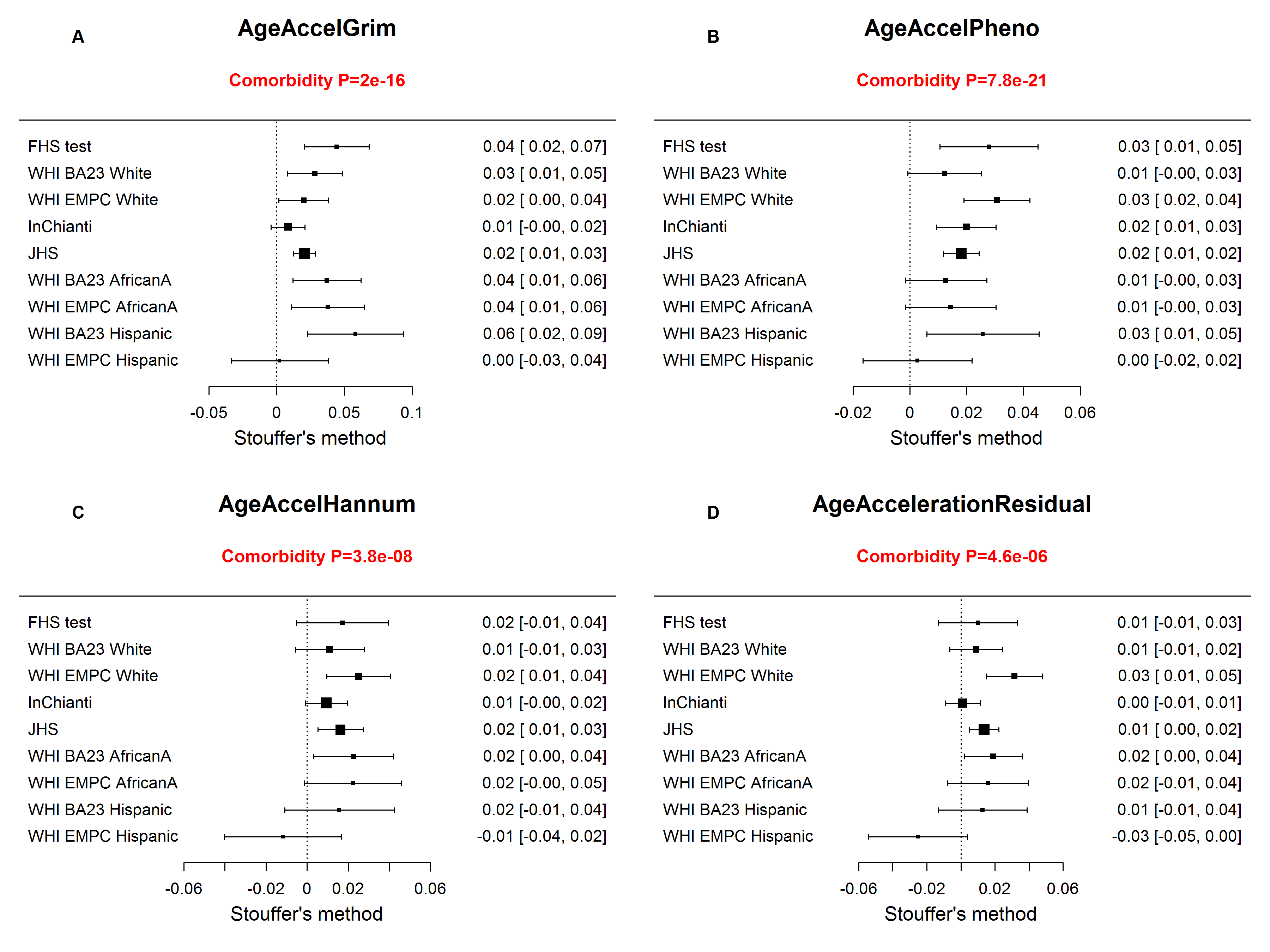


# Supplementary Figure 17. Comparing measures of epigenetic age acceleration wrt. predicting time-to-coronary heart disease.

Each panel presents a meta analysis forest plot for combining hazard ratios predicting time-to-CHD based on an epigenetic measure of age acceleration (reported in the figure heading) across different strata formed by racial group within cohort. Each row reports a hazard ratio (for time-to-CHD) and a 95% confidence interval resulting from a Cox regression model in each of the strata (defined by cohort and racial groups). Panels A-D report results for (A) AgeAccelGrim, (B) age-adjusted DNAm PhenoAge (AgeAccelPheno) [28], (C) age-adjusted DNAm age based on Hannum et al. (AgeAccelHannum) [27], and (D) age adjusted DNAm age based on Horvath (AgeAccelResidual) [24]. The meta analysis p-value (colored in red) should be used when it comes to comparing the predictive accuracy of the different measures. It would not be appropriate to compare the hazard ratios directly because the 4 respective measures of age acceleration have different distributions. Each hazard ratio (HR) corresponds to a 1 year increase in the respective measure of age acceleration. A non-significant p value of the heterogeneity test (Het., Cochran's Q test) is desirable.


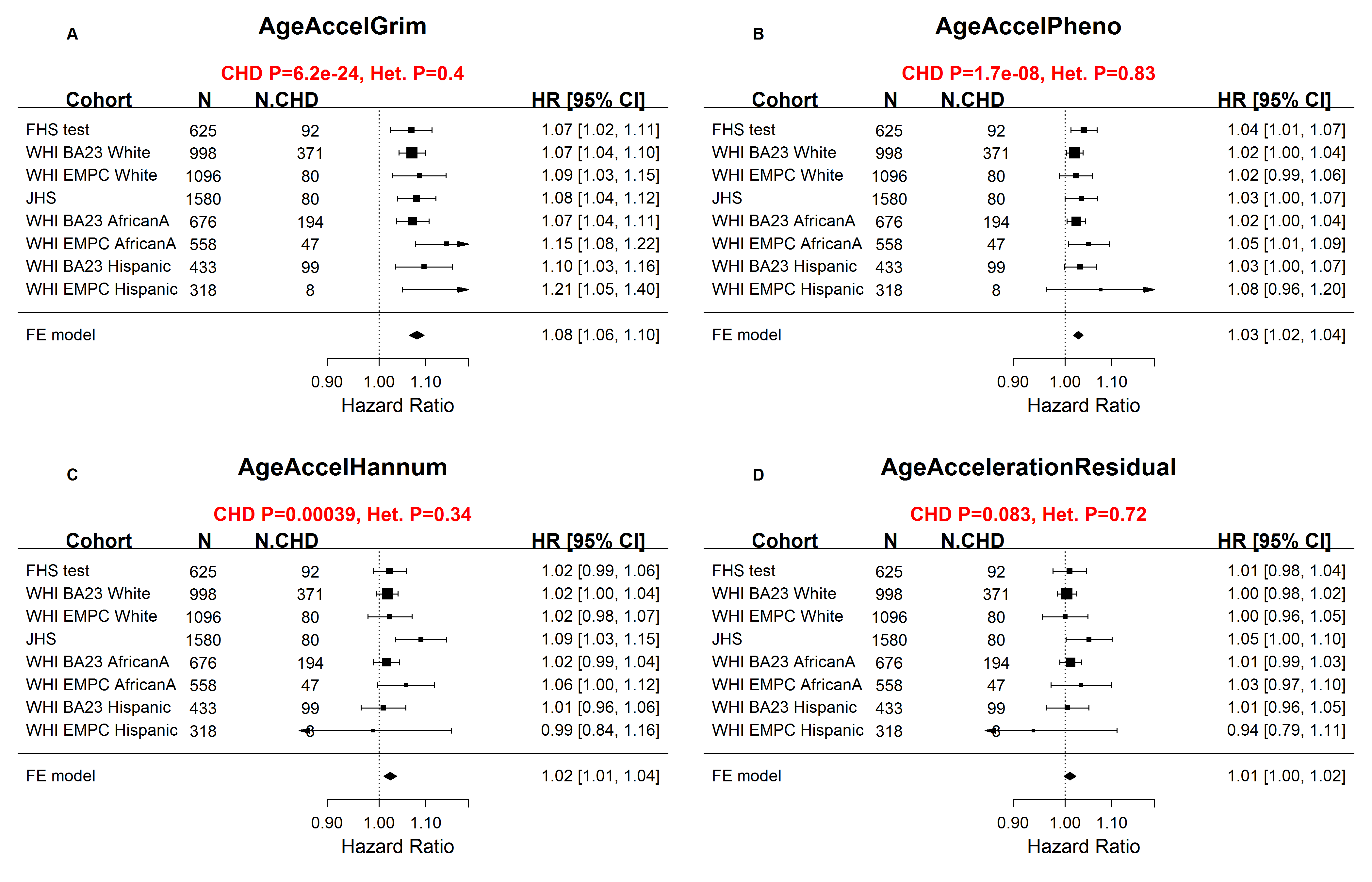


# Supplementary Figure 18. Comparing measures of epigenetic age acceleration wrt. predicting time-to-coronary heart disease among never-smokers.

Here we restrict the analysis to individuals who never smoked before the blood draw. Each panel presents a meta analysis forest plot for combining hazard ratios predicting time-to-CHD based on an epigenetic measure of age acceleration (reported in the figure heading) across different strata formed by racial group within cohort. Each row reports a hazard ratio (for time-to-CHD) and a 95% confidence interval resulting from a Cox regression model in each of the strata (defined by cohort and racial groups). Panels A-D report results for (A) AgeAccelGrim, (B) age-adjusted DNAm PhenoAge (AgeAccelPheno) [28], (C) age-adjusted DNAm age based on Hannum et al. (AgeAccelHannum) [27], and (D) age adjusted DNAm age based on Horvath (AgeAccelResidual) [24]. The meta analysis p-value (colored in red) should be used when it comes to comparing the predictive accuracy of the different measures. It would not be appropriate to compare the hazard ratios directly because the 4 respective measures of age acceleration have different distributions and are scaled differently. Each hazard ratio (HR) corresponds to a 1 year increase in the respective measure of age acceleration. A non-significant p value of the heterogeneity test (Het., Cochran's Q test) is desirable.


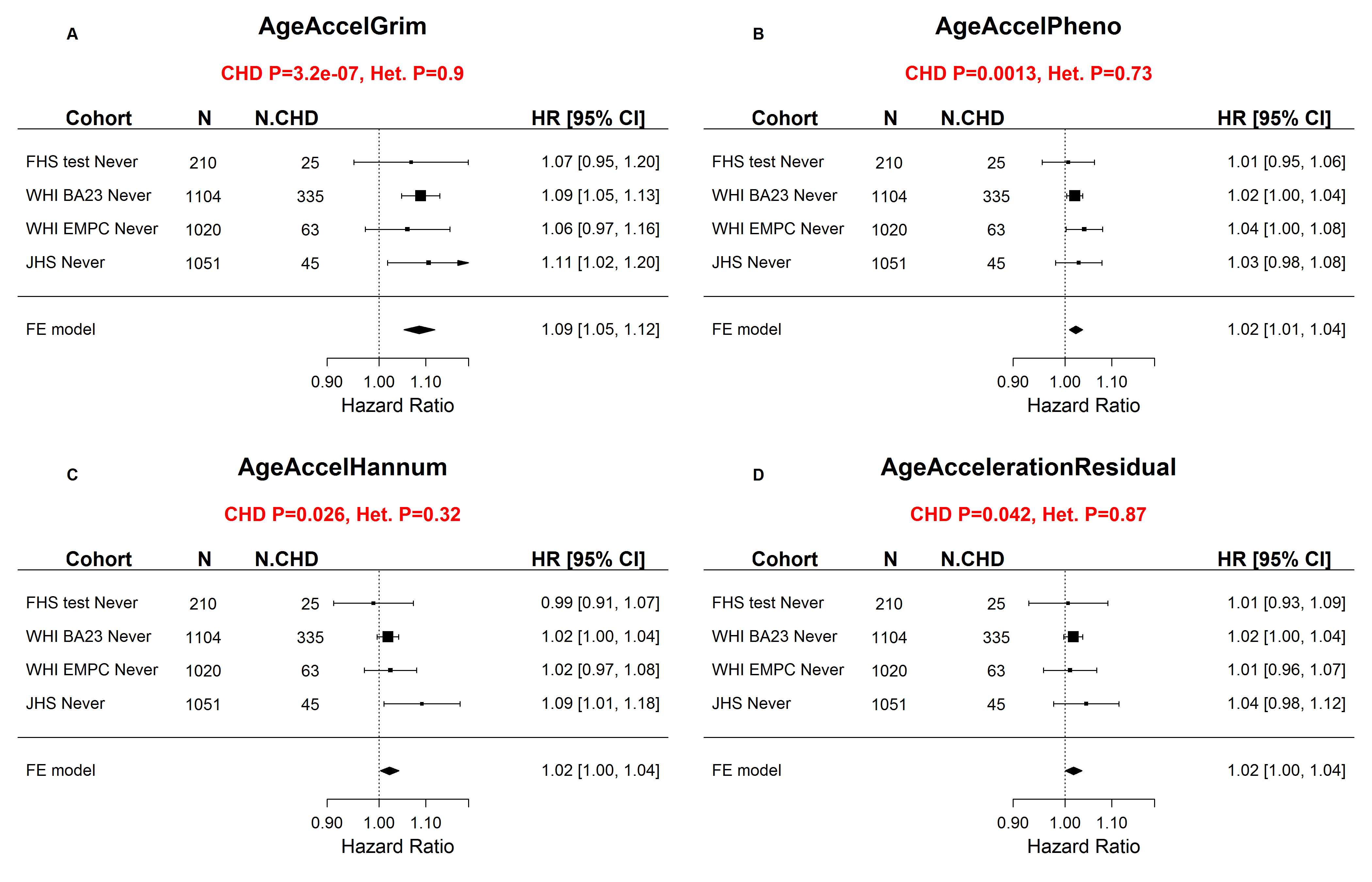


# Supplementary Figure 19. Comparing measures of epigenetic age acceleration wrt. predicting time-to-coronary heart disease among smokers.

Here we restrict the analysis to individuals who were former or current smokers at the time of the blood draw. Each panel presents a meta analysis forest plot for combining hazard ratios predicting time-to-CHD based on an epigenetic measure of age acceleration (reported in the figure heading) across different strata formed by racial group within cohort. Each row reports a hazard ratio (for time-to-CHD) and a 95% confidence interval resulting from a Cox regression model in each of the strata (defined by cohort and racial groups). Panels A-D report results for (A) AgeAccelGrim, (B) age-adjusted DNAm PhenoAge (AgeAccelPheno) [28], (C) age-adjusted DNAm age based on Hannum et al. (AgeAccelHannum) [27], and (D) age adjusted DNAm age based on Horvath (AgeAccelResidual) [24]. The meta analysis p-value (colored in red) should be used when it comes to comparing the predictive accuracy of the different measures. It would not be appropriate to compare the hazard ratios directly because the 4 respective measures of age acceleration have different distributions and are scaled differently. Each hazard ratio (HR) corresponds to a 1 year increase in the respective measure of age acceleration. A non-significant p value of the heterogeneity test (Het., Cochran's Q test) is desirable.


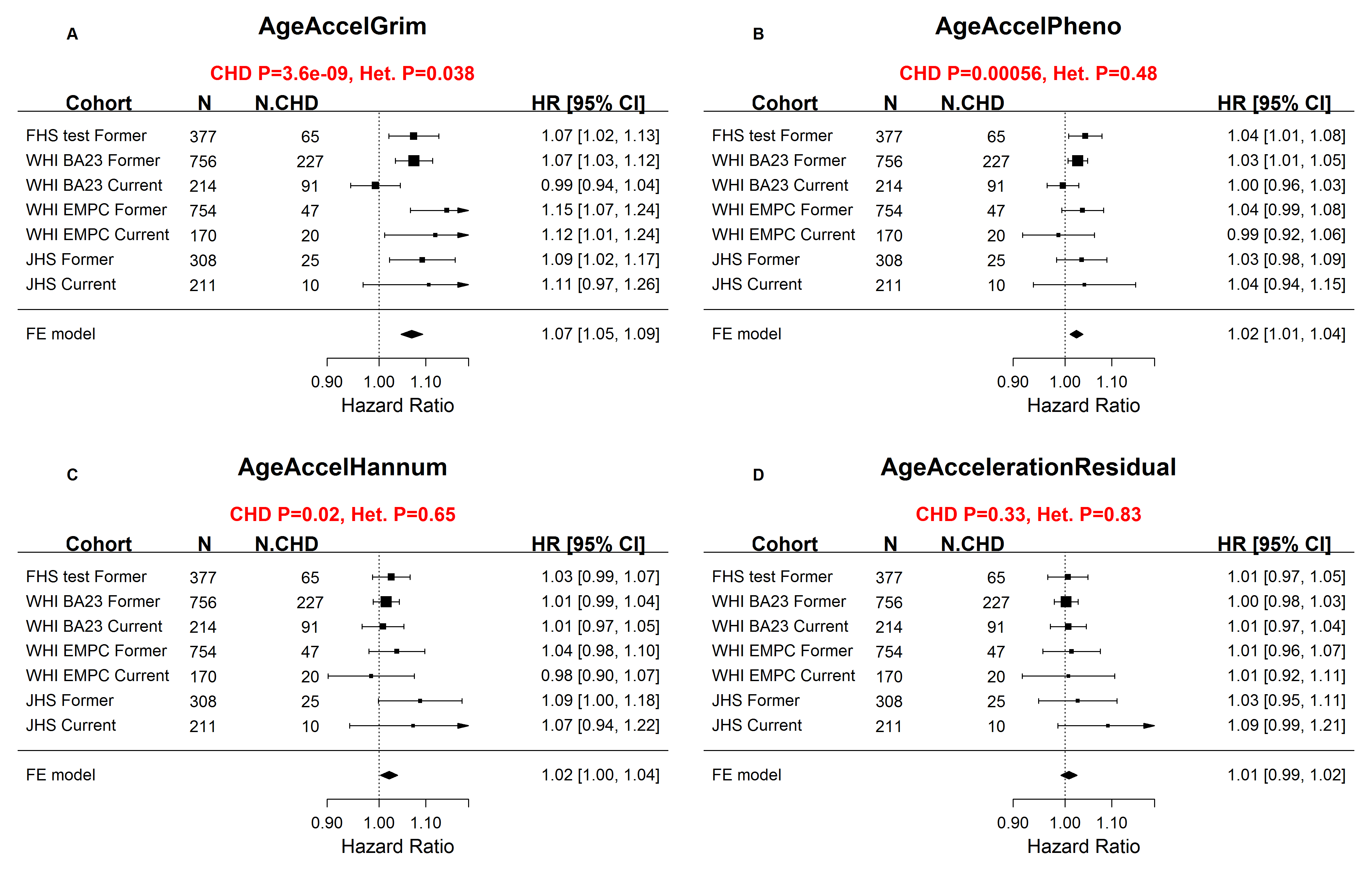


# Supplementary Figure 20. Comparing measures of epigenetic age acceleration wrt. predicting time-to-cancer .

Each panel depicts a meta analysis forest plot for the hazard ratio (HR) in predicting time-to-cancer based on an epigenetic measure of age acceleration (reported in the heading). Panels A-D report results for (A) AgeAccelGrim, (B) age-adjusted DNAm PhenoAge (AgeAccelPheno) [28], (C) age-adjusted DNAm age based on Hannum et al. (AgeAccelHannum) [27], and (D) age adjusted DNAm age based on Horvath (AgeAccelResidual) [24]. The meta analysis p-value (colored in red) should be used when it comes to comparing the different measures. It would not be appropriate to compare the odds ratios directly because the 4 respective measures of age acceleration have different distributions. Each odds ratio corresponds to a 1 year increase in the respective measure of age acceleration. A non-significant p value of the heterogeneity test (Het., Cochran's Q test) is desirable.


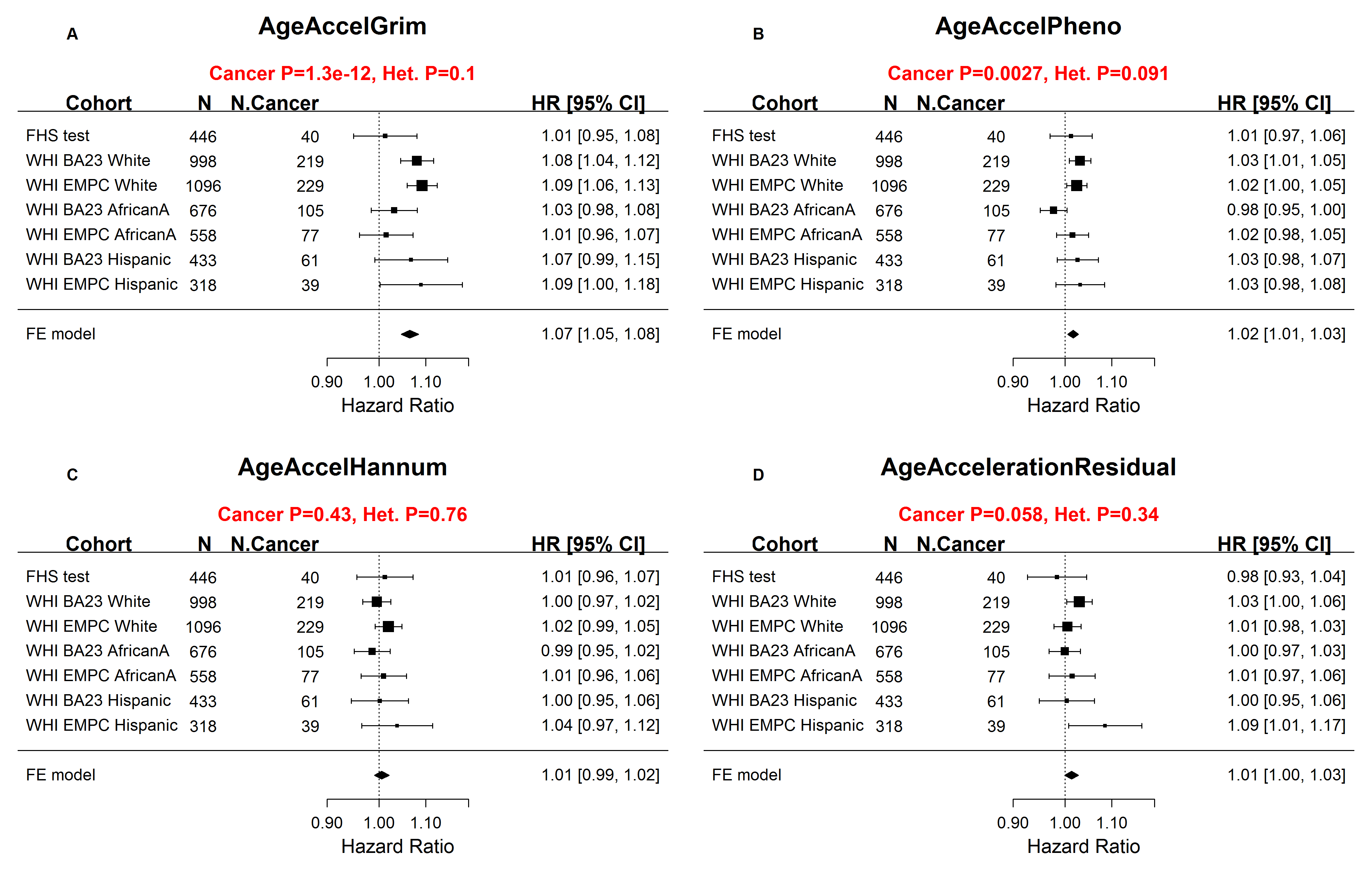


#

# Supplementary Figure 21. Comparing measures of epigenetic age acceleration wrt. age-at-menopause.

Each panel reports a meta analysis forest plot for combining regression coefficients between age-at-menopause (independent variable, in women) and the DNAm based biomarker (dependent variable, as reported in the figure heading) across different strata, which are formed by racial group within cohort. Panel A-D report results for age-adjusted measures of (A) DNAmGrimAge (AgeAccelGrim), (B) DNAm PhenoAge (AgeAccelPheno) [28], (C) DNAm age based on Hannum et al. (AgeAccelHannum) [27], and (D) DNAm age based on Horvath (AgeAccelResidual) [24]. The fixed effects meta analysis p-value (colored in red) should be used when it comes to comparing the sensitivity of the different measures. It would not be appropriate to compare the coefficient values directly because the 4 respective measures of age acceleration have different distributions. Each coefficient corresponds to a 1 year later of age at menopause. Overall, these results shows that earlier age at menopause is associated with accelerated epigenetic age acceleration.


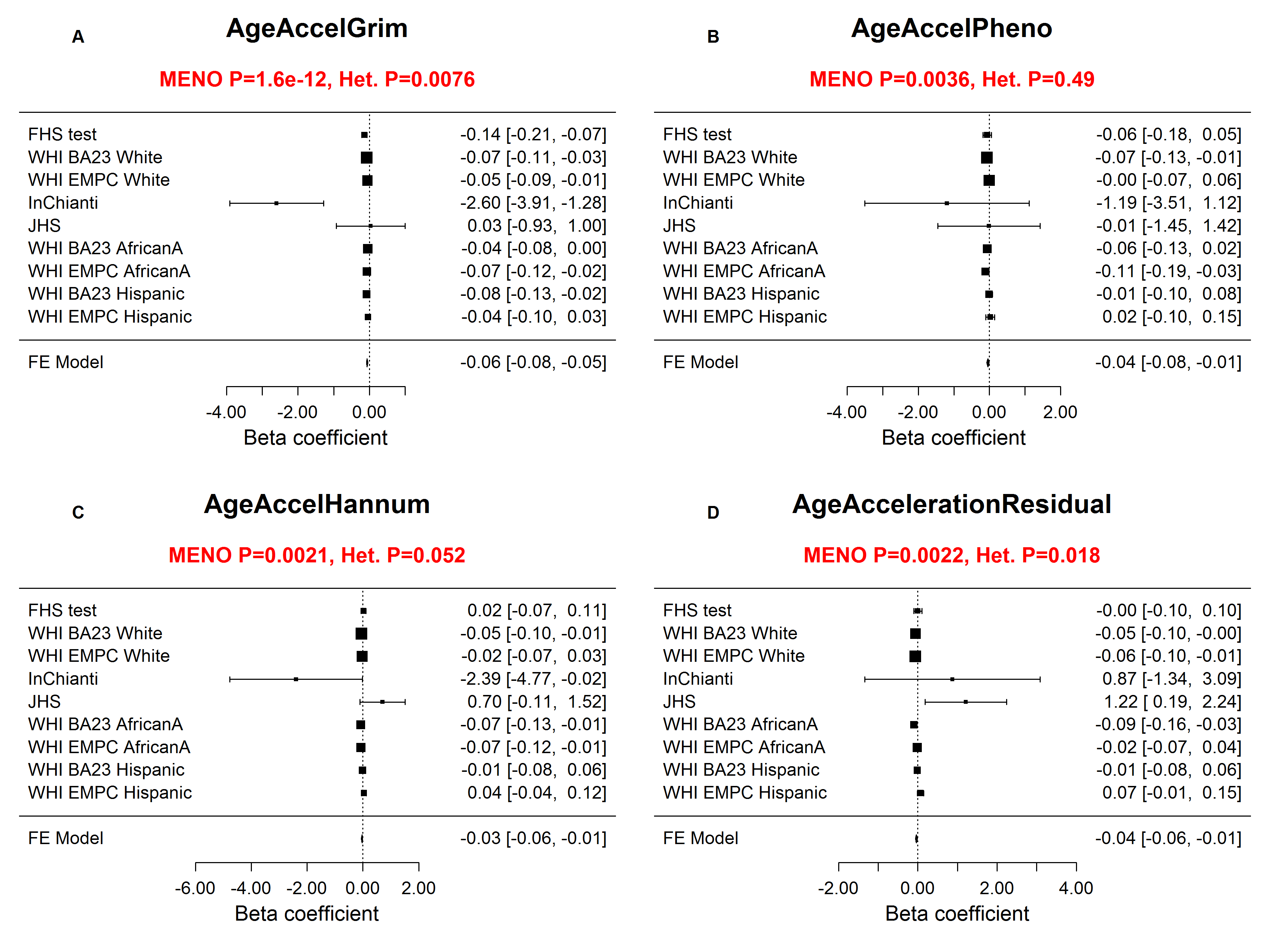


# Supplementary Figure 22. Sensitivity analysis of the meta analysis for age-at-menopause.

The analysis is analogous to the one reported in Supplementary Fig. 21 except that the InChianti study was removed from the analysis because its coefficient value stood out. After removing the InChianti data, the heterogeneity test (Het.) is no longer significant, which is a desirable finding. Note that the earlier results (Supplementary Fig. 21) regarding the superior sensitivity of AgeAccelGrim remain qualitatively the same even after removing the InChianti data.


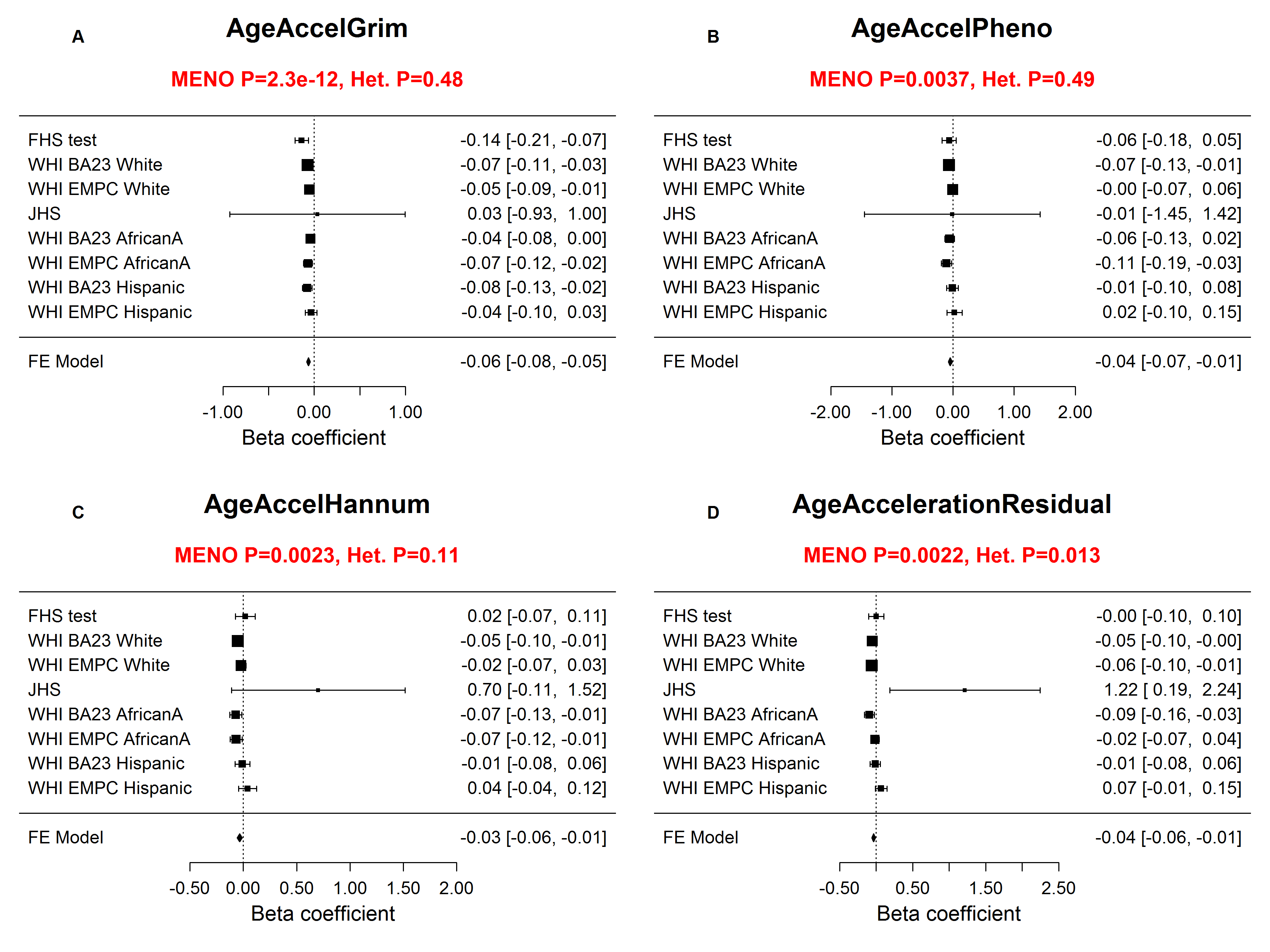


# Supplementary Figure 23. Multivariate Cox regression analysis of time-to-death for epigenetic measures of age acceleration.

Each panel presents a meta analysis forest plot for combining hazard ratios predicting time-to-death based on an epigenetic measure of age acceleration (reported in the figure heading) across different strata formed by racial group within cohort. The survival analysis was based on multivariate Cox regression models, adjusted for chronological age and additional covariates related to demographic characteristics, psychosocial behaviors and clinical covariates. Each row reports a hazard ratio (for time-to-death due to all cause mortality) and a 95% confidence interval resulting from a Cox regression model in each of 9 strata (defined by cohort and racial groups). Panels A-D report results for (A) AgeAccelGrim, (B) age-adjusted DNAm PhenoAge (AgeAccelPheno) [28], (C) age-adjusted DNAm age based on Hannum et al. (AgeAccelHannum) [27], and (D) age adjusted DNAm age based on Horvath (AgeAccelResidual) [24]. The meta analysis p-value (colored in red) should be used when it comes to comparing the predictive accuracy of the different measures. It would not be appropriate to compare the hazard ratios directly because the 4 respective measures of age acceleration have different distributions and are scaled differently. Each hazard ratio (HR) corresponds to a 1 year increase in the respective measure of age acceleration. A non-significant p value of the heterogeneity Cochran’s Q test (Het.) is desirable.


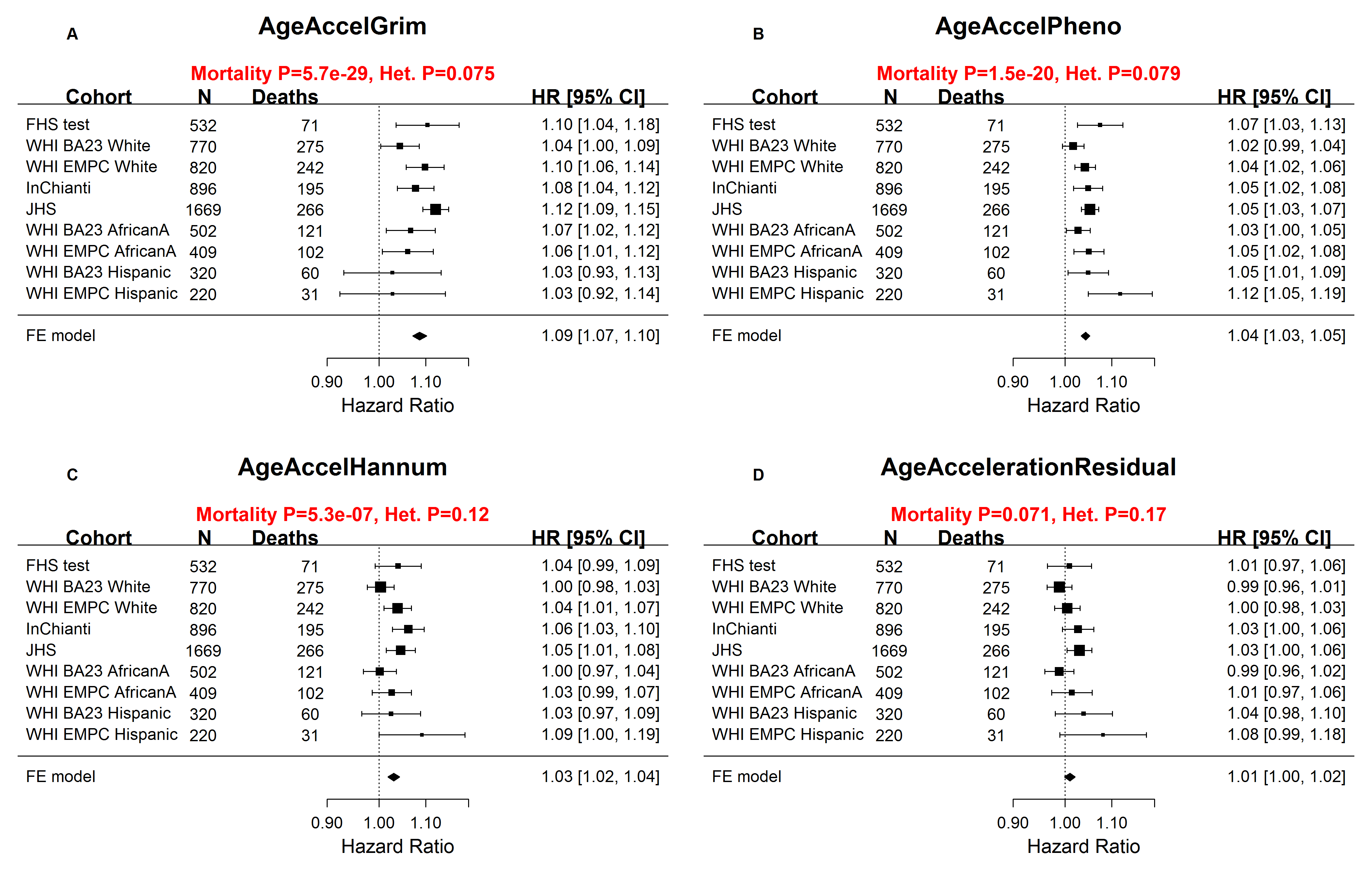


# Supplementary Figure 24. Multivariate Cox regression analysis of time-to-CHD for epigenetic measures of age acceleration.

Each panel presents a meta analysis forest plot for combining hazard ratios predicting time-to-CHD based on an epigenetic measure of age acceleration (reported in the figure heading) across different strata formed by racial group within cohort. The survival analysis was based on multivariate Cox regression models, adjusted for chronological age and additional covariates related to demographic characteristics, psychosocial behaviors and clinical covariates. Each row reports a hazard ratio (for time-to-CHD) and a 95% confidence interval resulting from a Cox regression model in each of the strata (defined by cohort and racial groups). Panels A-D report results for (A) AgeAccelGrim, (B) age-adjusted DNAm PhenoAge (AgeAccelPheno) [28], (C) age-adjusted DNAm age based on Hannum et al. (AgeAccelHannum) [27], and (D) age adjusted DNAm age based on Horvath (AgeAccelResidual) [24]. The meta analysis p-value (colored in red) should be used when it comes to comparing the predictive accuracy of the different measures. It would not be appropriate to compare the hazard ratios directly because the 4 respective measures of age acceleration have different distributions. Each hazard ratio (HR) corresponds to a 1 year increase in the respective measure of age acceleration. A non-significant p value of the heterogeneity test (Het., Cochran's Q test) is desirable.


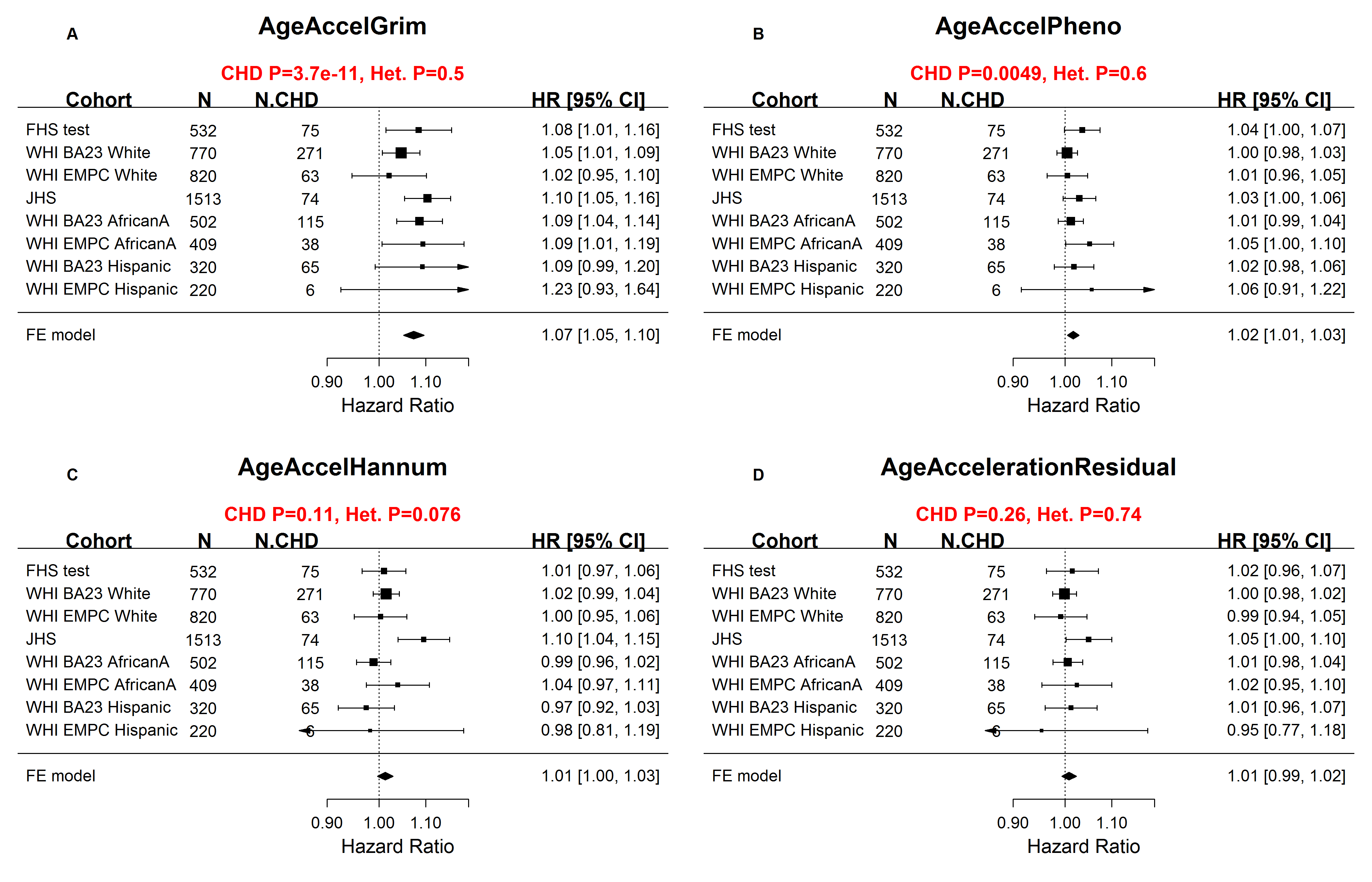


# Supplementary Figure 25. Comparing DNAm GrimAge with single stage mortality predictors wrt. time-to-death.

Each panel presents a meta analysis forest plot for combining hazard ratios predicting time-to-death (due to all cause mortality) based on an epigenetic measure of age acceleration (reported in the figure heading) across different strata formed by racial group within cohort. Each row reports a hazard ratio (for time-to-death due to all cause mortality) and a 95% confidence interval resulting from a Cox regression model in each of 9 strata (defined by cohort and racial groups). (A) Meta analysis of AgeAccelGrim, (B) Meta analysis for AgeAccelMortality, which is an age adjusted version of our single stage mortality estimator, DNAm Mortality, on the basis of 59 CpGs. The direct approach involved in the construction of DNAm Mortality involved again an ElasticNet Cox regression model and subsequent linear transformation of the mortality risk to ensure that the values of DNAm Mortality are in units of years (**Methods**). We used the mortality predictor of Zhang (2017) [29] to define two single stage mortality risk estimators denoted by DNAm Zhang and DNAm ZhangScore, respectively (**Methods**). (C,D) Meta analysis for the age adjusted versions of DNAmZhang (AgeAccelZhang, panel (C) and DNAmZhangScore (panel (D) [29]. Of the 10 CpGs involved in Zhang’s mortality risk model, cg06126421 and cg23665802 were absent from the JHS data and were replaced by the median values calculated in the FHS training data. Panels E-H list the results of a sensitivity analysis that omitted the JHS cohort from the meta analysis. The meta analysis p-value (colored in red) should be used when it comes to comparing the predictive accuracy of the different measures. It would not be appropriate to compare the hazard ratios directly because the 4 respective measures of age acceleration have different distributions. A non-significant p value of the heterogeneity test (Het., Cochran's Q test) is desirable.


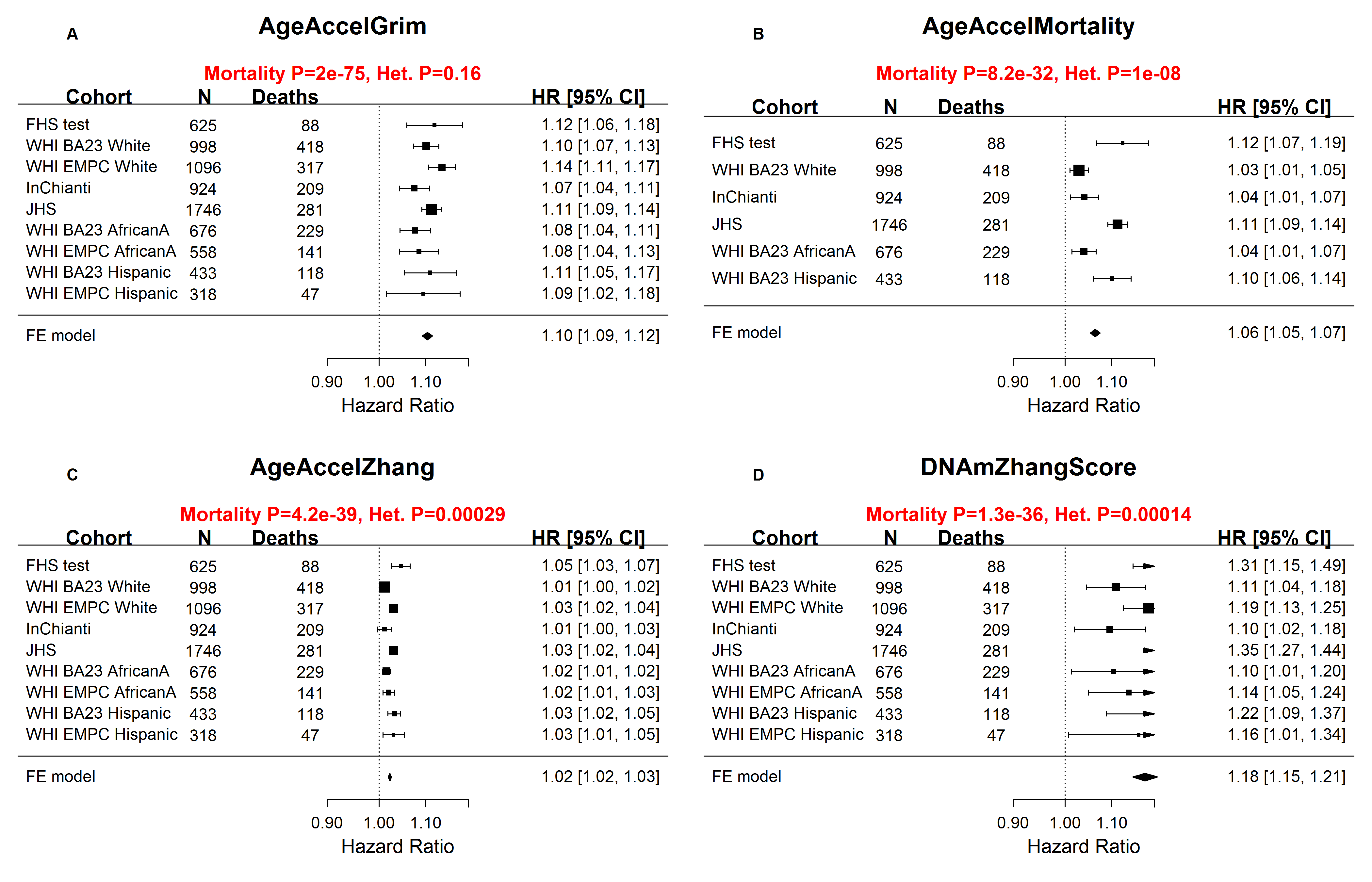


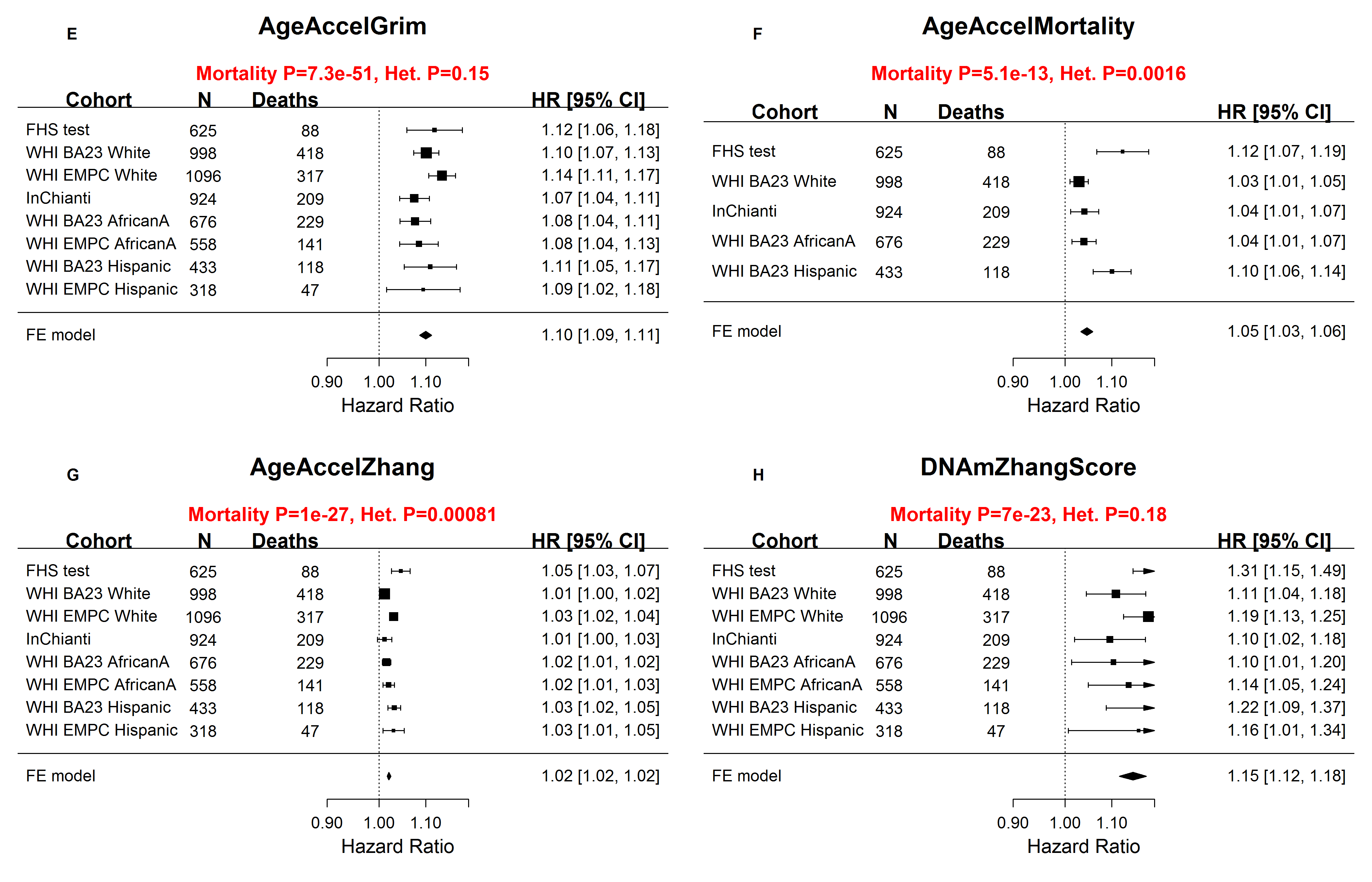
**Sensitivity Analysis: Results after omitting JHS**

# Supplementary Figure 26. Comparing DNAm GrimAge with single stage mortality predictors wrt. time-to-coronary heart disease.

Each panel presents a meta analysis forest plot for combining hazard ratios predicting time-to-death (due to all cause mortality) based on an epigenetic measure of age acceleration (reported in the figure heading) across different strata formed by racial group within cohort. Each row reports a hazard ratio (for time-to-death due to all cause mortality) and a 95% confidence interval resulting from a Cox regression model in each of 9 strata (defined by cohort and racial groups). A) Meta analysis of AgeAccelGrim, B) Meta analysis for AgeAccelMortality, which is an age adjusted version of our single stage mortality estimator, DNAm Mortality, on the basis of 59 CpGs. The direct approach involved in the construction of DNAm Mortality involved again an ElasticNet Cox regression model and subsequent linear transformation of the mortality risk to ensure that the values of DNAm Mortality are in units of years (Methods). We used the mortality predictor of Zhang (2017) [29] to define two single stage mortality risk estimators denoted by DNAm Zhang and DNAm ZhangScore, respectively (Methods). C,D) Meta analysis for the age adjusted versions of DNAmZhang (AgeAccelZhang, panel C) and DNAmZhangScore (panel D) [29]. Of the 10 CpGs involved in Zhang’s mortality risk model, cg06126421 and cg23665802 were absent from the JHS data and were replaced by the median values calculated in the FHS training data. Panels E-H list the results of a sensitivity analysis that omitted the JHS cohort from the meta analysis. The meta analysis p-value (colored in red) should be used when it comes to comparing the predictive accuracy of the different measures. It would not be appropriate to compare the hazard ratios directly because the 4 respective measures of age acceleration have different distributions. A non-significant p value of the heterogeneity test (Het., Cochran's Q test) is desirable.


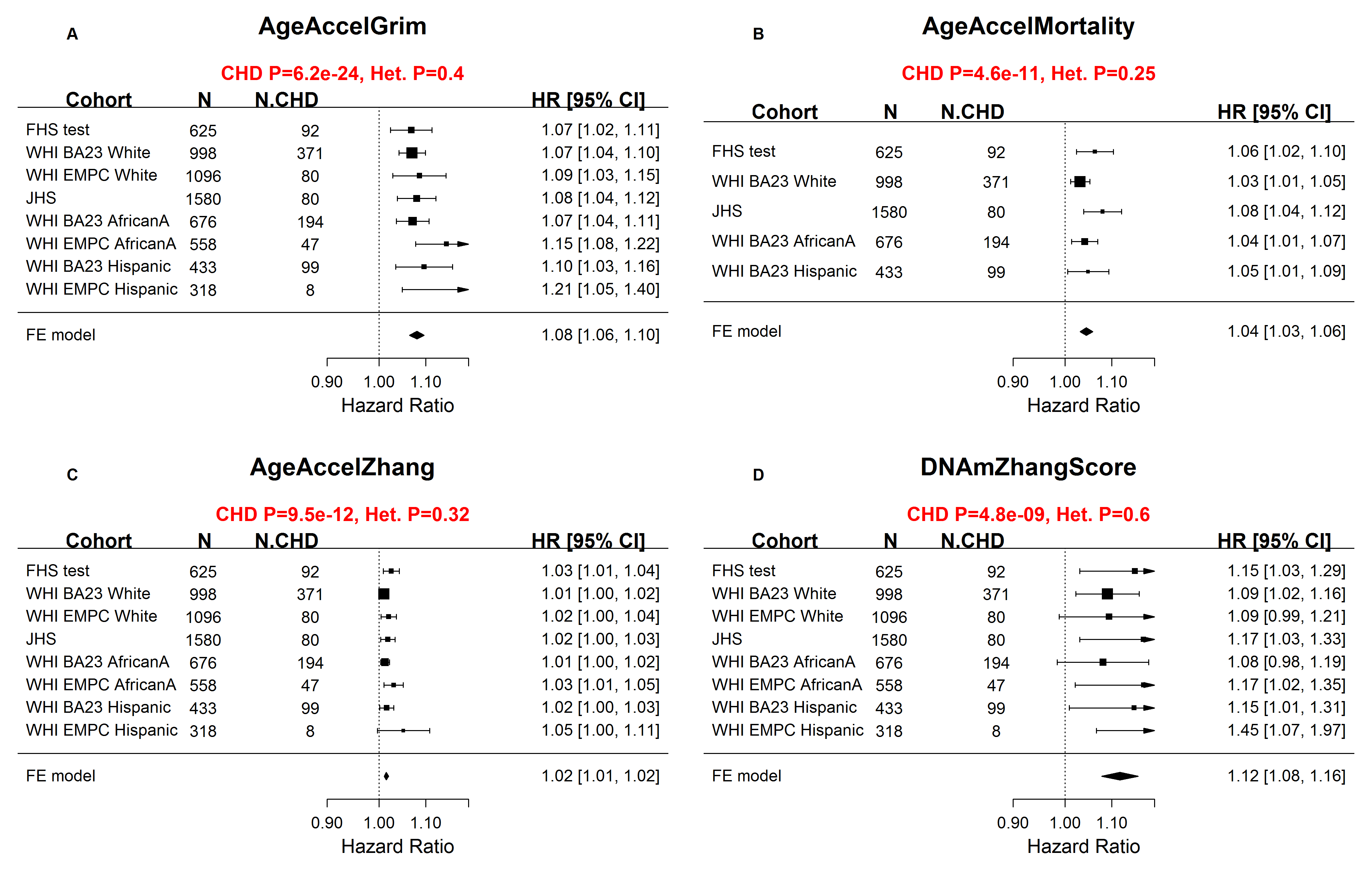


**Sensitivity analysis that omits the JHS cohort**


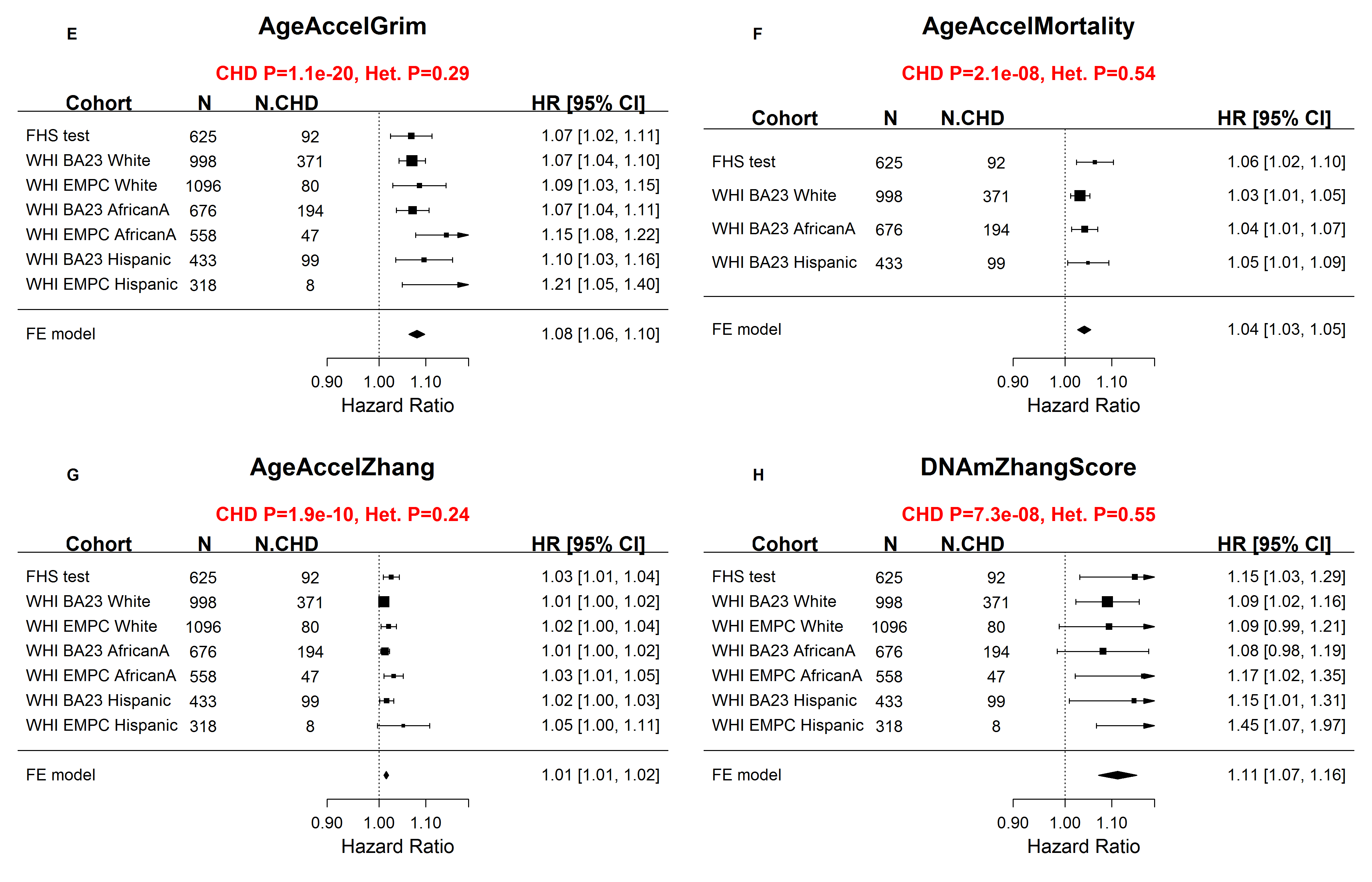


# Supplementary Figure 27. Measures of blood cell composition versus DNAm based biomarkers.

Each panel reports how the respective DNAm based biomarker (heading) relates to 10 imputed measures of blood cell counts. The height of each bar corresponding to the statistical significance level (meta analysis p-value) of an association test between the blood cell measure and the age-adjusted DNAm biomarker. More precisely, the y-axis presents minus logarithm (base 10) transformed meta P values. The numbers displayed on top of each bar are the meta analysis estimates of the correlation coefficients. The association analysis is *not* confounded by chronological age because we used age adjusted DNAm based biomarkers. The fixed effects meta analysis was performed across the validation study sets (N=6003): FHS test, WHI BA23, JHS, and inChianti. Additional analysis was performed on DNAm Leptin stratified by gender, due to its highly correlation with gender (panels J and K).

Abbreviations for cell counts are listed in the following: nature killer (NK), monocyte (MONO) and granulocyte (Gran), CD8pCD28nCD45Ran (CD8.ex for exhausted cytotoxic T cells), and plasma blast (PB). The blood cell counts were imputed based on DNA methylation levels as described in [23, 30] and the **Supplementary Methods section** (above).


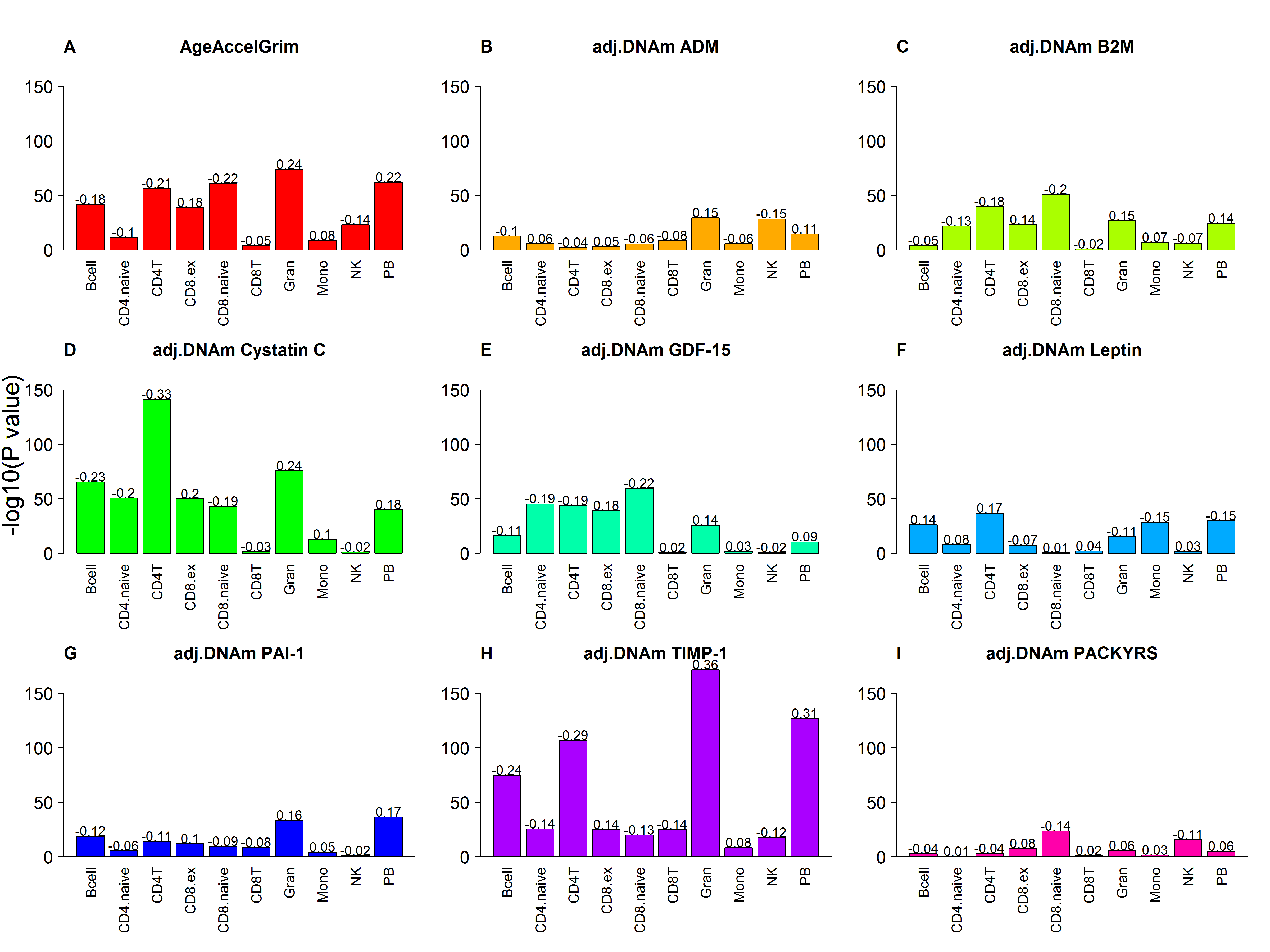


**Blood cell count analysis for age-adjusted DNAm Leptin stratified by gender**


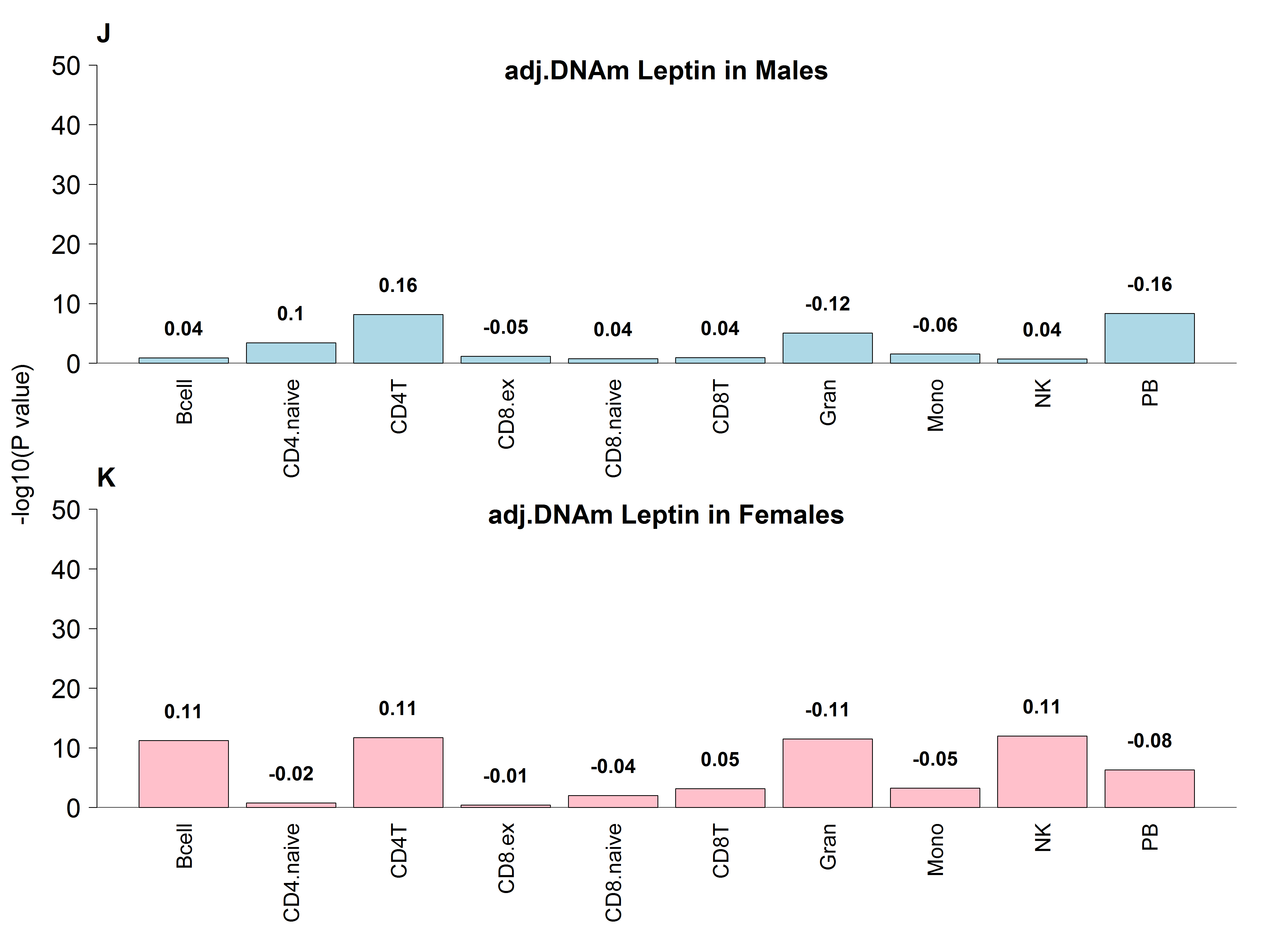


# Supplementary Figure 28. Meta analysis forest plots for predicting time-to-death adjusted for blood cell composition.

Each panel reports a meta analysis forest plot for combining hazard ratios predicting time-to-death based on a DNAm based biomarker (reported in the figure heading) across different strata formed by racial group within cohort. Here we re-conducted the survival analysis as listed in **Figure 3** and adjusted additional 7 imputed blood cell counts: CD8 naïve, CD8pCD28nCD45Ran, plasma blasts, CD4+ T, nature killer cells, monocytes and granulocytes.


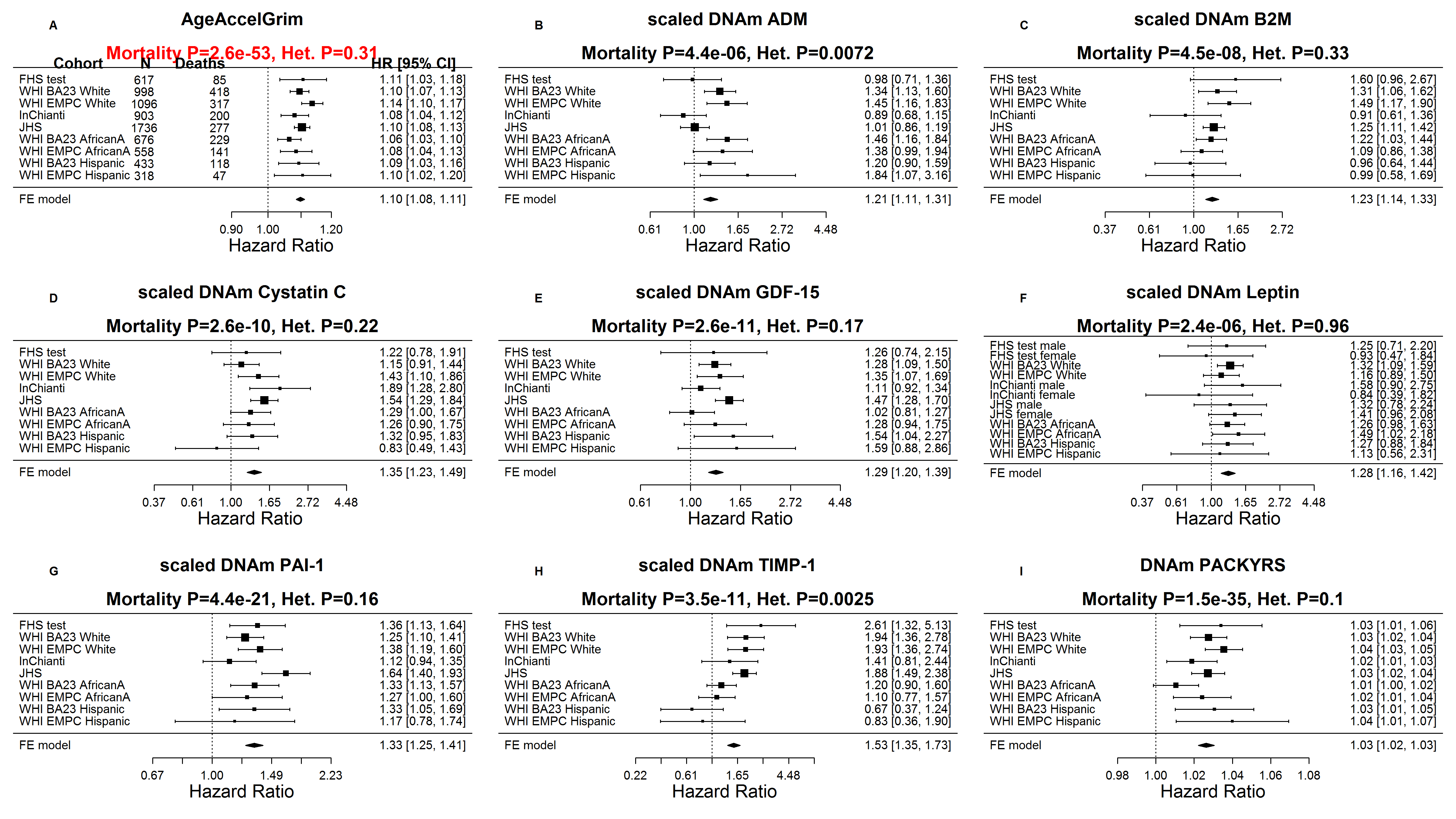


# Supplementary Figure 29. Meta analysis forest plots for predicting time-to-coronary heart disease adjusted for blood cell composition.

Each panel reports a meta analysis forest plot for combining hazard ratios predicting time to CHD and the DNAm based biomarker (reported in the figure heading) across different strata formed by racial groups within cohorts. Here we re-conducted the survival analysis as listed in **Figure 4** and adjusted additional 7 imputed blood cell counts: CD8 naïve, CD8pCD28nCD45Ran, plasma blasts, CD4+ T, nature killer cells, monocytes and granulocytes.


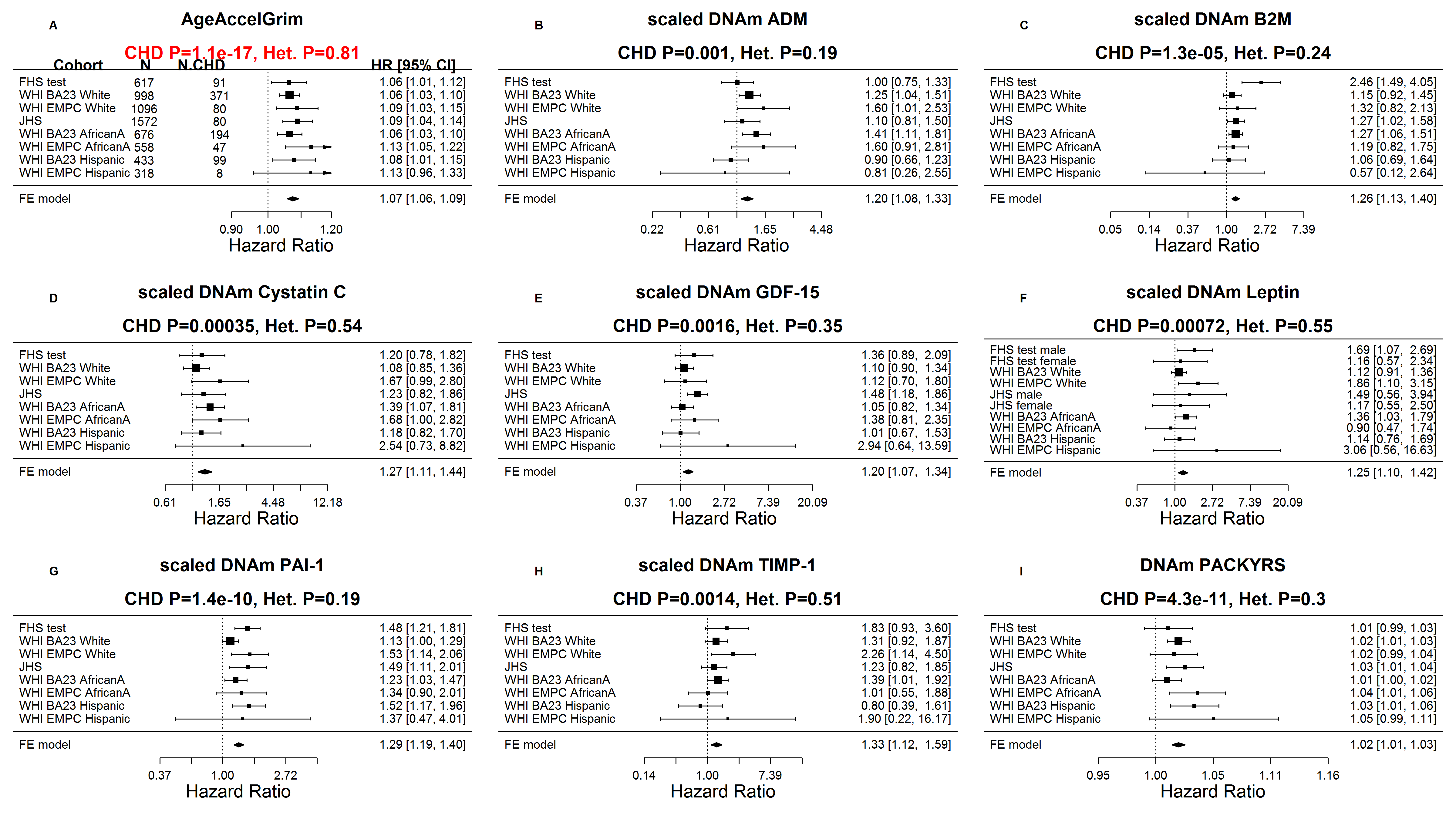


# Supplementary Figure 30. Marginal correlation analysis of lifestyle factors, biomarkers, and age-adjusted DNAm biomarkers in the WHI.

The columns correspond to (age-adjusted versions of) DNAm based biomarkers. The rows correspond to different lifestyle factors and clinical biomarkers. The first column reports the sample size (n) in the WHI. Other columns report the robust correlation coefficient (bicor, based on the biweight midcorrelation coefficient [31]), and a corresponding correlation test p-value. The bicor correlation coefficients is color coded (blue to red) across its range of [-1, 1]. P-values are color-coded in green (light to dark green scale).

The analysis (rows) are stratified by race/ethnicity: (A) non-Hispanic white (European Ancestry, N up to 2048), (B) African Ancestry (N up to 1180, see panel B and Hispanic ancestry (N up to 719, see panel C).

1. **Non-Hispanic White Postmenopausal Women**


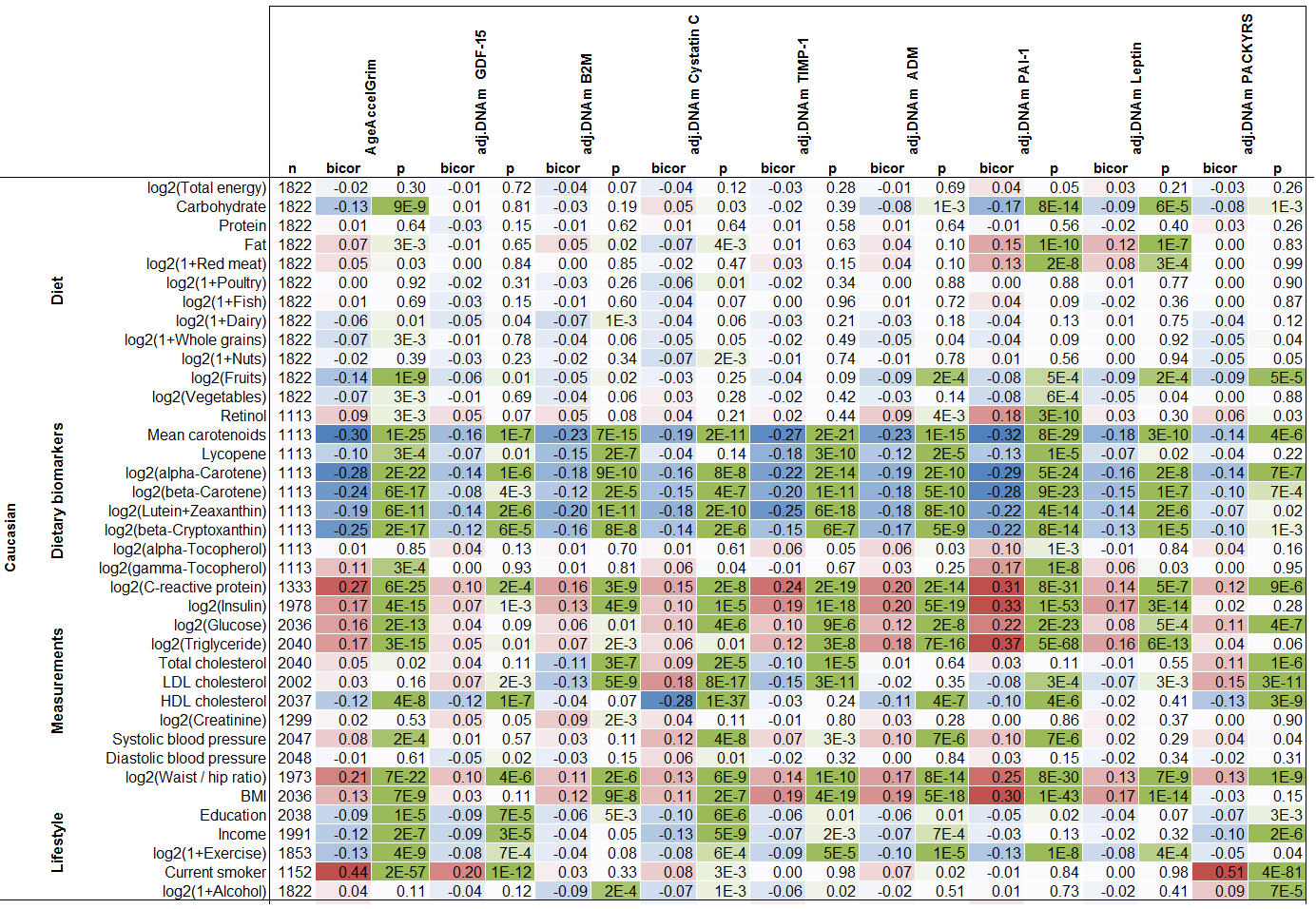


1. **African American Postmenopausal Women**


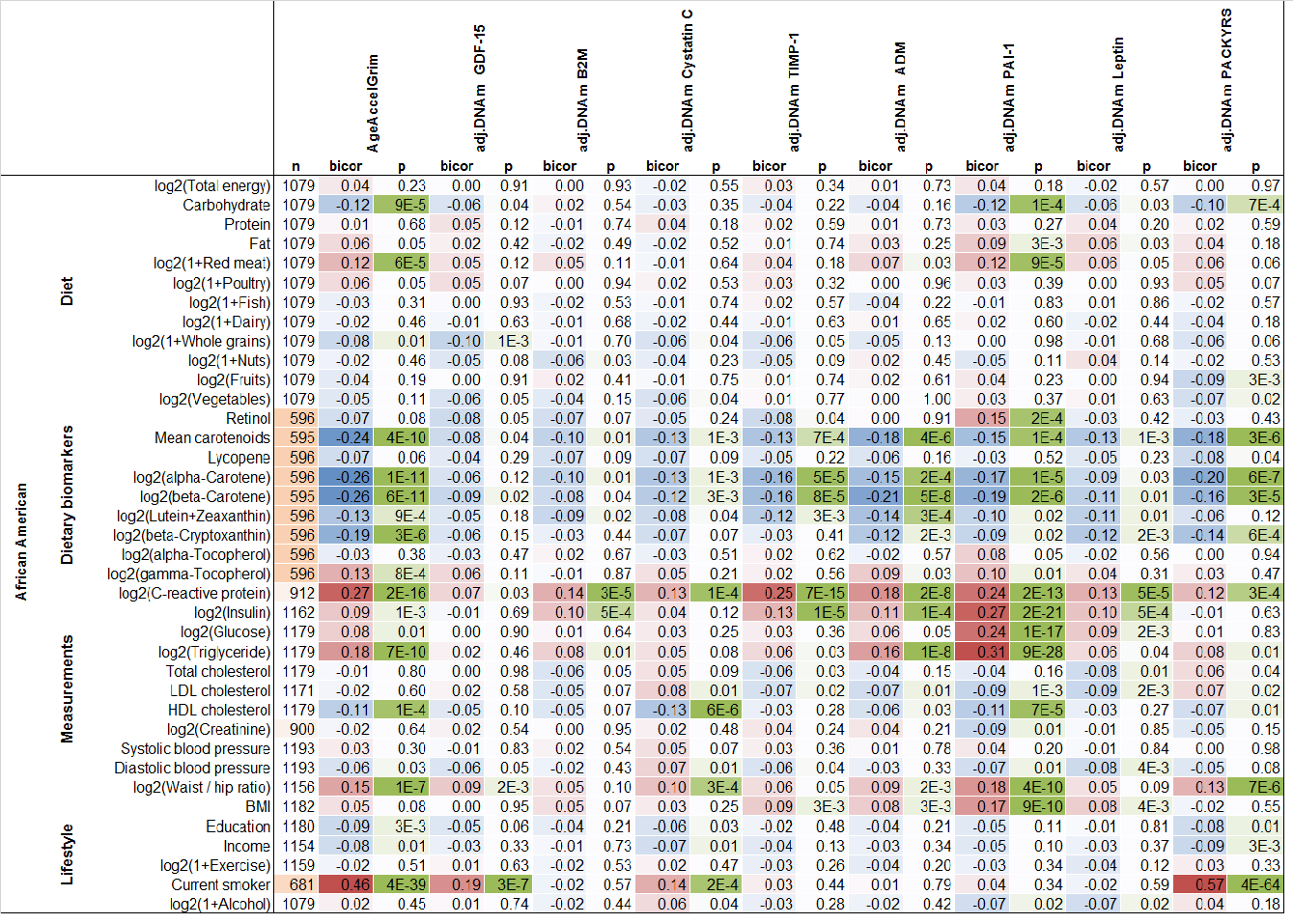


1. **Hispanic Postmenopausal Women**

**
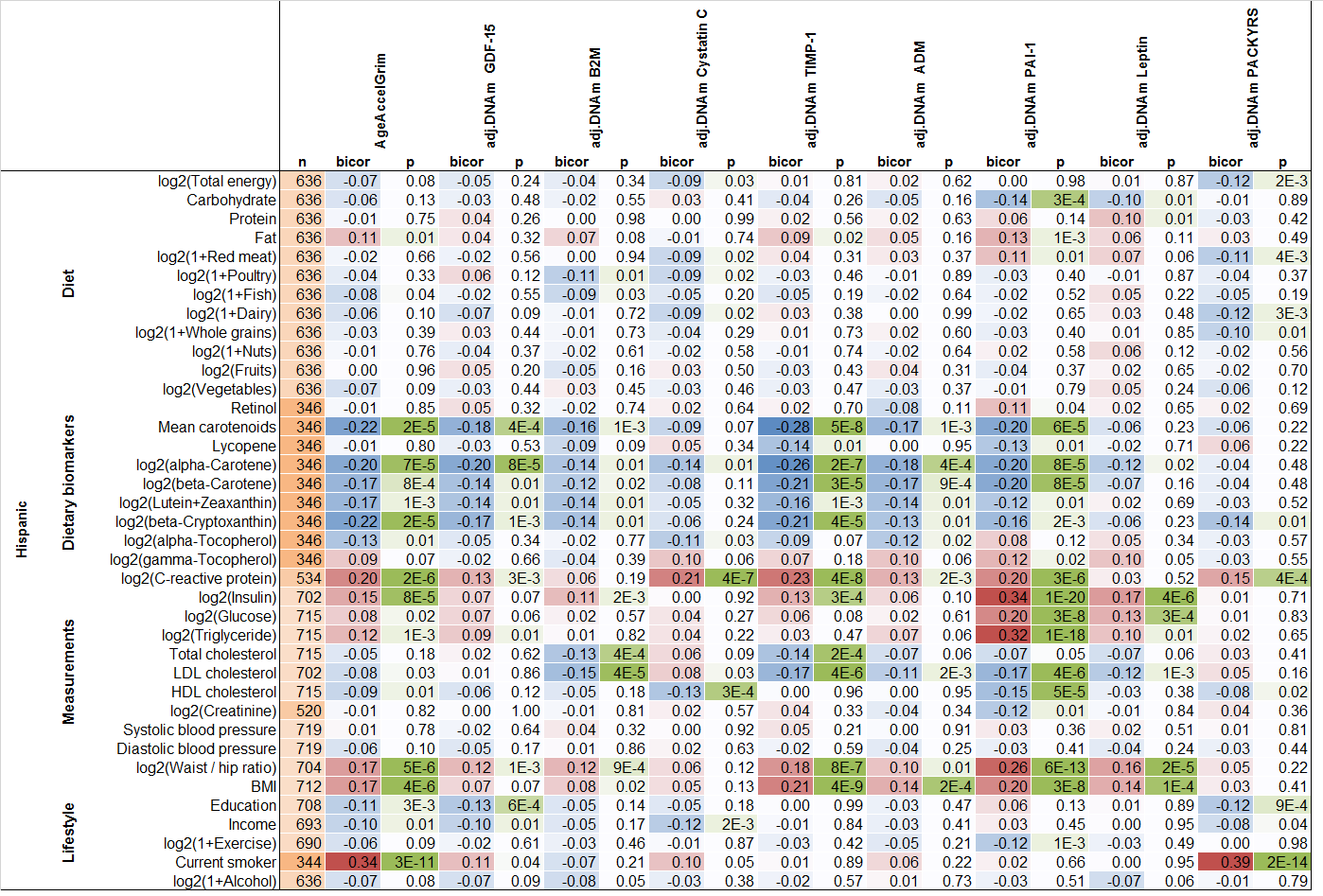
**

# Supplementary Figure 31. Marginal correlation analysis of clinical biomarkers versus age-adjusted DNAm biomarkers in the FHS.

The columns correspond to (age-adjusted versions of ) DNAm based biomarkers. The rows of the table correspond to different clinical biomarkers, e.g. measures of lipid levels, glucose levels, (e.g. Hemoglobin A1C), and various metabolites. As indicated, in the first column, the results were analyzed in all individuals, in females only, and in males only. The columns report the available sample size (n) in the test data from the FHS, the robust correlation coefficient (bicor, based on the biweight midcorrelation coefficient [31]), and a p-value based on a linear effects model that also adjusts for the pedigree structure (implemented in the R package "nlme"). The bicor correlation coefficients is color coded (blue to red) across its range of [-1, 1]. P-values are color-coded in green (light to dark green scale). The fact that total cholesterol exhibits an unexpected negative correlation (*bicor* <0) with AgeAccelGrim and the other age-adjusted DNAm variables might reflect that total cholesterol is the sum of both good (HDL) and bad cholesterol (LDL). This interpretation is supported by the expected positive correlation results for the ratio "Total cholesterol/HDL", which is known to be positively correlated with the risk of coronary heart disease. The fact that the age-adjusted versions of DNAm ADM and DNAm Leptin also showed unexpected correlation patterns with the clinical biomarkers might reflect confounding by sex as can be seen from the sex-stratified analysis. While HDL has a weak positive correlation with age-adjusted DNAm ADM (r=0.14) across all individuals, weak negative correlations can be observed in males (r= -0.12) and females (r= -0.13).


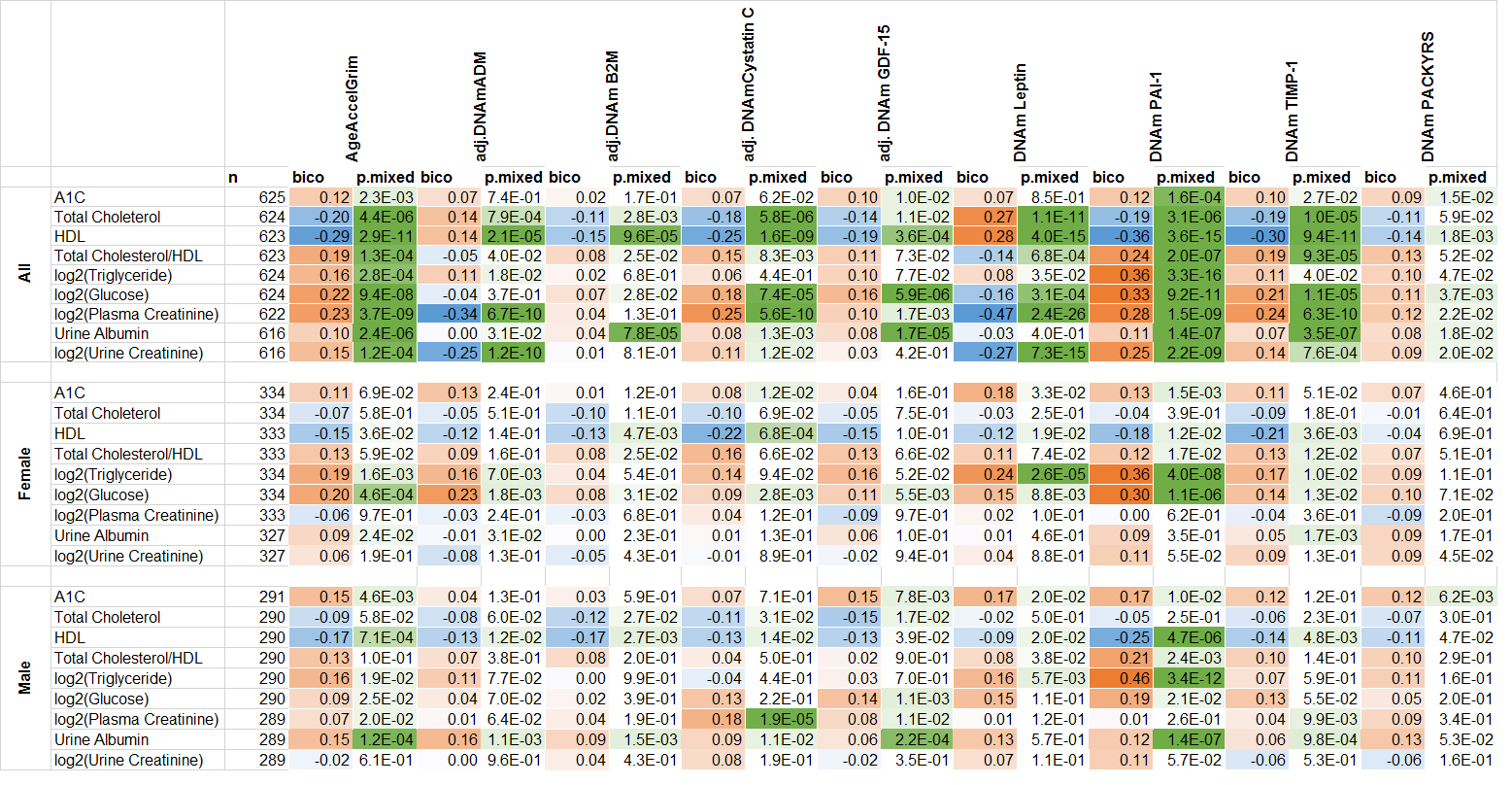


# Supplementary Figure 32. Correlation analysis of chronological age versus CT-scan fatty liver and adipose tissue density in the FHS.

We present the scatter plots of chronological age at computed tomography (CT) scan (x-axis) versus CT-scan derived measures in the FHS. The CT-scan measures included attenuation in liver, spleen, paraspinal muscle, subcutaneous adipose tissue (SAT) and visceral adipose tissue (VAT). Panels A-C, E and G are in Hounsfield (HU) unit, obtained from a linear transformation of attenuation coefficients. Panels D and F are measures of volume in units of cm^3^.


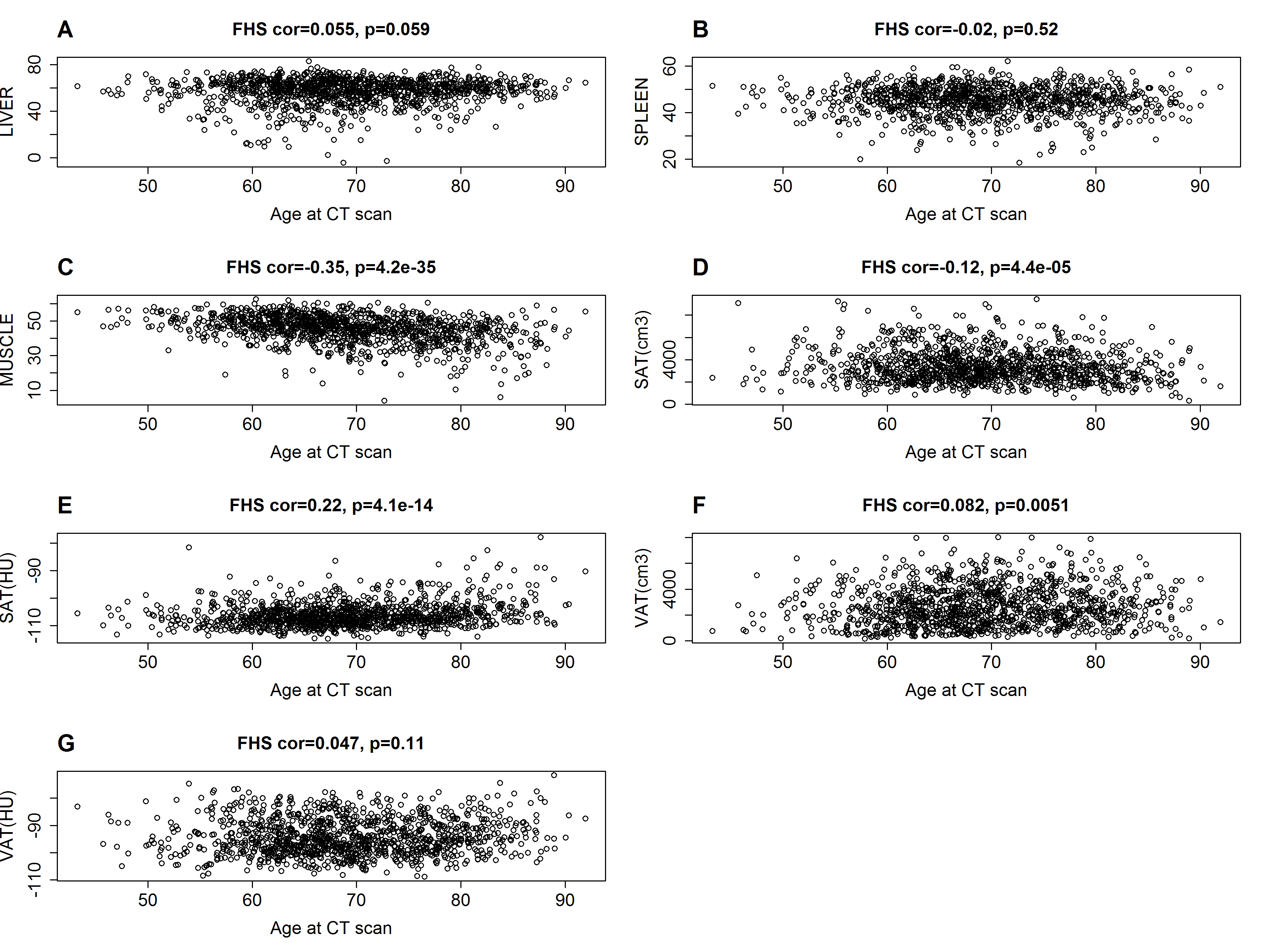


# Supplementary Figure 33. Marginal correlation analysis of CT-scan fatty liver associated and adipose tissue density with age-adjusted DNAm biomarkers in the FHS.

The columns correspond to age adjusted DNAm age based on Horvath (AgeAccelResidual) [24], age-adjusted DNAm age based on Hannum et al. (AgeAccelHannum) [27], and age-adjusted DNAm PhenoAge (AgeAccelPheno) [28], and AgeAccelGrim, . The rows correspond to computed tomography (CT) derived fatty liver associated and adipose tissue density markers. The columns report the available sample size (n) in the FHS, the robust correlation coefficient (bicor, based on the biweight midcorrelation coefficient [31]), and a p-value based on a linear mixed effect model that also adjusts for the pedigree structure (implemented in the R package "nlme"). The bicor correlation coefficients is color coded (blue to red) across its range of [-1, 1]. P-values are color-coded in green (light to dark green scale). We applied the correlation analysis to males and females, respectively, and combined the results via fixed effect models weighted by inverse variance (listed in the top rows, denoted as “ALL”).

Panel A lists the results using the entire FHS cohort available for the CT scan (N ~ 1200). Panel B lists the results limited to the individuals in the FHS test dataset (N ~290).

**
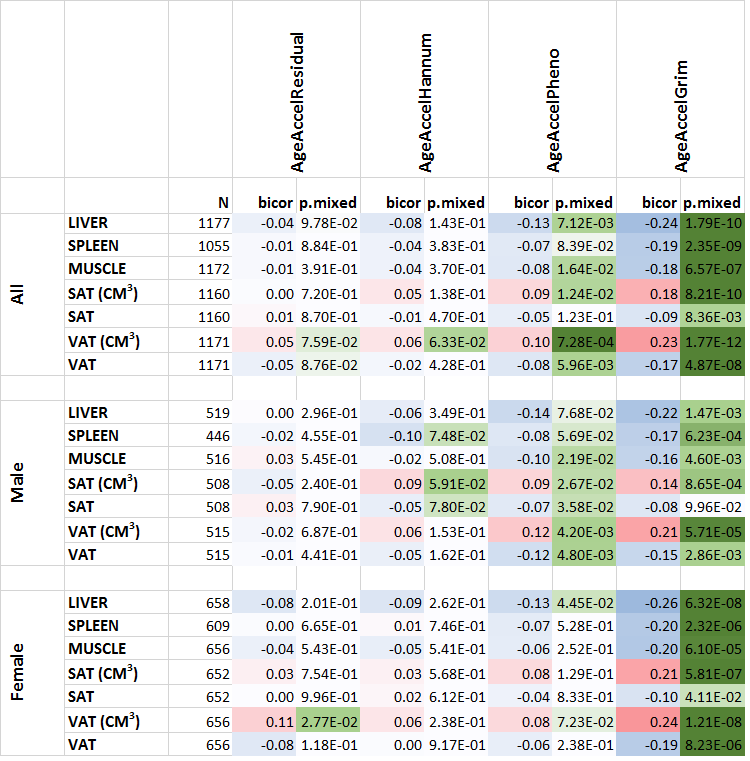
(A)**

(B)


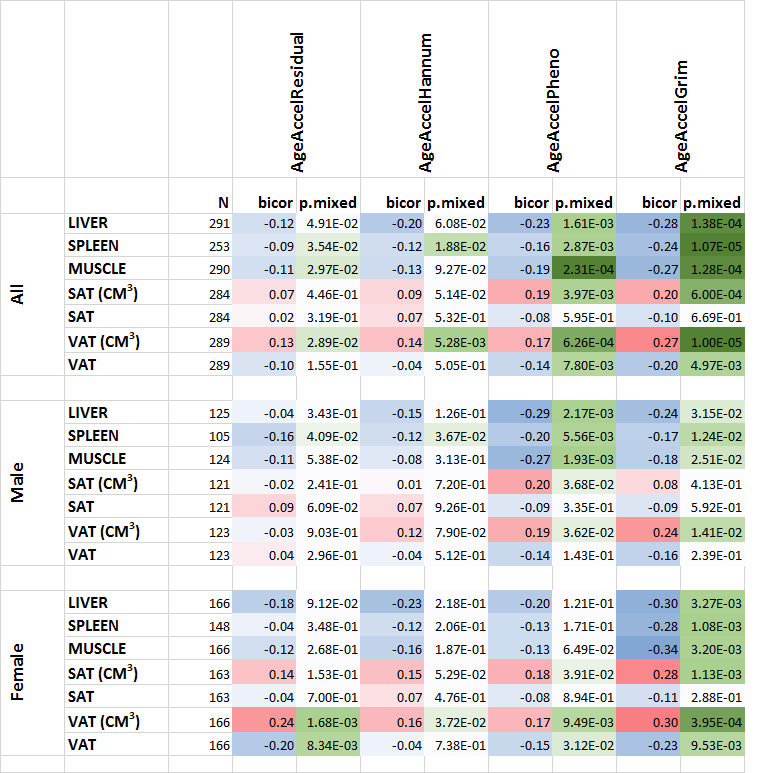

Supplement: Supplementary Figures [file aging-11-101684-s005.docx]
