# Supplementary material for: DNA methylation GrimAge strongly predicts lifespan and healthspan
Source: Aging (Albany NY). 2019 Jan 21;11(2):303–27. doi: 10.18632/aging.101684 (PMC6366976; doi:10.18632/aging.101684)
Supplement: Supplementary Tables [file aging-11-101684-s006.docx]

# SUPPLEMENTARY TABLES

# Supplementary Table 1. Characteristics of the Framingham Heart Study.

Description of the Framingham Heart Study (FHS) Offspring Cohort that was used to train and test

DNAm GrimAge. We assigned 70% of pedigrees to the training dataset and the remaining 30%

pedigrees to the test dataset.

|  | **Training** |  | **Test** |
| --- | --- | --- | --- |
|  | **N=1731** |  | **N=625** |
| **N_pedigree_** | 622 |  | 266 |
| **Female** | 54% |  | 53% |
| **Smoking** |  |  |  |
| Never | 41% |  | 37% |
| Former | 51% |  | 52% |
| Current | 8% |  | 10% |
| **Age** |  |  |  |
| exam 7 | 59.6±9.05 [53;67] |  | 60.3±8.59 [54;66] |
| exam 8 | 66.2±9.08 [59;73] |  | 66.9±8.64 [61;73] |
| **PACK years** | 13.8±20.17 [0;22] |  | 14.7±19.91 [0;23] |
| **Follow-up (years)** |  |  |  |
| since exam 7 | 14.5±1.69 [14;15.6] |  | 14.4±1.82 [13.9;15.6] |
| since exam 8 | 7.9±1.65 [7.5;8.9] |  | 7.7±1.78 [7.3;8.8] |

Quantitative variables are presented in the format of mean ± one standard deviation, SD and the interval: [25^th^, 75th] percentile.

Immunoassay array was profiled at exam 7;

DNA methylation array was profiled at exam 8.

# Supplementary Table 2. Multivariate regression model for estimating DNA GrimAge.

Coefficient values for computing the uncalibrated version of DNAm GrimAge based on the underlying covariates (rows): chronological age, sex (an indicator of female), and eight DNA methylation (DNAm) based variables (rows). The columns report the name of the covariate (e.g. DNAm based biomarker), its abbreviation, regression coefficient, and the number of underlying CpGs that underlie the surrogate biomarker (defined in stage 1). As noted, this model yields uncalibrated DNAm GrimAge. The finalized DNAm GrimAge is based on transforming the raw variable into a distribution in units of year (see **Methods**).

| **Covariate** | **Abbreviation** | **Coefficients** | **Number of CpGs** |
| --- | --- | --- | --- |
| DNAm adrenomedullin | DNAm ADM | 0.007903 | 186 |
| DNAm beta-2-microglobulin | DNAm B2M | 4.59E-7 | 91 |
| DNAm cystatin-C | DNAm Cystatin C | 3.5E-6 | 87 |
| DNAm growth differentiation factor 15 | DNAm GDF-15 | 0.000349 | 137 |
| DNAm leptin | DNAm Leptin | -7.3E-6 | 187 |
| DNAm plasminogen activator inhibitor 1 | DNAm PAI-1 | 2.56E-5 | 211 |
| DNAm tissue inhibitor metalloproteinases 1 | DNAm TIMP-1 | 0.000144 | 42 |
| DNAm pack years | DNAm PACKYRS | 0.030398 | 172 |
| Chronological age | Age | 0.030082 | -- |
| Female | Female | -0.22847 | -- |

# Supplementary Table 3. Comparing self-reported versus DNAm based estimates of smoking pack years wrt. predicting time-to-death.

Two Cox regression models for time-to-death (due to all-cause mortality) were used to evaluate the predictive power of self-reported smoking pack years and its DNAm based surrogate biomarker, respectively. The survival analysis was conducted across five datasets from four independent study cohorts (as detailed in the first column). The column report the variables, the sample size, the total number of deaths during the follow-up period, the hazard ratio associated with a 1 unit increase in the variable, and the corresponding Cox regression p-value. To compare the performance of the two variables, one should focus on the p-value as opposed to the hazard ratio (because the latter depends on the distribution of the underlying variable). Surprisingly, this comparative analysis indicates that the DNAm based surrogate biomarker of smoking pack years leads to a more significant (smaller) Cox regression p-value than that of the self-reported variable.

| **Data** | **Pack years** | **N** | **# death** | **HR** | **P** |
| --- | --- | --- | --- | --- | --- |
| FHS train | DNAm surrogate | 1729 | 219 | 1.04 | 8.10E-23 |
|  | Self-reported | 1729 | 219 | 1.02 | 1.12E-11 |
| FHS test | DNAm surrogate | 625 | 88 | 1.04 | 8.51E-5 |
|  | Self-reported | 625 | 88 | 1.01 | 2.13E-3 |
| WHI BA23 | DNAm surrogate | 2107 | 765 | 1.02 | 7.17E-12 |
|  | Self-reported | 2029 | 734 | 1.01 | 7.03E-12 |
| WHI EMPC | DNAm surrogate | 1972 | 505 | 1.03 | 2.1E-19 |
|  | Self-reported | 1904 | 484 | 1.02 | 6.4E-19 |
| InChianti | DNAm surrogate | 924 | 209 | 1.02 | 5.33E-4 |
|  | Self-reported | 924 | 209 | 1.01 | 1.78E-1 |

HR=hazard ratio

# Supplementary Table 4. Comparing ImmunoAssay versus DNAm based estimates of plasma proteins wrt. predicting time-to-death.

Two Cox regression models for time-to-death (due to all-cause mortality) were used to evaluate the predictive power of our study plasma proteins measured by ImmunoAssay versus its DNAm based surrogate biomarker, respectively. The survival analysis was adjusted for chronological age, center, and family structure, conducted in the FHS training (N/number of death =1729/219) and test (N/number of death=625/88) datasets, respectively. For each plasma protein, we list the hazard ratios (HR) and the corresponding Cox regression p-values of the protein levels based on ImmunoAssay measure and DNA methylation estimate, respectively. All the test variables were standardized based on the FHS training dataset such that each HR corresponds to an increase in one-standard deviation. To compare the performance of the two variables, one should focus on the p-value as opposed to the hazard ratio (because the latter depends on the distribution of the underlying variable).

The analysis for leptin levels was stratified by gender due to its highly correlation with gender.

|  |  | **HR*** | | **P-value** | |
| --- | --- | --- | --- | --- | --- |
| **Protein** | **data** | **ImmunoAssay** | **DNAm** | **ImmunoAssay** | **DNAm** |
| Adrenomedullin | training | 1.23 | 1.36 | 4.53E-4 | **3.85E-7** |
|  | test | 1.33 | 1.06 | 2.92E-2 | 7.23E-1 |
| Beta-2  Microglobulin | training | 1.31 | 1.51 | 7.42E-8 | 9.87E-8 |
|  | test | 1.22 | 1.94 | 1.33E-2 | **2.92E-3** |
| Cystatin C | training | 1.25 | 1.53 | 1.32E-4 | **1.44E-8** |
|  | test | 1.13 | 1.53 | 5.40E-2 | **1.92E-2** |
| GDF-15 | training | 1.24 | 1.20 | 3.82E-5 | 2.58E-3 |
|  | test | 1.83 | 1.63 | 6.86E-13 | 2.32E-2 |
| Leptin | training male | 1.28 | 2.23 | 1.03E-1 | **9.12E-6** |
|  | test male | 0.99 | 1.11 | 9.74E-1 | 6.83E-1 |
|  | training female | 0.93 | 0.97 | 4.81E-1 | 8.38E-1 |
|  | test female | 1.15 | 1.18 | 3.25E-3 | 5.91E-1 |
| PAI-1 | training | 1.14 | 1.31 | 3.95E-2 | **3.63E-6** |
|  | test | 1.19 | 1.37 | 7.42E-2 | **8.69E-4** |
| TIMP-1 | training | 1.33 | 2.61 | 1.38E-5 | **1.06E-8** |
|  | test | 1.29 | 3.30 | 1.71E-2 | **3.79E-4** |

*in units of one SD.

The p value based on a DNAm variable was marked in bold if it was more significant than the one based on the corresponding ImmunoAssay variable.

GDF-15=growth differentiation factor 15; PAI-1=plasminogen activation inhibitor 1; TIMP-1=tissue inhibitor metalloproteinase 1.

#

# Supplementary Table 5. Mortality prediction in the FHS based on DNAm- and observed versions of AgeAccelGrim.

The fact that AgeAccelGrim is a composite biomarker based on DNAm based surrogate biomarkers of plasma protein levels and smoking pack-years begs the question whether a predictor of lifespan based on observed values, i.e. observed plasma protein levels and self-reported smoking pack-years, outperforms its DNAm based analog? To our surprise, relatively little is gained by using observed values as will be shown in the following. Analogous to our construction of DNAm GrimAge, we used a Cox regression model to regress-time to-death on the observed plasma protein levels and self-reported pack-year in the training data (Methods). As before, the resulting mortality risk estimator (defined as linear combination of the observed biomarkers) was linearly transformed into units of years. The resulting predictor will be denoted as observed GrimAge and its age-adjusted version as observed AgeAccelGrim. The table reports the predictive accuracy for the two age acceleration measures (based on observed values versus DNAm based surrogates) when it comes to predicting time to death (due to all cause mortality). The table reports the sample size (N), the number of deaths during the follow up period, the hazard ratio associated with a 1 unit increase in the variable, the Z statistic, and the Cox regression p value. To compare the performance of the two variables, one should focus on the p-value as opposed to the hazard ratio (because the latter depends on the distribution of the underlying variable).

Rows correspond to the results in the training data and the test data of the FHS. An unbiased comparison in the test from the FHS reveals that the hazard ratio associated with observed AgeAccelGrim (HR=1.10, P=3.2E-7) is similar to that of its DNAm based analog AgeAccelGrim (HR= 1.12, P=8.6E-5).

The results in the training data of the FHS are biased and should be ignored.

| **FHS** | **AgeAccelGrim** | **N** | **# death** | **HR** | **Z** | **P** |
| --- | --- | --- | --- | --- | --- | --- |
| **Training**  **(N=1731)** | Observed | 1729 | 219 | 1.12 | 10.54 | 5.65E-26 |
|  | DNAm based | 1729 | 219 | 1.14 | 11.85 | 2.15E-32 |
| **Test**  **(N=625)** | Observed | 625 | 88 | 1.10 | 5.11 | 3.18E-7 |
|  | DNAm based | 625 | 88 | 1.12 | 3.93 | 8.55E-5 |

HR=hazard ratio.

# Supplementary Table 6. Heritability analysis of observed and DNAm based variables.

Robust polygenic model was performed to estimate heritability using the FHS test pedigree datasets. We estimated the heritability of observed and DNAm based AgeAccelGrim and it associated proteins. The observed AgeAccelGrim was defined in manuscript and the observed protein levels were based on ImmunoAssay measures. Additional analysis was applied to other epigenetic measures of age acceleration: age-adjusted DNAm PhenoAge (AgeAccelPheno)[28], age-adjusted DNAm age based on Hannum et al. (AgeAccelHannum) [27] and age adjusted DNAm age based on Horvath (AgeAccelResidual) [24].

|  | **Heritability** | | **P value** | |
| --- | --- | --- | --- | --- |
| **Trait** | **ImmunoAssay** | **DNAm** | **ImmunoAssay** | **DNAm** |
| AgeAccelGrim | 0.37 | 0.30 | 6.0E-3 | 2.2E-2 |
| AgeAccelPheno | NA | 0.11 | NA | 2.5E-1 |
| AgeAccelHannum | NA | 0.19 | NA | 7.7E-2 |
| AgeAccelerationResidual | NA | 0.44 | NA | 1.8E-3 |
| Adrenomedullin | 0.42 | 0.38 | 3.7E-3 | 2.9E-3 |
| Beta 2 microglobulin | 0.34 | 0.45 | 3.3E-3 | 2.4E-3 |
| Cystatin C | 0.32 | 0.09 | 2.9E-2 | 3.1E-1 |
| GDF-15 | 0.24 | 0.21 | 6.0E-2 | 9.1E-2 |
| Leptin male | 0.58 | 0.31 | 4.8E-2 | 1.8E-1 |
| Leptin females | 0.34 | 0.38 | 1.1E-1 | 1.3E-1 |
| PAI-1 | 0.34 | 0.51 | 7.1E-3 | 6.2E-4 |
| TIMP-1 | 0.31 | 0.24 | 2.0E-2 | 9.7E-2 |

GDF-15=growth differentiation factor 15; PAI-1=plasminogen activation inhibitor 1; TIMP-1=tissue inhibitor metalloproteinase 1;NA= not applicable.

# Supplementary Table 7. Stratification analysis of time-to-death among epigenetic measures of age acceleration.

Meta analysis for combining hazard ratios predicting time-to-death based on an epigenetic measure of age acceleration stratified by different subgroup. At each subgroup of each study set, the analysis was further stratified by racial group that we limited the analysis with number of events ≥20. The association results of four AgeAccle measures are listed below: AgeAccelGrim, age-adjusted DNAm PhenoAge (AgeAccelPheno) [28], age-adjusted DNAm age based on Hannum et al. [27], and age adjusted DNAm age based on Horvath [24]. The categories associated with BMI ranges are a) normal 18.5 -25 (normal), b) 25 to 30 (over), and c) >30 (obese). The categories I to IV associated with education attainment (EDU) are I) less than high school, II) high school degree, III) some college, and IV) college degree and above. The other abbreviations are HYPT (hypertension) and T2D (Type 2 diabetes). Each hazard ratio (HR) corresponds to a 1 year increase in the respective measure of age acceleration. The most significant meta analysis p-value across the 4 AgeAccel measures is marked in bold red at each row.

|  |  |  |  | **HR of AgeAccel** | | | | **P value of AgeAccel** | | | |
| --- | --- | --- | --- | --- | --- | --- | --- | --- | --- | --- | --- |
| **Group** | **level** | **N** | **Deaths** | **Grim** | **Pheno** | **Hannum** | **Horvath** | **Grim** | **Pheno** | **Hannum** | **Horvath** |
| **Age** | <65years | 3397 | 480 | 1.12 | 1.06 | 1.06 | 1.03 | **3.1E-34** | 5.8E-17 | 8.2E-8 | 7.4E-4 |
|  | >=65years | 3359 | 1310 | 1.09 | 1.04 | 1.03 | 1.01 | **4.0E-39** | 3.2E-20 | 5.7E-9 | 1.0E-2 |
| **BMI** | normal | 1504 | 443 | 1.10 | 1.05 | 1.05 | 1.01 | **7.7E-21** | 4.9E-10 | 6.6E-6 | 1.2E-1 |
|  | over | 2510 | 572 | 1.10 | 1.05 | 1.04 | 1.03 | **7.3E-27** | 4.6E-15 | 4.5E-8 | 6.7E-6 |
|  | obese | 3086 | 771 | 1.10 | 1.04 | 1.03 | 1.01 | **7.4E-26** | 3.1E-12 | 4.8E-5 | 1.7E-1 |
| **EDU** | I | 960 | 299 | 1.07 | 1.03 | 1.06 | 1.01 | **6.0E-8** | 7.1E-3 | 5.7E-6 | 2.7E-1 |
|  | II | 702 | 232 | 1.13 | 1.04 | 1.05 | 1.03 | **2.1E-12** | 3.9E-5 | 3.5E-4 | 5.3E-2 |
|  | III | 1565 | 508 | 1.09 | 1.04 | 1.03 | 1.01 | **5.9E-13** | 4.0E-8 | 4.0E-3 | 3.4E-1 |
|  | IV | 1374 | 349 | 1.12 | 1.05 | 1.04 | 1.01 | **4.5E-15** | 1.2E-7 | 6.5E-4 | 3.8E-1 |
| **HYPT** | No | 3988 | 785 | 1.11 | 1.04 | 1.03 | 1.02 | **3.6E-31** | 1.7E-10 | 1.4E-5 | 3.5E-2 |
|  | Yes | 3170 | 993 | 1.09 | 1.04 | 1.04 | 1.02 | **2.6E-36** | 2.5E-21 | 3.1E-10 | 2.6E-3 |
| **T2D** | No | 5726 | 1201 | 1.10 | 1.05 | 1.04 | 1.01 | **5.3E-49** | 3.1E-23 | 1.7E-8 | 1.8E-2 |
|  | Yes | 1009 | 310 | 1.09 | 1.04 | 1.04 | 1.02 | **3.6E-10** | 6.3E-7 | 4.4E-4 | 3.9E-2 |
| **Cancer** | No | 5089 | 1389 | 1.10 | 1.04 | 1.04 | 1.01 | **1.7E-45** | 8.5E-20 | 5.2E-10 | 2.2E-2 |
|  | Yes | 281 | 84 | 1.07 | 1.06 | 1.05 | 1.03 | 1.4E-2 | **1.6E-6** | 2.7E-3 | 1.9E-2 |

# Supplementary Table 8. Stratification analysis of time-to-CHD among epigenetic measures of age acceleration.

Meta analysis for combining hazard ratios predicting time-to-CHD based on an epigenetic measure of age acceleration stratified by different subgroup. At each subgroup of each study set, the analysis was further stratified by racial group that we limited the analysis with number of events ≥20. The association results of four AgeAccle measures are listed below: AgeAccelGrim, age-adjusted DNAm PhenoAge (AgeAccelPheno) [28], age-adjusted DNAm age based on Hannum et al. [27], and age adjusted DNAm age based on Horvath [24]. The categories associated with BMI ranges are a) normal 18.5 -25 (normal), b) 25 to 30 (over), and c) >30 (obese). The categories I to IV associated with education attainment (EDU) are I) less than high school, II) high school degree, III) some college, and IV) college degree and beyond. The other abbreviations are HYPT (hypertension) and T2D (Type 2 diabetes). Each hazard ratio (HR) corresponds to a 1 year increase in the respective measure of age acceleration. The most significant meta analysis p-value across the 4 AgeAccel measures is marked in bold red at each row.

|  |  |  |  | **HR of AgeAccel** | | | | **P value of AgeAccel** | | | |
| --- | --- | --- | --- | --- | --- | --- | --- | --- | --- | --- | --- |
| **Group** | **level** | **N** | **Deaths** | **Grim** | **Pheno** | **Hannum** | **Horvath** | **Grim** | **Pheno** | **Hannum** | **Horvath** |
| **Age** | <65years | 3343 | 370 | 1.10 | 1.04 | 1.03 | 1.02 | **8.5E-16** | 1.3E-5 | 4.1E-3 | 1.2E-1 |
|  | >=65years | 2561 | 584 | 1.06 | 1.02 | 1.02 | 1.01 | **7.2E-8** | 1.9E-3 | 2.7E-2 | 4.0E-1 |
| **BMI** | normal | 757 | 176 | 1.10 | 1.02 | 1.02 | 1.01 | **1.0E-7** | 4.7E-2 | 1.9E-1 | 7.2E-1 |
|  | over | 1872 | 285 | 1.06 | 1.03 | 1.01 | 1.01 | **2.7E-6** | 3.3E-4 | 2.9E-1 | 5.3E-1 |
|  | obese | 2668 | 450 | 1.08 | 1.02 | 1.02 | 1.01 | **4.3E-10** | 3.7E-3 | 1.7E-2 | 5.9E-1 |
| **EDU** | I | 270 | 85 | 1.07 | 1.01 | 1.04 | 1.00 | **9.8E-3** | 6.2E-1 | 1.3E-1 | 9.2E-1 |
|  | II | 459 | 140 | 1.08 | 1.01 | 1.01 | 0.99 | **3.8E-4** | 4.9E-1 | 4.2E-1 | 5.7E-1 |
|  | III | 1604 | 342 | 1.08 | 1.03 | 1.02 | 1.02 | **1.9E-8** | 1.5E-3 | 3.7E-2 | 1.3E-2 |
|  | IV | 746 | 184 | 1.08 | 1.03 | 1.01 | 0.99 | **4.0E-5** | 3.2E-3 | 4.9E-1 | 3.7E-1 |
| **HYPT** | No | 1888 | 351 | 1.08 | 1.02 | 1.01 | 1.01 | **1.1E-8** | 9.9E-3 | 2.7E-1 | 5.6E-1 |
|  | Yes | 2753 | 549 | 1.07 | 1.02 | 1.02 | 1.00 | **2.3E-11** | 5.7E-4 | 2.5E-2 | 6.4E-1 |
| **T2D** | No | 4515 | 557 | 1.07 | 1.02 | 1.02 | 1.01 | **2.8E-12** | 1.8E-4 | 5.7E-2 | 2.4E-1 |
|  | Yes | 650 | 159 | 1.06 | 1.02 | 1.02 | 1.01 | **1.4E-3** | 1.0E-1 | 1.8E-1 | 7.2E-1 |
| **Cancer** | No | 3950 | 776 | 1.08 | 1.02 | 1.02 | 1.01 | **2.5E-17** | 1.0E-5 | 8.1E-3 | 2.1E-1 |
|  | Yes | 176 | 37 | 1.07 | 1.02 | 1.00 | 0.97 | **8.9E-2** | 3.6E-1 | 8.8E-1 | 5.0E-1 |

# Supplementary Table 9. Distributions of epigenetic age acceleration measures.

We list the standard deviations and sample quantities of four epigenetic age acceleration measures: AgeAccelGrim, age-adjusted DNAm PhenoAge (AgeAccelPheno), age-adjusted DNAm age based on Hannum et al. (AgeAccelHannum), and age adjusted DNAm age based on Horvath (AgeAccelResidual). The estimates were stratified by cohort, racial group and visit (baseline and follow-up).

| **Group** | **AgeAccel** | **SD** | **1^th^** | **5^th^** | **10^th^** | **25^th^** | **median** | **75^th^** | **90^th^** | **95^th^** | **99^th^** |
| --- | --- | --- | --- | --- | --- | --- | --- | --- | --- | --- | --- |
| **FHS test** | | | | | | | | | | | |
|  | AgeAccelGrim | 3.81 | -7.40 | -5.48 | -4.41 | -2.60 | -0.47 | 2.12 | 5.06 | 7.02 | 10.59 |
|  | AgeAccelPheno | 6.45 | -15.02 | -10.05 | -7.95 | -4.01 | -0.15 | 4.03 | 7.84 | 10.68 | 16.78 |
|  | AgeAccelHannum | 4.87 | -11.47 | -7.76 | -6.10 | -3.20 | 0.00 | 3.22 | 6.13 | 7.93 | 10.92 |
|  | AgeAccelResidual | 5.30 | -12.03 | -7.82 | -6.19 | -3.20 | -0.12 | 3.28 | 6.27 | 8.57 | 14.23 |
| **WHI BA23** | | | | | | | | | | | |
| White | AgeAccelGrim | 3.75 | -7.52 | -5.83 | -4.79 | -3.02 | -0.92 | 1.60 | 4.68 | 6.78 | 9.35 |
|  | AgeAccelPheno | 6.10 | -13.45 | -9.83 | -8.18 | -4.54 | -0.67 | 3.21 | 7.19 | 9.73 | 14.05 |
|  | AgeAccelHannum | 4.64 | -10.96 | -7.22 | -5.65 | -3.08 | 0.01 | 3.01 | 6.00 | 7.77 | 11.08 |
|  | AgeAccelResidual | 5.05 | -10.22 | -7.21 | -5.75 | -2.77 | 0.01 | 3.48 | 6.48 | 8.54 | 13.53 |
| AfricanA | AgeAccelGrim | 4.06 | -7.10 | -5.08 | -3.88 | -1.89 | 0.56 | 3.07 | 6.41 | 8.57 | 11.28 |
|  | AgeAccelPheno | 7.22 | -17.80 | -10.79 | -8.19 | -3.98 | 0.40 | 4.64 | 8.94 | 12.76 | 18.79 |
|  | AgeAccelHannum | 5.34 | -14.44 | -8.99 | -7.30 | -4.05 | -0.75 | 2.52 | 5.94 | 7.80 | 10.13 |
|  | AgeAccelResidual | 6.06 | -13.81 | -9.06 | -7.05 | -3.25 | 0.19 | 3.72 | 7.40 | 9.66 | 15.70 |
| Hispanic | AgeAccelGrim | 3.28 | -6.69 | -5.15 | -4.41 | -2.53 | -0.64 | 1.30 | 3.79 | 5.20 | 9.75 |
|  | AgeAccelPheno | 5.82 | -11.53 | -8.63 | -6.64 | -3.46 | 0.41 | 4.45 | 8.31 | 10.59 | 14.42 |
|  | AgeAccelHannum | 4.42 | -8.33 | -6.27 | -4.97 | -1.80 | 0.93 | 3.82 | 6.79 | 8.10 | 11.87 |
|  | AgeAccelResidual | 4.46 | -10.12 | -7.61 | -6.03 | -3.83 | -0.56 | 2.45 | 4.80 | 6.24 | 10.96 |
| **WHI EMPC** | | | | | | | | | | | |
| White | AgeAccelGrim | 3.99 | -7.50 | -5.93 | -4.82 | -3.07 | -1.06 | 1.72 | 4.84 | 7.24 | 11.19 |
|  | AgeAccelPheno | 6.27 | -14.52 | -10.49 | -8.23 | -4.32 | -0.28 | 3.86 | 8.06 | 10.57 | 14.58 |
|  | AgeAccelHannum | 4.75 | -10.46 | -7.21 | -5.36 | -2.64 | 0.45 | 3.62 | 6.31 | 7.98 | 12.02 |
|  | AgeAccelResidual | 4.55 | -10.12 | -7.09 | -5.33 | -2.71 | 0.42 | 2.86 | 5.76 | 7.57 | 13.30 |
| AfricanA | AgeAccelGrim | 4.17 | -7.09 | -5.24 | -3.81 | -1.78 | 0.84 | 3.36 | 6.71 | 8.69 | 12.32 |
|  | AgeAccelPheno | 7.07 | -17.40 | -11.65 | -8.47 | -4.40 | 0.02 | 4.78 | 9.01 | 11.66 | 16.13 |
|  | AgeAccelHannum | 4.84 | -13.28 | -9.11 | -7.21 | -4.40 | -1.23 | 1.94 | 4.58 | 6.07 | 11.37 |
|  | AgeAccelResidual | 4.75 | -10.43 | -8.15 | -5.81 | -2.97 | -0.16 | 3.23 | 5.85 | 7.50 | 11.54 |
| Hispanic | AgeAccelGrim | 3.54 | -6.97 | -5.33 | -4.28 | -2.73 | -0.82 | 1.26 | 4.07 | 6.26 | 9.86 |
|  | AgeAccelPheno | 6.43 | -14.52 | -9.61 | -7.65 | -4.03 | 0.37 | 5.05 | 9.17 | 10.63 | 15.12 |
|  | AgeAccelHannum | 4.39 | -9.11 | -6.39 | -4.99 | -2.09 | 0.70 | 3.69 | 5.87 | 8.07 | 11.70 |
|  | AgeAccelResidual | 4.20 | -10.44 | -7.01 | -6.03 | -3.33 | -0.74 | 1.82 | 4.44 | 6.12 | 9.58 |
| **JHS** | | | | | | | | | | | |
|  | AgeAccelGrim | 4.81 | -8.59 | -6.34 | -5.29 | -3.26 | -0.81 | 2.39 | 6.78 | 9.49 | 13.61 |
|  | AgeAccelPheno | 6.10 | -13.85 | -9.76 | -7.50 | -3.98 | -0.20 | 4.02 | 7.70 | 9.95 | 15.15 |
|  | AgeAccelHannum | 3.50 | -7.82 | -5.41 | -4.20 | -2.17 | -0.16 | 2.13 | 4.37 | 5.77 | 9.67 |
|  | AgeAccelResidual | 4.47 | -10.32 | -7.01 | -5.55 | -3.01 | -0.08 | 2.92 | 5.73 | 7.32 | 10.70 |
| **InChianti** | | | | | | | | | | | |
| baseline | AgeAccelGrim | 4.29 | -6.91 | -5.81 | -5.08 | -2.90 | -0.53 | 2.12 | 5.76 | 8.38 | 12.23 |
|  | AgeAccelPheno | 4.40 | -10.22 | -6.76 | -5.35 | -2.84 | -0.12 | 2.67 | 5.55 | 6.82 | 10.66 |
|  | AgeAccelHannum | 4.90 | -11.32 | -7.55 | -5.93 | -3.26 | 0.02 | 2.70 | 5.59 | 7.68 | 13.61 |
|  | AgeAccelResidual | 4.91 | -10.95 | -6.97 | -5.84 | -3.27 | -0.26 | 3.20 | 6.04 | 8.54 | 12.66 |
| follow-up | AgeAccelGrim | 4.21 | -7.64 | -5.81 | -4.70 | -3.00 | -0.62 | 2.43 | 5.75 | 7.46 | 11.58 |
|  | AgeAccelPheno | 5.13 | -9.95 | -7.17 | -6.05 | -3.34 | -0.41 | 2.58 | 5.88 | 9.70 | 16.21 |
|  | AgeAccelHannum | 5.15 | -11.43 | -8.41 | -6.61 | -3.15 | 0.05 | 3.14 | 6.08 | 8.39 | 12.94 |
|  | AgeAccelResidual | 4.87 | -10.71 | -7.83 | -6.22 | -3.31 | -0.22 | 3.12 | 5.95 | 8.24 | 10.93 |

# Supplementary Table 10. Correlation analyses between DNAm based biomarkers and leukocyte telomere length.

The table presents the correlation coefficients (and corresponding p values) between leukocyte telomere length and age-adjusted versions of our DNAm based biomarkers (rows). The columns report the findings in the FHS test, WHI BA23 and JHS datasets. The last two columns report the findings of a fixed effects meta analysis weighted by inverse variance.

The columns report the correlation coefficients (denoted *r)* and the corresponding P value. The green color (light to dark) scale applies to unadjusted P values.

|  | Meta analysis  (N=2,702) | | FHS test  (N=245) | | WHI BA23  (N=818) | | JHS  (N=1,639) | |
| --- | --- | --- | --- | --- | --- | --- | --- | --- |
| Variable Name | r | P | r | P | r | P | r | P |
| AgeAccelGrim | -0.12 | 3.3E-10 | -0.12 | 5.7E-2 | -0.14 | 4.2E-5 | -0.11 | 1.0E-5 |
| adj.DNAm ADM | -0.002 | 9.0E-1 | 0.01 | 9.3E-1 | -0.09 | 1.1E-2 | 0.04 | 1.1E-1 |
| adj DNAm B2M | -0.07 | 1.8E-4 | -0.13 | 4.2E-2 | -0.13 | 3.2E-4 | -0.04 | 1.5E-1 |
| adj DNAm Cystatin C | -0.05 | 1.0E-2 | -0.11 | 7.6E-2 | -0.06 | 7.4E-2 | -0.03 | 1.8E-1 |
| adj DNAm GDF-15 | -0.10 | 3.4E-7 | -0.13 | 3.8E-2 | -0.13 | 1.1E-4 | -0.07 | 2.8E-3 |
| adj DNAm Leptin | 0.04 | 4.3E-2 | 0.06 | 3.9E-1 | -0.05 | 1.7E-1 | 0.08 | 1.2E-3 |
| adj DNAm PAI-1 | -0.10 | 5.1E-8 | -0.13 | 3.8E-2 | -0.13 | 1.5E-4 | -0.09 | 4.9E-4 |
| adj DNAm TIMP-1 | -0.05 | 9.8E-3 | -0.08 | 1.9E-1 | -0.07 | 4.1E-2 | -0.03 | 1.7E-1 |
| adj DNAm PACKYRS | -0.09 | 2.9E-6 | -0.05 | 4.2E-1 | -0.10 | 2.8E-3 | -0.09 | 3.6E-4 |

adj.=age adjusted.

Abbreviations of the DNAm based surrogate variables can be found in Supplementary Table 2.

# Supplementary Table 11. Impact of OMEGA3 on epigenetic age acceleration measures.

We investigated the impact of OMEGA-3 FATTY-ACIDS supplement on epigenetic age acceleration measures and age-adjusted DNAm proteins, using the FHS cohort. Of the cohort, 2174 participants were available for the self-report variable of OMEGA-3 intake. We studied the following epigenetic acceleration measures: AgeAccelGrim, age-adjusted DNAm PhenoAge (AgeAccelPheno)[28], age-adjusted DNAm age based on Hannum et al. [27], age adjusted DNAm age based on Horvath[24] (AgeAccelerationResidual), and intrinsic epigenetic age acceleration version of AgeAccelreationResidual (IEAA)[26]. Robust bicor-correlation analysis was performed to study marginal association, followed by linear mixed model analysis with P values adjusted for familial correlation and data status (training versus test). The variable of OMEGA-3 intake was log transformed, log (OMEGA-3+1), to improve its normality.

The columns report the bicor correlation coefficients, the corresponding P values and the P values of log OMEGA-3 as an independent variable in the linear mixed models. Additional analysis was conducted in the FHS test dataset.

| **Data** | **Epigenetic**  **biomarker** | **Bicor**  **correlation** | **Bicor**  **P value** | **Linear mixed**  **P value** |
| --- | --- | --- | --- | --- |
| **FHS**  **(N=2174)** | AgeAccelGrim | -0.108 | **4.6E-07** | **1.3E-05** |
|  | AgeAccelPheno | -0.008 | 7.1E-01 | 6.5E-01 |
|  | AgeAccelHannum | -0.023 | 2.9E-01 | 2.8E-01 |
|  | AgeAccelerationResidual | -0.052 | **1.6E-02** | 7.5E-02 |
|  | IEAA | -0.034 | 1.2E-01 | 2.4E-01 |
|  | adj.DNAm ADM | 0.017 | 4.3E-01 | 3.2E-01 |
|  | adj.DNAm B2M | -0.043 | **4.8E-02** | 3.2E-01 |
|  | adj.DNAm Cystatin C | -0.055 | **1.1E-02** | 1.7E-01 |
|  | adj.DNAm GDF-15 | -0.058 | **7.5E-03** | **5.9E-03** |
|  | adj.DNAm Leptin | 0.103 | **1.7E-06** | **6.0E-04** |
|  | adj.DNAm PAI-1 | -0.076 | **4.0E-04** | **4.7E-04** |
|  | adj.DNAm TIMP-1 | -0.069 | **1.5E-03** | **8.4E-04** |
| **FHS test (N=575)** | AgeAccelGrim | -0.055 | 1.9E-01 | 4.7E-01 |
|  | AgeAccelPheno | 0.001 | 9.9E-01 | 9.3E-01 |
|  | AgeAccelHannum | -0.063 | 1.4E-01 | 2.0E-01 |
|  | AgeAccelerationResidual | -0.167 | **6.4E-05** | **6.4E-03** |
|  | IEAA | -0.135 | **1.2E-03** | **2.7E-02** |
|  | adj.DNAm ADM | 0.036 | 3.9E-01 | 2.5E-01 |
|  | adj.DNAm B2M | -0.002 | 9.7E-01 | 7.5E-01 |
|  | adj.DNAm Cystatin C | -0.045 | 2.9E-01 | 9.4E-01 |
|  | adj.DNAm GDF-15 | -0.013 | 7.6E-01 | 4.7E-01 |
|  | adj.DNAm Leptin | 0.138 | **9.9E-04** | **1.1E-02** |
|  | adj.DNAm PAI-1 | -0.047 | 2.7E-01 | 7.1E-01 |
|  | adj.DNAm TIMP-1 | -0.014 | 7.4E-01 | 7.6E-01 |

# Supplementary Table 12. Multivariate regression analysis of AgeAccelGrim on CT-scan derived fatty liver and adipose tissue density in FHS.

The table presents the results from three multivariate regression models of AgeAccelGrim using the FHS individuals available for both DNA methylation data and computed tomography (CT) scan for fatty liver and adipose tissue density (N ~1000). In model I, we regressed AgeAccelGrim on fatty liver associated Hounsfield (HU) measures from CT-scan: liver HU, spleen HU and paraspinal muscle HU. In model II, we regressed AgeccelGrim on adipose tissue density measures from CT scan: abdominal subcutaneous (SAT) and visceral adipose tissue volume (VAT). In model III, we regressed AgeAccelGrim on liver, spleen, paraspinal muscle HU, SAT volume, and VAT volume. We performed linear mixed models for all the three models, adjusted for familiar correlations from pedigree, gender, and age at CT-scan.

| **Model*** | **Variable** | **Coefficient (SE)** | **P** |
| --- | --- | --- | --- |
| I | Liver | -5.0E-02 (1.45E-02) | **6.86E-04** |
|  | Spleen | -6.6E-02 (2.90E-02) | **2.38E-02** |
|  | Muscle | -3.2E-02 (1.98E-02) | 1.11E-01 |
|  | Female | -2.8E+00 (2.66E-01) | **4.47E-23** |
|  | BMI | 3.4E-02 (3.51E-02) | 3.36E-01 |
|  | Age at CT scan | -8.6E-03 (1.70E-02) | 6.15E-01 |
| II | SAT (cm^3^) | 1.5E-04 (1.56E-04) | 3.45E-01 |
|  | VAT (cm^3^) | 4.6E-04 (1.32E-04) | **5.54E-04** |
|  | Female | -2.7E+00 (3.47E-01) | **3.94E-14** |
|  | BMI | 5.0E-02 (4.79E-02) | 2.99E-01 |
|  | Age at CT scan | -7.6E-03 (1.55E-02) | 6.26E-01 |
| III | Liver | -4.2E-02 (1.57E-02) | **7.32E-03** |
|  | Spleen | -6.1E-02 (3.05E-02) | **4.67E-02** |
|  | Muscle | -3.6E-02 (2.05E-02) | 8.15E-02 |
|  | SAT (cm^3^) | -6.3E-05 (1.73E-04) | 7.15E-01 |
|  | VAT (cm^3^) | 2.4E-04 (1.53E-04) | 1.18E-01 |
|  | Female | -2.4E+00 (3.88E-01) | **1.22E-09** |
|  | BMI | 2.6E-02 (5.17E-02) | 6.19E-01 |
|  | Age at CT scan | -2.0E-02 (1.75E-02) | 2.56E-01 |
| *intercept estimates are not listed.  P values < 0.5 marked in bold. | | | |

# Supplementary Table 13. Multivariate regression analysis of age-adjusted DNA methylation based plasminogen inhibitor 1 (DNAm PAI-1) on CT-scan derived fatty liver and adipose tissue density in FHS.

The table presents the results from three multivariate regression models on DNA methylation based plasminogen inhibitor 1 (DNAm PAI-1) using the FHS individuals available for both DNA methylation data and computed tomography (CT) scan for fatty liver and adipose tissue density (N ~1000). In model I, we regressed age-adjusted DNAm PAI-1 on fatty liver associated Hounsfield (HU) measures from CT-scan: liver HU, spleen HU and paraspinal muscle HU. In model II, we regressed age-adjusted DNAm PAI-1 on adipose tissue density measures from CT scan: abdominal subcutaneous (SAT) and visceral adipose tissue volume (VAT). In model III, we regressed age-adjusted DNAm PAI-1 on liver, spleen, paraspinal muscle HU, SAT volume, and VAT volume. We performed linear mixed models for all the three models, adjusted for familiar correlations from pedigree, gender, and age at CT-scan.

| **Model*** | **Variable** | **Coefficient (SE)** | **P** |
| --- | --- | --- | --- |
| I | Liver | -9.0E+01 (1.15E+01) | **3.17E-14** |
|  | Spleen | -5.7E+00 (2.31E+01) | 8.05E-01 |
|  | Muscle | -1.0E+00 (1.58E+01) | 9.48E-01 |
|  | Female | -1.6E+03 (2.14E+02) | **9.93E-13** |
|  | BMI | 7.3E+01 (2.77E+01) | **9.00E-03** |
|  | Age at CT scan | 5.8E+00 (1.31E+01) | 6.61E-01 |
| II | SAT (cm^3^) | 3.1E-01 (1.21E-01) | **1.04E-02** |
|  | VAT (cm^3^) | 7.6E-01 (1.02E-01) | **4.22E-13** |
|  | Female | -1.2E+03 (2.71E+02) | **7.48E-06** |
|  | BMI | -1.5E+01 (3.72E+01) | 6.78E-01 |
|  | Age at CT scan | -1.3E+01 (1.17E+01) | 2.60E-01 |
| III | Liver | -6.8E+01 (1.22E+01) | **3.16E-08** |
|  | Spleen | 1.7E+01 (2.37E+01) | 4.78E-01 |
|  | Muscle | -4.6E+00 (1.60E+01) | 7.74E-01 |
|  | SAT (cm^3^) | 3.4E-01 (1.34E-01) | **1.18E-02** |
|  | VAT (cm^3^) | 6.4E-01 (1.19E-01) | **1.38E-07** |
|  | Female | -1.1E+03 (3.02E+02) | **1.99E-04** |
|  | BMI | -5.3E+01 (4.01E+01) | 1.90E-01 |
|  | Age at CT scan | -9.0E+00 (1.33E+01) | 5.00E-01 |
| *intercept estimates are not listed.  P values < 0.5 marked in bold. | | | |
