# Supplementary material for: DNA methylation GrimAge strongly predicts lifespan and healthspan
Source: Aging (Albany NY). 2019 Jan 21;11(2):303–27. doi: 10.18632/aging.101684 (PMC6366976; doi:10.18632/aging.101684)
Supplement: Supplementary References [file aging-11-101684-s007.docx]

# SUPPLEMENTARY REFERENCES

1. Dawber TR, Meadors GF and Moore FE, Jr. Epidemiological approaches to heart disease: the Framingham Study. Am J Public Health Nations Health. 1951; 41(3):279-281.

2. Kannel WB, Feinleib M, McNamara PM, Garrison RJ and Castelli WP. An investigation of coronary heart disease in families. The Framingham offspring study. Am J Epidemiol. 1979; 110(3):281-290.

3. Aryee MJ, Jaffe AE, Corrada-Bravo H, Ladd-Acosta C, Feinberg AP, Hansen KD and Irizarry RA. Minfi: a flexible and comprehensive Bioconductor package for the analysis of Infinium DNA methylation microarrays. Bioinformatics. 2014; 30(10):1363-1369.

4. Anonymous A. Design of the Women's Health Initiative clinical trial and observational study. The Women's Health Initiative Study Group. Control Clin Trials. 1998; 19(1):61-109.

5. Anderson G, Manson J, Wallace R, Lund B, Hall D, Davis S, Shumaker S, Wang C, Stein E and Prentice R. Implementation of the women's health initiative study design. Ann Epidemiol. 2003; 13(Suppl 9):S5-17.

6. Whitsel E. (2016). Epigenetic Mechanisms of PM-Mediated CVD Risk (WHI-EMPC; R01-ES020836). National Institutes of Health US Department of Health and Human Services Research Portfolio Online Reporting Tools.

7. Teschendorff AE, Marabita F, Lechner M, Bartlett T, Tegner J, Gomez-Cabrero D and Beck S. A beta-mixture quantile normalization method for correcting probe design bias in Illumina Infinium 450 k DNA methylation data. Bioinformatics. 2013; 29(2):189-196.

8. Johnson WE, Rabinovic A and Li C. Adjusting batch effects in microarray expression data using Empirical Bayes methods. Biostatistics. 2007; 8(1):118-127.

9. Patterson RE, Kristal AR, Tinker LF, Carter RA, Bolton MP and Agurs-Collins T. Measurement characteristics of the Women’s Health Initiative food frequency questionnaire. Annals of epidemiology. 1999; 9(3):178-187.

10. Taylor HA, Jr., Wilson JG, Jones DW, Sarpong DF, Srinivasan A, Garrison RJ, Nelson C and Wyatt SB. Toward resolution of cardiovascular health disparities in African Americans: design and methods of the Jackson Heart Study. Ethn Dis. 2005; 15(4 Suppl 6):S6-4-17.

11. Moore AZ, Hernandez DG, Tanaka T, Pilling LC, Nalls MA, Bandinelli S, Singleton AB and Ferrucci L. Change in Epigenome-Wide DNA Methylation Over 9 Years and Subsequent Mortality: Results From the InCHIANTI Study. The journals of gerontology Series A, Biological sciences and medical sciences. 2015.

12. Wong HK, Cheung TT and Cheung BM. Adrenomedullin and cardiovascular diseases. JRSM Cardiovasc Dis. 2012; 1(5).

13. Larrayoz IM, Ferrero H, Martisova E, Gil-Bea FJ, Ramirez MJ and Martinez A. Adrenomedullin Contributes to Age-Related Memory Loss in Mice and Is Elevated in Aging Human Brains. Front Mol Neurosci. 2017; 10:384.

14. Liabeuf S, Lenglet A, Desjardins L, Neirynck N, Glorieux G, Lemke HD, Vanholder R, Diouf M, Choukroun G, Massy ZA and European Uremic Toxin Work G. Plasma beta-2 microglobulin is associated with cardiovascular disease in uremic patients. Kidney Int. 2012; 82(12):1297-1303.

15. Smith LK, He Y, Park JS, Bieri G, Snethlage CE, Lin K, Gontier G, Wabl R, Plambeck KE, Udeochu J, Wheatley EG, Bouchard J, Eggel A, et al. beta2-microglobulin is a systemic pro-aging factor that impairs cognitive function and neurogenesis. Nat Med. 2015; 21(8):932-937.

16. Ferguson TW, Komenda P and Tangri N. Cystatin C as a biomarker for estimating glomerular filtration rate. Curr Opin Nephrol Hypertens. 2015; 24(3):295-300.

17. van der Laan SW, Fall T, Soumare A, Teumer A, Sedaghat S, Baumert J, Zabaneh D, van Setten J, Isgum I, Galesloot TE, Arpegard J, Amouyel P, Trompet S, et al. Cystatin C and Cardiovascular Disease: A Mendelian Randomization Study. J Am Coll Cardiol. 2016; 68(9):934-945.

18. Paterson RW, Bartlett JW, Blennow K, Fox NC, Alzheimer's Disease Neuroimaging I, Shaw LM, Trojanowski JQ, Zetterberg H and Schott JM. Cerebrospinal fluid markers including trefoil factor 3 are associated with neurodegeneration in amyloid-positive individuals. Translational psychiatry. 2014; 4:e419.

19. Fujita Y, Taniguchi Y, Shinkai S, Tanaka M and Ito M. Secreted growth differentiation factor 15 as a potential biomarker for mitochondrial dysfunctions in aging and age-related disorders. Geriatr Gerontol Int. 2016; 16 Suppl 1:17-29.

20. Maioli S, Lodeiro M, Merino-Serrais P, Falahati F, Khan W, Puerta E, Codita A, Rimondini R, Ramirez MJ, Simmons A, Gil-Bea F, Westman E, Cedazo-Minguez A, et al. Alterations in brain leptin signalling in spite of unchanged CSF leptin levels in Alzheimer's disease. Aging cell. 2015; 14(1):122-129.

21. Cesari M, Pahor M and Incalzi RA. Plasminogen activator inhibitor-1 (PAI-1): a key factor linking fibrinolysis and age-related subclinical and clinical conditions. Cardiovasc Ther. 2010; 28(5):e72-91.

22. Ashutosh, Chao C, Borgmann K, Brew K and Ghorpade A. Tissue inhibitor of metalloproteinases-1 protects human neurons from staurosporine and HIV-1-induced apoptosis: mechanisms and relevance to HIV-1-associated dementia. Cell Death Dis. 2012; 3:e332.

23. Houseman E, Accomando W, Koestler D, Christensen B, Marsit C, Nelson H, Wiencke J and Kelsey K. DNA methylation arrays as surrogate measures of cell mixture distribution. BMC Bioinformatics. 2012; 13(1):86.

24. Horvath S. DNA methylation age of human tissues and cell types. Genome biology. 2013; 14(10):R115.

25. Horvath S and Levine AJ. HIV-1 Infection Accelerates Age According to the Epigenetic Clock. J Infect Dis. 2015; 212(10):1563-1573.

26. Horvath S, Gurven M, Levine ME, Trumble BC, Kaplan H, Allayee H, Ritz BR, Chen B, Lu AT, Rickabaugh TM, Jamieson BD, Sun D, Li S, et al. An epigenetic clock analysis of race/ethnicity, sex, and coronary heart disease. Genome biology. 2016; 17(1):171.

27. Hannum G, Guinney J, Zhao L, Zhang L, Hughes G, Sadda S, Klotzle B, Bibikova M, Fan JB, Gao Y, Deconde R, Chen M, Rajapakse I, et al. Genome-wide methylation profiles reveal quantitative views of human aging rates. Molecular cell. 2013; 49(2):359-367.

28. Levine ME, Lu AT, Quach A, Chen BH, Assimes TL, Bandinelli S, Hou L, Baccarelli AA, Stewart JD, Li Y, Whitsel EA, Wilson JG, Reiner AP, et al. An epigenetic biomarker of aging for lifespan and healthspan. Aging. 2018; 10(4):573-591.

29. Zhang Y, Wilson R, Heiss J, Breitling LP, Saum KU, Schottker B, Holleczek B, Waldenberger M, Peters A and Brenner H. DNA methylation signatures in peripheral blood strongly predict all-cause mortality. Nat Commun. 2017; 8:14617.

30. Horvath S and Levine AJ. HIV-1 infection accelerates age according to the epigenetic clock. J Infect Dis. 2015.

31. Langfelder P and Horvath S. WGCNA: an R package for weighted correlation network analysis. BMC bioinformatics. 2008; 9:559.
